# Different binding modalities of quercetin to inositol-requiring enzyme 1 of *S. cerevisiae* and human lead to opposite regulation

**DOI:** 10.1038/s42004-023-01092-0

**Published:** 2024-01-05

**Authors:** S. Jalil Mahdizadeh, Johan Grandén, Diana Pelizzari-Raymundo, Xavier Guillory, Antonio Carlesso, Eric Chevet, Leif A. Eriksson

**Affiliations:** 1 Department of Chemistry and Molecular Biology, University of Gothenburg, 405 30 Göteborg, Sweden; 2 INSERM U1242, Université de Rennes, Rennes, France; 3 Centre de Lutte contre le Cancer Eugène Marquis, Rennes, France; 4 Univ Rennes, CNRS, ISCR – UMR 6226, F-35000 Rennes, France; 5 Department of Pharmacology, Sahlgrenska Academy, University of Gothenburg, SE-405 31 Gothenburg, Sweden

**Keywords:** Molecular dynamics, RNA-binding proteins, Enzyme mechanisms, Computational chemistry, Target validation

## Abstract

The flavonoid Quercetin (Qe) was identified as an activator of Inositol-requiring enzyme 1 (IRE1) in *S. cerevisiae* (*sc*Ire1p), but its impact on human IRE1 (*h*IRE1) remains controversial due to the absence of a conserved Qe binding site. We have explored the binding modes and effect of Qe on both *sc*Ire1p and *h*IRE1 dimers using in silico and in vitro approaches. The activation site in *sc*Ire1p stably accommodates both Qe and its derivative Quercitrin (Qi), thus enhancing the stability of the RNase pocket. However, the corresponding region in *h*IRE1 does not bind any of the two molecules. Instead, we show that both Qe and Qi block the RNase activity of *h*IRE1 in vitro, with sub-micromolar IC_50_ values. Our results provide a rationale for why Qe is an activator in *sc*Ire1p but a potent inhibitor in *h*IRE1. The identification of a new allosteric site in *h*IRE1 opens a promising window for drug development and UPR modulation.

## Abstract (web summary)

Inositol requiring enzyme 1 (IRE1) is a conserved protein kinase involved in the endoplasmic reticulum secretory pathway, however, IRE1 from *S. cerevisiae* and human show opposite responses to flavonoid quercetins. Here, the authors apply in silico and in vitro approaches to reveal the different binding modes of quercetins on IRE1, providing molecular insight into their opposite effects on *S. cerevisiae* and humans.

## Introduction

The endoplasmic reticulum (ER) is the most abundant cellular organelle in Eukaryotes and the gateway to the secretory pathway^1^. The ER plays an important role in secretory and transmembrane protein productive folding, accounting for about 1/3 of the cellular proteome. When the protein folding demand exceeds the ER capacity, ER homeostasis is disrupted, and ER stress is triggered. To cope with ER stress, the unfolded protein response (UPR) is activated^2^ and aims to restore ER protein homeostasis^3^. The UPR is transduced by three ER-resident transmembrane proteins in eukaryotes, namely the protein kinase RNA-like endoplasmic reticulum kinase (PERK), the activating transcription factor 6 (ATF6), and the Inositol requiring enzyme 1α (IRE1α, hereafter referred to as IRE1)^4^. PERK is a kinase that phosphorylates the eukaryotic initiation factor 2 alpha (eIF2α) in response to ER stress. Phosphorylation of eIF2α results in a global attenuation of protein synthesis in the cell, which reduces the protein-folding load on the ER. However, some specific mRNAs, including activating transcription factor 4 (ATF4), are preferentially translated under these conditions. ATF4 upregulates genes involved in amino acid metabolism, antioxidant responses, and apoptosis, among others^5^. ATF6 is a transmembrane protein in the ER that is transported to the Golgi apparatus and cleaved upon ER stress. The cleaved ATF6 fragment, ATF6(N), enters the nucleus and acts as a transcription factor. ATF6(N) upregulates the expression of ER chaperones, ERAD components, and other genes involved in protein folding and quality control to enhance the ER’s protein processing capacity^6^.

IRE1 is the most evolutionary conserved of the three sensors belonging to the family of protein kinases and is found from Yeast to Humans. It is structurally composed of an *N*-terminal sensor domain in the ER lumen, a transmembrane region, and a CDK2-like serine/threonine kinase domain fused to a unique C-terminal ribonuclease (RNase) domain in the cytosol^4^. Coordinated dissociation of the ER chaperone BiP/GRP78 and direct binding to misfolded proteins to the luminal domain promotes high-order oligomerization of IRE1, trans-autophosphorylation of the cytosolic domain leading to the activation RNase domain, and to the subsequent non-conventional splicing of XBP1 mRNA and Regulated IRE1 Dependent Decay of RNA^7,8^. The importance of IRE1 signaling has been shown in a wide variety of diseases such as cancer, diabetes, and Alzheimer’s disease, and IRE1 modulation has emerged as a novel route for disease intervention^9,10^. This has led to the development of a wide variety of small molecules that can either activate or inhibit the IRE1 signaling pathway by binding to either the RNase or kinase domain of IRE1^11,12^.

**Fig. 1:**
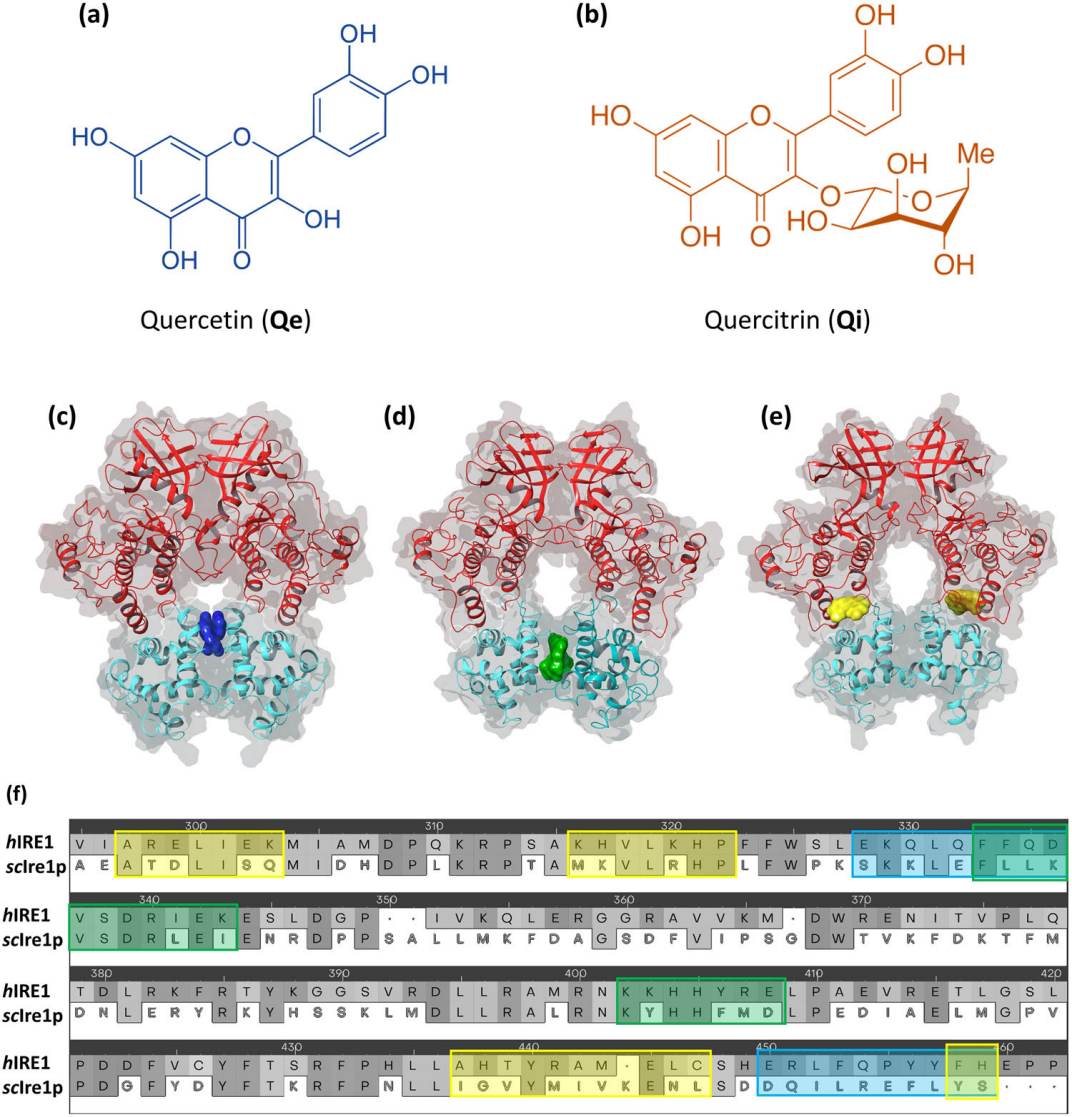
The ligands under study, binding sites of the IRE1 dimers, *h*IRE1 vs. *sc*Ire1p sequence alignment. The chemical structures of **a** quercetin (Qe) and **b** quercitrin (Qi) molecules. **c** Binding Site 1: the region where co-crystalized Quercetin (Qe) molecules bind in *sc*Ire1p back-to-back dimer (pdb id:3LJ0); **d** Binding Site 2: the region where Quercitrin (Qi) molecules can bind in *h*IRE1 back-to-back dimer (pdb id:4YZC) predicted in silico using peptidomimetic approach. **e** Binding Site 3: a new potential binding pocket where Qe is predicted to bind in *h*IRE1 back-to-back dimer (pdb id:4YZC) identified in silico using the FPocket tool. The Kinase and RNase domains of IRE1 dimers are shown in red and cyan colors, respectively. **f** Part of the pairwise sequence alignment between *h*IRE1 and *sc*Ire1p showing the residue composition of the binding sites evaluated in this study. The blue, green, and yellow boxes are associated with binding sites 1, 2, and 3, respectively.

In previous studies, peptide fragments derived from the IRE1 kinase domain sequence were identified to inhibit IRE1 activity by binding to the kinase domain^13,14^. Based on a similar approach, using a series of peptides derived from the IRE1 RNase domain, small molecules were identified to bind with high affinity towards the IRE1 RNase domain within the back-to-back dimer interface region^15^. This led to the identification of Quercitrin (Qi, Fig. 1b) as a novel inhibitor of human IRE1 (*h*IRE1) RNase activity. Quercitrin is a derivative of Quercetin (Qe, Fig. 1a), which was previously reported to be an activator of *S. cerevisiae* IRE1 (*sc*Ire1p)^16^ which co-crystallized in complex with *sc*Ire1p in the back-to-back dimer conformation (Fig. 1c). So far, Qe has not been co-crystallized in complex with *h*IRE1 and its impact on *h*IRE1 signaling remains very controversial.

In this study, we explored the ability of Qe and Qi to bind to both *sc*Ire1p and *h*IRE1 back-to-back dimers (Fig. 1c–e) using different in silico approaches. Computational modeling allowed us to explain the observed Qe-induced activation of *sc*Ire1pand the inhibitory effects and allosteric nature of Qe and Qi on *h*IRE1, the latter being also validated in vitro, and corroborating the dimer disrupting capability of both Qi and Qe. The opposite modulation of *h*IRE1 and *sc*Ire1p by Qe and Qi molecules is primarily influenced by significant divergence in the amino acid sequence identities of their respective binding sites (Fig. 1f) which will be discussed in the following section. The results of this study clarify the impact of sequence differences of *sc*Ire1p and *h*IRE1, in the selectivity of binding and interaction of Qe and Qi. Moreover, the identification of a new allosteric site in the linker region of *h*IRE1 connecting the kinase and RNase domains opens additional possibilities for therapeutic intervention.

## Results and discussion

### Binding site *sc-1* of *sc*Ire1p dimers enhance dimer stability

**Fig. 2:**
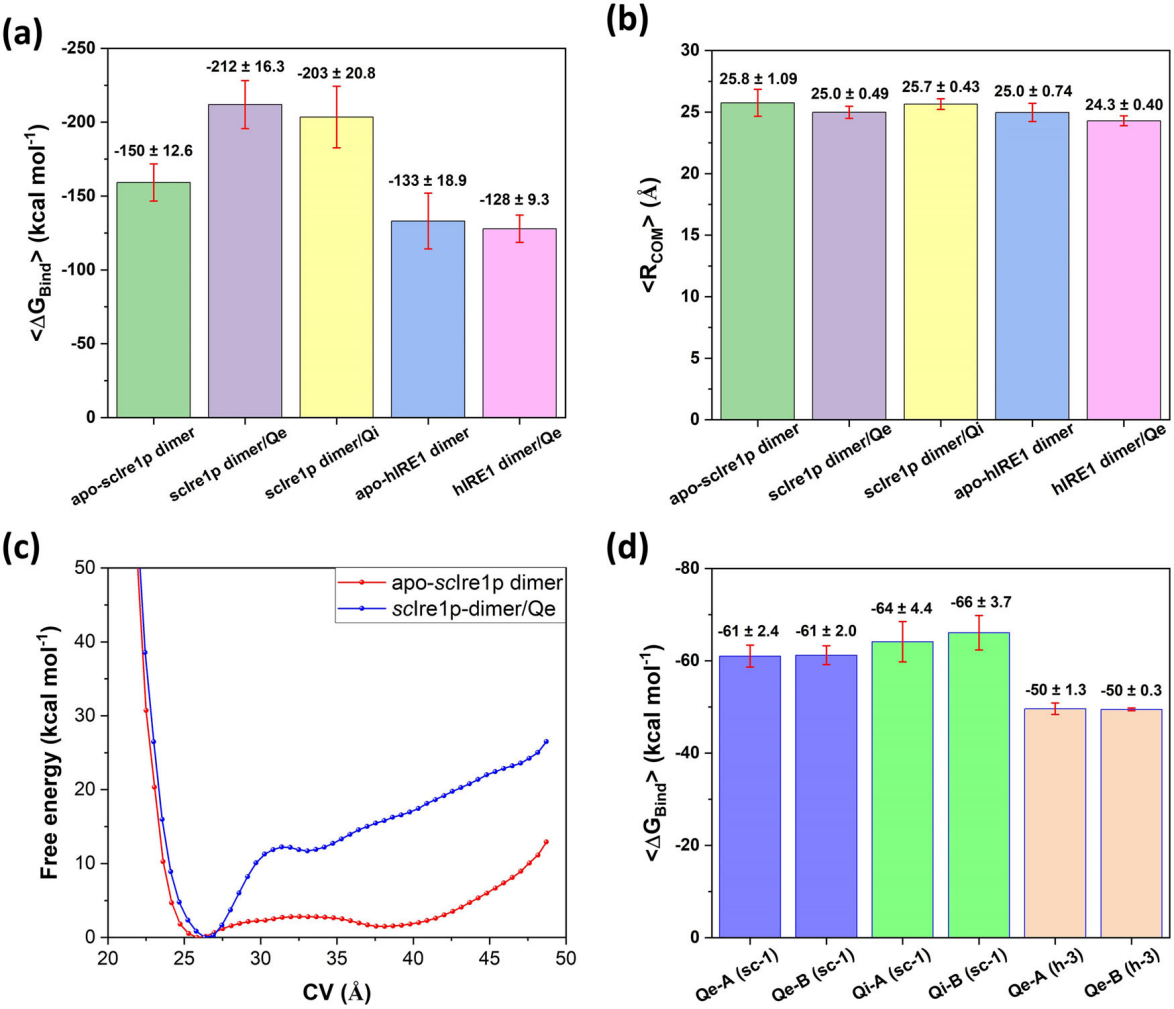
Free energies of binding of Qe and Qi, and their impact on dimer interaction energies and distances between the RNase domains in *h*IRE1 and *sc*Ire1p. **a** Influence of Qe and Qi molecules on the free energy of binding between two monomers in the *sc*Ire1p and *h*IRE1 dimers. **b** Average distance between center of mass <*R*_COM_> of RNase domains along with standard deviation values in apo-IRE1 dimers and those bound to Qe and Qi molecules in site *sc-1*. **c** Free energy profiles for the interaction between two RNase domains in apo- *sc*Ire1p dimer (red) and *sc*Ire1p dimer bound to Qe molecules (blue) in site *sc-1* as function of the distance between the centers of mass of the RNase domains, *R*_COM_ (CV). **d** Average free energy of binding values <Δ*G*_Bind_> for the Qe and Qi molecules at different binding sites in the *sc*Ire1p and *h*IRE1 dimers. The error bars are standard deviations and are shown as capped red vertical lines.

As a first step, the docking methodology was verified by benchmarking the docking of Qe towards the co-crystallized structure of *sc*Ire1p, as outlined in the Methods section (Supplementary Fig. 1 and Supplementary Table 1). This was followed by docking of Qi to the same site *sc-1*, followed by MD and BPMD simulations, and evaluation of computed binding affinities, for both Qe and Qi (Supplementary Fig. 2). It is concluded that both ligands bind strongly, the interaction is stable, and that the preferred protein-ligand stoichiometry is 2:2 (Supplementary Discussion). Next, we explored the impact of Qe and Qi binding to the *sc-1* binding site, on the dynamics and structural properties of the RNase domain of *sc*Ire1p dimers. To do so, 300 ns MD simulations were conducted in triplicate on the apo dimer, and the results were compared to those obtained with either Qe or Qi ligands bound to the dimers (Supplementary Discussion; Supplementary Fig. 2). The average free energy of binding <ΔG_Bind_> between the protein-protein interface of the two monomers in the apo-*sc*Ire1p dimer and those with Qe or Qi molecules bound to the *sc-1* site indicated that the former is −150 kcal mol^−1^ which is 52.8 and 44.4 kcal mol^−1^ weaker than the corresponding values obtained with Qe or Qi present (−211.9 and −203.5 kcal mol^−1^, respectively) (Fig. 2a). This implies that the Qe and Qi molecules clearly enhance the affinity of the protomers toward each other, leading to formation of a more stable protein complex. This stabilization is also reflected by the average fluctuation of the distance between the centers of mass of the two RNase domains, <*R*_COM_> (983–1115 in *sc*Ire1p), for the protomers during the triplicate 300 ns MD simulations (900 MD snapshots) (Fig. 2b). As is clearly indicated, while the <*R*_COM_> values are almost identical (25-26 Å), the average standard deviation of <*R*_COM_> in the *sc*Ire1p dimer bound to Qe/Qi molecules (0.49 and 0.43 Å, respectively) is significantly lower than the corresponding value in the apo dimer (1.09 Å), thus indicating a more stable RNase dimer and the subsequent increase in activity.

To further document this point, a series of well-tempered metadynamics (WT-MetaD) simulations were furthermore conducted to evaluate the effect of Qe, bound to the *sc-1* site, on the dynamics and interaction profile of the two RNase domains in the *sc*Ire1p dimer (Supplementary Discussion). The WT-MetaD simulations were performed in duplicate for two systems: the *sc*Ire1p dimer with Qe molecules bound to *sc-1*, and the apo- *sc*Ire1p dimer. The collective variable (CV) is the distance between the centers of mass of the two RNase domains (*R*_COM_) (Fig. 2c). The free energy profile for the interaction between the two RNase domains in the apo-*sc*Ire1p dimer and *sc*Ire1p dimer bound to Qe differ significantly. In the presence of Qe, the interaction between the two RNase domains is markedly enhanced compared to apo- *sc*Ire1p. This is in agreement with the results observed from free energy calculations (Fig. 2a), and <*R*_COM_> fluctuation evaluation (Fig. 2b). A video showing the RNase opening/closing cycles during the WT-MetaD trajectory is available in the associated Zenodo page (see Data availability). These results verify the formation of a more stable binding site for XBP1 mRNA when Qe is bound to *sc-1*, and support the experimental findings that Qe (and likely Qi) acts as an activator in the *sc*Ire1p dimer^16^.

### The activator binding site in *h*IRE1 dimers (*h-1*) is not stable

**Fig. 3:**
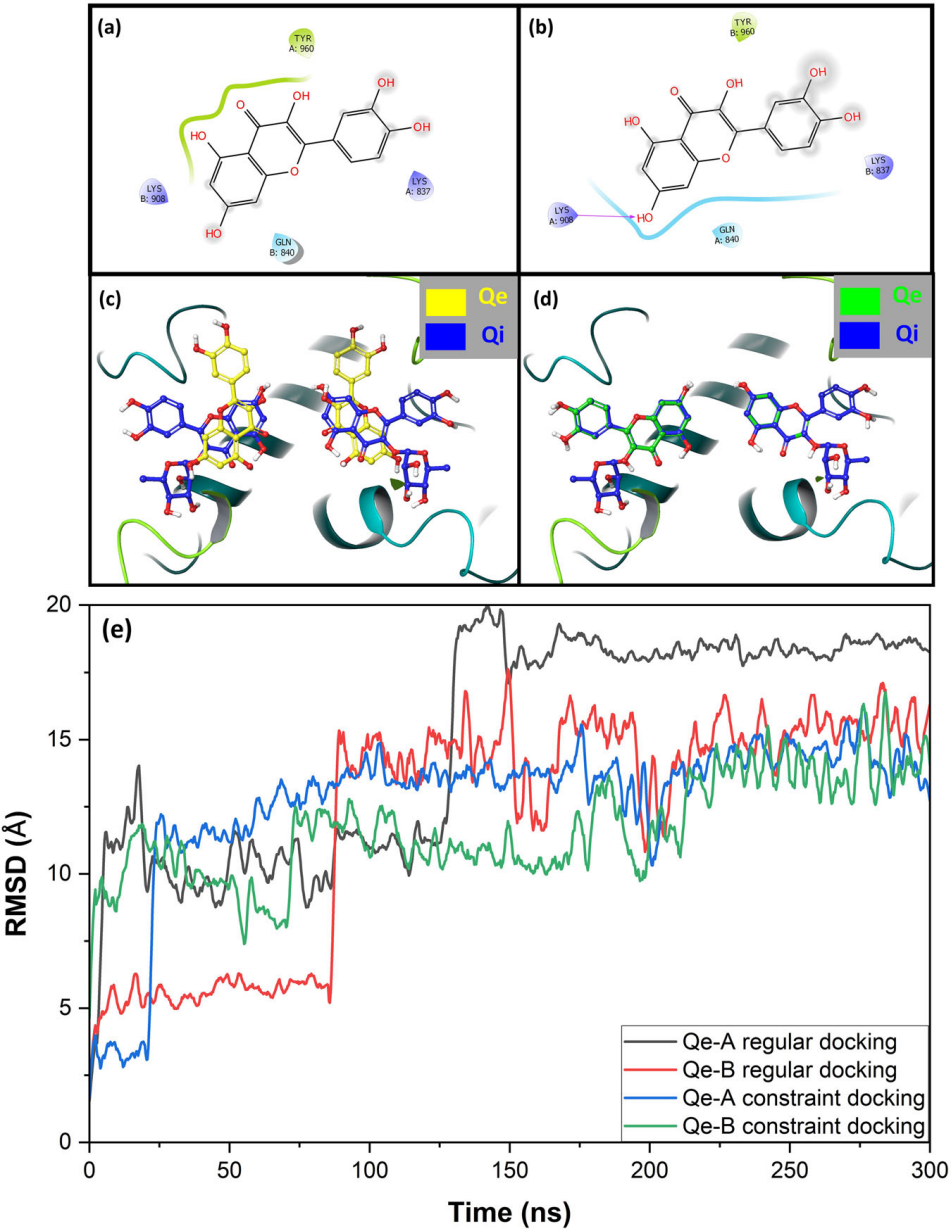
Docking and interactions of Qe in sites *h-1* and *h-2*. The closest residues from **a** Qe-A and **b** Qe-B molecules after *sc*Ire1p /*h*IRE1 superposition were used to generate the receptor grid boxes for binding *h-1*. The binding mode of Qe molecules docked into binding site *h-2* using **c** regular molecular docking (yellow) and **d** constraint molecular docking (green) calculations, superimposed on the Qi ligands (blue). **e** The high value of RMSD graphs of Qe molecules during 300 MD simulations indicate that Qe is not stable in *h-2*.

We next investigated the binding ability of Qe and Qi to the same region as *sc-1*, but on *h*IRE1 (*h-1* site) using molecular docking calculations. To generate a proper grid box for binding to *h-1*, the structures of *sc*Ire1p (3LJ0) and *h*IRE1 (4YZC) dimers were aligned and superimposed. The co-crystallized Qe molecules were transferred from *sc*Ire1p (binding site *sc-1*) to *h*IRE1, and the nearest residues identified for grid box generation (Fig. 3a, b). Qe and Qi were then docked using flexible docking into the *h-1* binding site. However, the calculations did not result in any relevant binding poses for neither Qe nor Qi. The fact that Qe has not been observed to be an activator in *h*IRE1, whereas it is an activator in *sc*Ire1p, is in line with these findings and can be explained by the significantly different sequence identities (<16%) of the proteins in this region, Fig. 1f.

### Crowding of the *h-2* site disrupts *h*IRE1 dimers

The *h-2* binding site (Fig. 1d) was recently identified in the *h*IRE1 dimer (pdb id: 4YZC)^15^. This binding site was identified through in silico peptidomimetic studies and yielded a set of FDA-approved compounds that could bind into the site, disrupt the dimer at the RNase interface, and eventually block the RNase activity of *h*IRE1, as evidenced by a series of in vitro experiments^15^. Interestingly, Qi was one of the FDA-approved compounds found to acts as an inhibitor through an *h*IRE1 dimer disruption mechanism of action. Amarasinghe et al.^15^. showed that Qi could be perfectly docked into the *h-2* site with the highest docking score and free energy of binding values among all the FDA compounds evaluated. Qi forms strong interactions with Arg912, Gln843, Gln840, Lys908, His909, and Asp847, which results in a high binding stability within this new allosteric site. Molecular dynamics and binding pose metadynamics simulations further confirmed the stability of Qi on the *h2* site. Herein, we address the question if Qe, which shares strong structural similarities with Qi, also binds to *h-2,* and inhibits IRE1 RNase activity. Regular molecular docking calculations showed that the binding modes of Qe and Qi are very different within *h-2* (Fig. 3c). The docking score values are of −7.5 and −7.1 kcal mol^−1^ for Qe bound to each of the two protomers A and B (“Qe-A” and “Qe-B”), respectively. Subsequent MD simulations showed that Qe is not stable within the binding site *h-2* (Fig. 3e). In a second attempt, Qe was docked into *h-2* using constrained docking protocol where previously docked Qi was assigned as templates, and Qe forced to share a maximum common structure with an RMSD deviation of <0.3 Å (Fig. 3d). The constraint docking score values are of −6.2 and −6.8 kcal mol^−1^ for Qe-A and Qe-B, respectively, and binding modes for Qe and Qi within the *h-2* site were identical. However, MD simulations confirmed that also, in this case, Qe, despite sharing largely overlapping binding modes with Qi, is not stable (Fig. 3e). These findings highlight the important role of sugar moiety in stabilizing Qi molecules within the *h-2* binding site.

### Relevance of the dimer disruptor site (*sc-2)* in *sc*Ire1p dimers

Having shown the strong interaction of Qi with *h-2*, we explored the possibility of the two compounds binding to the corresponding site in *sc*Ire1p using molecular docking and BPMD simulations. The findings show that the two molecules interact in very different modes in this site. Qi does not form stable interactions in the *sc-2*, equivalent to the dimer disruption site *h-2* in *h*IRE1, whereas Qe does bind but the interaction is much weaker than to the activation site *sc-1*. Full details of the dockings, simulations, and resulting data are available in the Supplementary Discussion, including Supplementary Fig. 3.

### Identification of additional binding sites in *h*IRE1

**Fig. 4:**
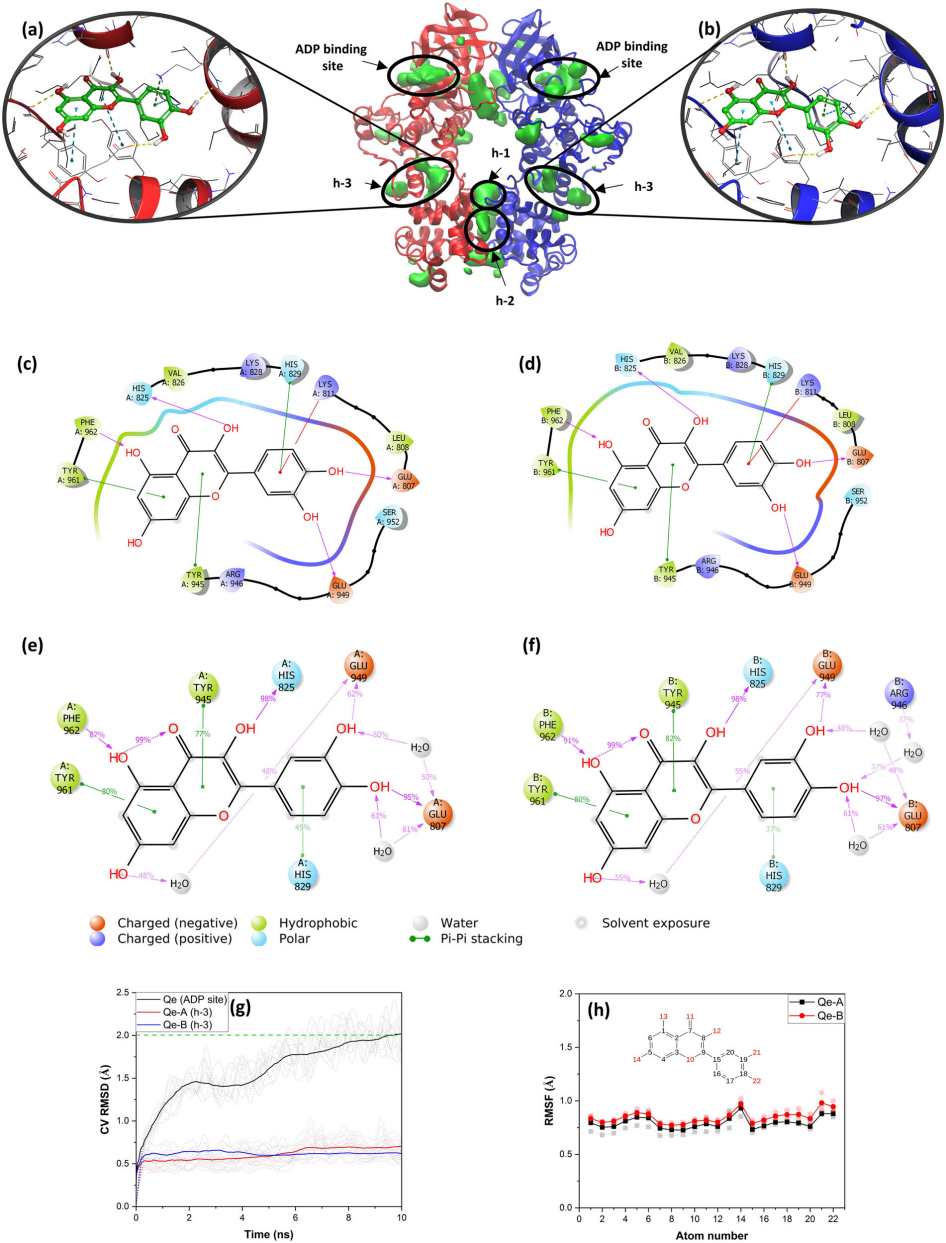
The binding poses of Qe in binding site *h-3* of the *h*IRE1 dimer. **a** Qe-A and **b** Qe-B docked to the *h-3* sites. 2D interaction diagram showing the interacting residues of **c** Qe-A and **d** Qe-B molecules after docking calculations. The green surfaces are the predicted binding sites (cavities) using FPocket depicted by a set of combined alpha spheres. 2D interaction diagram showing the interaction abundances (binding contributions) during the MD simulations for **e** Qe-A and **f** Qe-B molecules in site *h-3*. **g** The individual (dashed lines, 10 independent BPMD simulations) and corresponding average ligand RMSD curves (calculated over 10 independent BPMD simulations) for Qe molecules bound to site *h-3* and to the ADP binding site. **h** The individual (dashed lines, triplicates MD simulations) and the corresponding average RMSF values of Qe-A and Qe-B molecules bound to site *h-3*, during 3 × 300 ns MD simulations.

According to the results reported above, Qe, which is a potent activator of *sc*Ire1p, could bind to neither *h-1* nor *h-2-*binding sites. To explore if additional bindings sites are present in the *h*IRE1 dimer (PDB ID: 4YZC) that could accommodate the Qe molecule, the Fpocket tool (Supplementary Note 1) was applied to 900 structures from the 3 × 300 ns classical MD simulation trajectories (Fig. 4). Fpocket was able to identify binding sites *h-1*, *h-2* and the ADP binding site within the kinase domain. Additionally, it identified three more pockets. Among these, two were classified as transient due to their association with low isovalue clusters of grid points (lower than %25). In contrast, the third pocket (binding site *h*-3), was deemed permanent. To investigate the transient binding pockets, Qe and Qi molecules were docked using the corresponding MD snapshots that featured the highest number of alpha spheres. For the binding site *h*-3, Qe, and Qi molecules were docked using the *h*IRE1 dimer (PDB ID: 4YZC). The docking calculations resulted in no binding possibility to the transient sites but two binding sites where Qe and Qi preferentially bound: (a) the ADP binding site and (b) a new binding pocket located in the structural linker region between the kinase and RNase domains (site *h-3*; Fig. 1e).

### Qe and Qi bind with moderate strength to the ATP binding site

To explore if the inhibitory effect of Qe and Qi arise due to binding to the kinase pocket, extensive docking, BPMD, and MD simulations were performed, and results were compared with those of ADP. The data show that, although both Qe and Qi are able to bind to the ATP binding site, the interaction is weak, and will not compete with ADP. Full details of the calculations and findings for the ATP binding site are given in Supplementary Discussion, including Supplementary Fig. 4.

### Qe binds to a new allosteric binding site (*h-3*) in *h*IRE1 dimer

The new binding site, *h-3* (Fig. 1e) identified using Fpocket, is located in the structural linker region in between the kinase and RNase domains of *h*IRE1 (Fig. 4a, b). The grid box corresponding to the *h-3* binding site was generated based on the residues around the pocket, as outlined in the Methods section. Qe and Qi were subsequently docked into the site, which resulted in docking score values −6.87, −6.81, −5.12, and −5.26 kcal mol^−1^ for Qe-A, Qe-B, Qi-A, and Qi-B, respectively. A series of extensive MD and BPMD simulations were conducted to address the stability of Qe/Qi in this binding site. These showed that Qi is not stable in *h-3*, whereas for Qe the PoseScore values calculated from the BPMD simulations (0.64 and 0.62 Å for Qe-A, Qe-B, respectively; lower is better) indicate that this molecule binds significantly better in site *h-3* than in the ADP binding site (PoseScore 1.96 Å). Figure 4c, d show how Qe is predicted to interact with the residues in each chain, *i.e*., three hydrogen bonds with the backbone of resides His825, Glu807, and Phe962, one hydrogen bond with the sidechain of residue Glu949, three π-π stacking interactions with residues His829, Tyr945, and Tyr961, and one π-cation interaction with the sidechain of residue Lys811. Figure 4e, f confirms that these interactions are maintained during the 300 ns MD simulations. Additional analysis, i.e., time series of atomic interactions between Qe molecules and the surrounding residues and histogram of interactions per residue, were conducted using the geometrical criteria taken from^17^ and presented in Supplementary Figs. 5 and 6. Figure 4g illustrates the RMSD curves calculated from 10 independent BPMD simulations (dashed lines) along with the corresponding average curves (solid lines) for Qe-A and Qe-B in *h-3* compared to those of Qe bound to the ADP binding site. As seen, all independent RMSD curves for Qe molecules in the binding site *h-3* reached a plateau below 1 Å while for Qe bound to the ADP binding site the RMSD cures are considerably higher. Therefore, there is a significant difference in stability between the two binding sites, with clear preference for *h-3*. The heavy-atom RMSF values calculated from three independent 300 ns classical MD simulation (dashed lines) and the corresponding average values (solid lines) (Fig. 4h) are all below 1 Å for each atom of Qe molecules bound to*h-3*, indicating superior stability. The average free energies of Qe binding to *h-3* are ~−50 kcal mol^−1^ (Fig. 2d). As Fig. 3b shows, unlike what is noted for Qe in *sc-1*, there is only a small effect on the free energy of binding between the two protomers in the *h*IRE1 dimer when Qe binds to *h-3*.

### Qe and Qi do not bind to the allosteric site 3 in the *sc*Ire1p dimer

To generate the grid box for binding site *sc-3*, the corresponding residues for site *h-3* in *h*IRE1 were used in the *sc*Ire1p dimer, followed by flexible docking of the Qe and Qi molecules. The molecular docking calculations only resulted in few binding poses for both Qe and Qi, in all cases being different from those observed in *h*IRE1. MD and BPMD simulations showed that neither Qe nor Qi were stable enough to remain in the binding site during the simulation campaigns. The linker region site *3* is, hence, not a good binding site for either Qe or Qi in the *sc*Ire1p dimer. From the sequence alignment (Fig. 1f) we also note the low correlation between the two proteins at this site (33.3% sequence identity), strongly affecting the possible interactions.

### Comparative similarity analysis of binding sites between *h*IRE1 and *sc*Ire1p

A pairwise sequence alignment was conducted to compare the amino acid composition of each binding site in *h*IRE1 and *sc*Ire1p. The Multiple Sequence Viewer/Editor module of the Schrödinger program was employed to align the cytoplasmic part of *h*IRE1 (465 – 977) and *sc*Ire1p (556–1115) using FASTA files (*h*IRE1: O75460 and *sc*Ire1p: P32361) retrieved from the UniProt database (www.uniprot.org). The residues in the blue, green, and yellow dashed boxes are those associated with binding sites 1, 2, and 3, respectively (Fig. 1f). The binding site domains were identified as all residues within 7.0 Å distance from the ligand molecules i.e., Qe in *sc-1* for site 1, Qi in *h-2* for site 2, and Qi in *h-3* for site 3. Residue identity between *h*IRE1 and *sc*Ire1p is 15.8, 50.0, and 33.3% for site 1, site 2, and site 3, respectively (Fig. 1f). This large difference in local environment between the two proteins can be expected to greatly impact their binding capabilities.

### In vitro cleavage assays and MST measurements confirm Qe and Qi as *h*IRE1 inhibitors

**Fig. 5:**
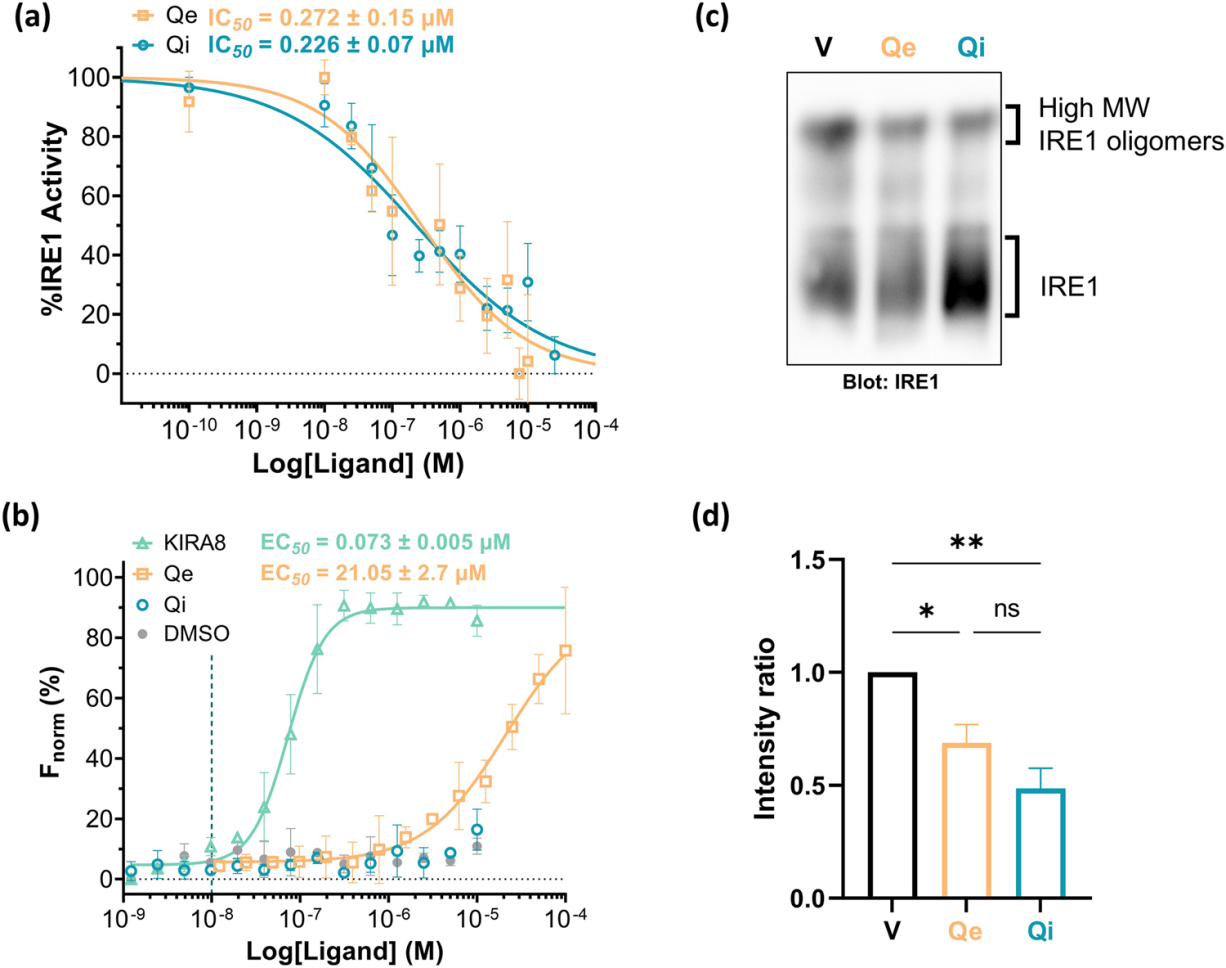
Effects of quercetin and quercitrin on IRE1 activity. **a** IRE1 RNase activity measured using in vitro cleavage assay in the presence of increasing concentrations of Qe (yellow) or Qi (cyan). The cleavage assay relied on the use of a fluorescence-quenched mini XBP1 RNA substrate probe (Cy3-CAUGUCCGCAGCGCAUG-BHQ3), which when cleaved by IRE1 emits fluorescence at 590 nm (cy5) wavelength. Symbols and error bars represent mean values (*n* = 3) ± SEM. **b** Displacement assays using microscale thermophoresis of the ADP-site bound Kinase Tracer 236 by KIRA8 (mint), a known IRE1 ADP-binding site ligand, Qe (yellow) or Qi (cyan). Symbols and error bars represent mean values ± SD (*n* = 3). **c** HEK293T cells overexpressing IRE1 were treated with vehicle (DMSO), 10 μM Qe, or 10 μM Qi for 2 hours. Detection of IRE1-containing complexes was achieved using Western blot following protein separation by native gel electrophoresis. **d** Quantification of **c**. Densitometry of high molecular weight IRE1 oligomers and IRE1 entities was determined using ImageJ, and ratios were determined and normalized to the vehicle condition. Symbols and error bars represent mean values ± SEM. ns: non-significant; *p values ≤ 0.05; **p values ≤ 0.01.

To validate and quantify the inhibitory effect of Qe on *h*IRE1, we measured IRE1 RNase activity in vitro as previously reported for Qi and other IRE1 inhibitors^15,18^. Dose-dependent assays resulted in the determination of an IC_50_ of 0.226 ± 0.07 µM for Qi, a value identical to the IC_50_ found in our previous study^15^, and of 0.272 ± 0.15 µM for Qe, thus confirming Qe as a *h*IRE1 inhibitor although it is an activator in *sc*Ire1p (Fig. 5a). To validate the allosteric mode of action of Qe and Qi, we developed a displacement assay using a known kinase-site bound ligand bearing a fluorophore, the Kinase Tracer 236 (KT236), thereby allowing us to determine by microscale thermophoresis (MST) the effective concentration (*EC*_50_) of ligand needed to compete for this binding site. As a positive control of binding to the kinase pocket, we used KIRA8^19^, a very potent IRE1 kinase inhibitor able to strongly compete with KT236, as shown by the low EC_50_ of 0.073 ± 0.005 µM measured (Fig. 5b). No displacement could be observed for Qi up to 10 µM, whereas titration of Qe resulted in signal change starting at the micromolar range. Titration at a higher concentration range going up to 100 µM allowed us to determine an EC_50_ of 21.05 ± 2.7 µM for Qe (Fig. 5b). These results demonstrate that Qe and Qi exert their inhibitory action on IRE1 RNase through an allosteric mode of action as they do not bind with high affinity to the kinase pocket, and they also cannot bind covalently with Lys907 of the catalytic RNase site as they do not possess the required hydroxy-aryl aldehyde moiety. To study further their effect on *h*IRE1, we tested if those molecules were able to alter the formation of IRE1 complexes. To this end, HEK293T cells overexpressing IRE1 were treated with vehicle (DMSO), Qe, or Qi for 2 hours followed by protein separation in native gel electrophoresis and Western blot with anti-IRE1 antibodies (Fig. 5c, d, Supplementary Fig. 7, and Supplementary Data 1). The amount of high molecular weight *h*IRE1 oligomers is reduced upon Qe and Qi treatments, while at the same time an increase of monomeric species is seen, especially accentuated when the cells are treated with Qi. Taken together, these experiments show that both, Qe and Qi are able to inhibit IRE1 RNase activity (Fig. 5a) through an allosteric mode of action (Fig. 5b), and that Qi and, to a lesser extent Qe affect IRE1 dimerization/oligomerization (Fig. 5c).

## Conclusions

We herein provide a detailed rationale for the observed discrepancy of Qe-mediated activation of *sc*Ire1p dimers^16^ and Qi (and Qe, current study)-mediated inhibition of *h*IRE1 RNase activity^15^. In addition to this, the effects of Qe on ER stress in mammalian cells and on IRE1 activity are currently controversial with articles describing either inhibiting or activating effects of this molecule. In our study, three allosteric binding sites were identified and explored in both *sc*Ire1p and *h*IRE1 dimers. These sites included Site 1 located in the region of the IRE1 dimer where Qe co-crystallized with *sc*Ire1p^16^ (*sc-1* and *h-1*), Site 2 in the dimer disruption binding site observed for Qi in *h*IRE1 dimers^15^ (*sc-2* and *h-2*), and Site 3 in the linker region between the kinase and RNase domains (*sc-3* and *h-3*).

In *sc*Ire1p dimer, binding site *sc-3* provided stable association neither for Qe nor for Qi, and site *sc-2* exhibited much less stable binding than the activator site *sc-1*, for both ligands. Through extensive MD simulations, we observed that both Qe and Qi binding to *sc-1* leads to a significantly enhanced binding affinity of the dimer, thus explaining the increased RNase activity. While there is no marked difference in distance between the two RNase domains upon Qe or Qi binding to site *sc-1*, the fluctuations (‘breathing motions’) of the center of mass distance become much reduced, in agreement with the free energy profiles of the two protomers showing a deep minimum as compared to the apo form. Binding to *sc-1* hence stabilizes the dimer and ‘locks’ the RNase pocket of *sc*Ire1p in its catalytically active conformation, leading to activation thereof.

In *h*IRE1 dimers, the situation is markedly different, which can be traced back to the very low sequence identities in the two sites between *h*IRE1 and *sc*Ire1p. No binding could be observed in the corresponding activation site *h-1* of the *h*IRE1 dimer. Instead, Qi was found to bind very strongly in the dimer disruptor site, *h-2*, previously identified^15^, and Qe preferentially to the novel allosteric site *h-3*. This is consistent with the fact that both Qe and Qi inhibit *h*IRE1 RNase activity, with IC_50_ values in the sub-micromolar range. MST measurements of displacement of the ADP-site bound Kinase Tracer 236 by KIRA8, Qe, and Qi, clearly showed that neither Qi nor Qe are kinase inhibitors, which is also in agreement with the computed free energies of binding. The mechanisms by which Qi works might involve the disruption of *h*IRE1 oligomers to increase monomeric, inactive *h*IRE1. The proposed binding by Qe at the allosteric linker site, in all likelihood, affects the catalytic activity of the RNase site (as seen by the IC_50_ values) but does not directly impose on dimer formation. This inhibitory activity of Qe towards *h*IRE1 RNase could be consistent with several physiological effects of Qe, for instance, regarding its anti-inflammatory properties^20–23^, provided that IRE1 signaling is pro-inflammatory^24,25^. Although Qe has been associated with numerous health benefits related to inflammation and immune function^26^, Boots et al.^27^ introduced the concept of the “quercetin paradox” in their study using rat lung epithelial (RLE) cells. The quercetin paradox suggests that while quercetin offers protection, its metabolism leads to the formation of potentially toxic byproducts. These toxic metabolites of Qe can lead to a decrease in glutathione levels, an increase in lactate dehydrogenase leakage, and an elevation in cytosolic free calcium concentration.

Our study provides a clear explanation of the observed activation of *sc*Ire1p by Qe, and on the mechanisms of *h*IRE1 RNase inhibition by Qe and Qi. Both dimer disruption (through site *h-2*) and inhibition of *h*IRE1 RNase activity (through allosteric site *h-3*) represent new means of modulating IRE1 activity, with very promising implications for the future development of novel therapeutics since by targeting these sites the entire issues of selectivity of ATP binding kinase pockets and the covalent inhibition of the RNase pocket could be avoided. However, further experimental substantiation (like mutagenesis analysis) may be required to fully support the in silico results regarding the actual existence of the binding site-3 in *h*IRE1 and the associated inhibitory effect of Qe.

## Methods

### Definition of binding sites

Six binding sites in the RNase domain of *sc*Ire1p and *h*IRE1 dimers have been extensively investigated, three in each of the IRE1 systems (Fig. 1c–e). Binding site 1 is the activating binding region of the co-crystalized Qe molecules in *sc*Ire1p back-to-back dimer (pdb id: 3LJ0^16^, Fig. 1c). Binding site 2 is the binding site of Qi in *h*IRE1 (pdb id: 4YZC, Fig. 1d) previously identified by Amarasinghe et al.^15^. This binding site is located in the interface between two RNase domains where Qi molecules bind and disrupt the dimer and eventually block the RNase activity of *h*IRE1. A new potential allosteric binding pocket in *h*IRE1 (site 3) is identified herein using the Fpocket tool^28,29^, and was found to perfectly accommodate the Qe molecule (Fig. 1e). This site is in the structural linker region located in between the kinase and RNase subdomains. The three sites will in the following be labelled *h-1, h-2, h-3* in *h*IRE1, and *sc-1, sc-2, sc-3* in *sc*Ire1p, respectively. A brief explanation of how the Fpocket tool works can be found in the supporting information.

### Protein and ligand preparation

The back-to-back dimer structures of the *sc*Ire1p (pdb id: 3LJ0) and *h*IRE1 (pdb id: 4YZC) were retrieved from the Protein Data Bank (PDB). The crystal structures were prepared using the protein preparation wizard in the Schrödinger 2020-2 program package (https://www.schrodinger.com). Hydrogen atoms were incorporated, and missing side chain atoms and missing loops were added using Prime^30,31^. After fixing structural defects, water molecules were removed from the system, and the protonation states of ionizable residues were determined at pH = 7.0. The prepared structure of IRE1 was further refined using the OPLS4 force field^32^ in a restrained minimization procedure with an RMSD threshold of 0.3 Å for all heavy atoms. The structures of the Qe and Qi molecules were prepared using the LigPrep module (LigPrep, Schrödinger, LLC, New York, NY, 2020.) in the Schrödinger 2020-2 package and Epik tool at pH = 7.0 using OPLS4 force field. The lowest state penalty structures were chosen for further calculations.

### Molecular docking

The Glide tool^33^ in the Schrödinger 2020-2 package was employed to perform the molecular docking calculations. The docking procedure was first validated by reproducing the binding pose of Qe co-crystalized in the *sc*Ire1p back-to-back dimer (pdb id: 3LJ0). In this benchmarking attempt, two grid boxes were generated with each of the Qe molecules (Qe-A and Qe-B) at the centers of the boxes. Each Qe molecule (either Qe-A or Qe-B) was docked in the presence of the second, already bound, counterpart. The superposed co-crystalized and docked Qe molecules are shown in the RNase dimer interface region of the *sc*Ire1p back-to-back dimer (Supplementary Fig. 1). The very low RMSD values (0.26 and 0.23 Å for Qe-A and Qe-B, respectively, Table S1) indicate a successful docking protocol. Both the co-crystalized and the docked Qe-A ligand interacts with residue A:Phe1112 (π-π stacking), A:Ser984 (hydrogen bonding), and B:Glu988 (hydrogen bonding) and Qe-B correspondingly interacts with residue B:Phe1112 (π-π stacking), B:Ser984 (hydrogen bonding) and A:Glu988 (hydrogen bonding).

The receptor grids for binding site *sc-1* in *sc*Ire1p, which were generated using the co-crystalized Qe molecules as the center of the boxes, were transferred accordingly to the corresponding area of binding site *h-1* in *h*IRE1 for the “cross-docking”. The receptor grid boxes for binding site *h-2* (*h*IRE1) were generated using the Qi molecules^15^ as the center of the boxes and subsequently transferred to the corresponding areas of binding site *sc-2* in *sc*Ire1p. Flexible docking of the prepared ligands into the receptor grid boxes was performed using extra precision (XP) GlideScore^33,34^ and OPLS4 force field. To soften the potential for the non-bonded parts of the ligands, the van der Waals radii of ligand atoms with partial atomic charges less than 0.15 were scaled by a factor of 0.80. The other docking parameters were set to the default values.

Induced fit docking^35,36^ was performed using the Schrödinger package for the molecular docking calculations in sites *h-3* and *sc-3*. The receptor grid box was generated based on the residues around the binding pocket in *h*IRE1 (Tyr945, Lys811, His825, and Glu949) identified using the Fpocket tool. The corresponding residues in *sc*Ire1p (Tyr1096, Gln959, Lys973, and Lys1100) were identified after sequence alignment and used for the grid box generation in site *sc-3*. All residues within 5.0 Å radius from the ligands were considered flexible, and the sidechains were optimized using Prime. The rest of the induced fit docking setting options were the same as for regular docking.

### Molecular dynamics simulation

Molecular dynamics (MD) simulations were performed using the Desmond MD engine^37^, an explicit-solvent molecular dynamics program implemented in the Schrödinger 2020-2 package. The TIP3P water model^38^ was applied for water molecules in a cubic box with 10 Å buffer distance to avoid unphysical interactions with the periodic images. Biological salt concentration (0.15 M) was considered, and counter ions (Na^+^/Cl^−^) were added to neutralize the system charge. The default Desmond protocol was applied for the minimization and relaxation steps prior to the start of the simulations. Periodic boundary conditions and OPLS4 force field were applied in NPT ensemble MD simulations, in which Nose-Hoover temperature coupling^39^ and Martyna–Tobias–Klein barostat^40^ were employed to keep the temperature and pressure kept constant at 300 K and 1 atm pressure, respectively. Following the relaxation steps, the MD simulations were run for 300 ns with a trajectory sampling frequency of 300 ps in the production phase. RESPA multi-timestep integrators^41^ (with bonded-, near-, and far-timesteps of 2.0, 2.0, and 6.0 fs, respectively) were employed to integrate the equations of motion in phase space. All covalent bonds connecting hydrogen atoms to the heavy atoms were kept fixed using the SHAKE algorithm^42^. A fixed cutoff distance of 10.0 Å was used for the short-range van der Waals and electrostatic interactions, while the smooth particle mesh Ewald (PME) algorithm^43^ was applied for the long-range electrostatic forces. The MD simulations were done in triplicate with different initial atomic velocity distributions; all data presented are based on the averages over all three trajectories.

### Free energy of binding calculations

The ligand-free energy of binding in each protein/ligand complex was calculated using the molecular mechanics generalized Born surface area (MM-GBSA)^44^ technique implemented in the Schrödinger 2020-2 package. To enhance the accuracy and reliability of the calculated free energy values, the MM-GBSA algorithm was applied on the triplicate MD trajectories at every 3 ns, giving ~100 snapshots per MD trajectory and a total of ~300 snapshots for each system. From this data, the average value was calculated. The same force field and cutoff distances were used as applied in the MD simulations.

### Binding pose metadynamics

Binding pose meta-dynamics (BPMD)^45^ is an automated enhanced sampling technique that imposes a biasing potential to force the ligand to move in and around its binding site. Ligand poses that are unstable under the bias of the metadynamics simulation are expected to be infrequently occupied in the energy landscape, thus making minimal contributions to the binding affinity. In this study, BPMD simulations were carried out using the Desmond MD engine with 10 independent metadynamics simulations (10 ns each) performed for each system. BPMD generates a “PoseScore”, a metric that indicates the average root-mean-square deviation (RMSD) from the starting pose and can be considered as a measure of the ligand stability in the binding site. A ligand with PoseScore <2 Å is regarded as being stably bound to the site. The CV in the BPMD simulation is the RMSD of all heavy atoms in the ligand. The height and width of Gaussian kernels, hill deposition interval, and biasing factor (*k*_b_ΔT) were set to 0.05 kcal mol^−1^, 0.02 Å, 1 ps, and 4.0 kcal mol^−1^, respectively. The remaining BPMD settings were the same as in regular MD simulations.

### Well-tempered metadynamics

A series of well-tempered metadynamics (WT-MetaD) simulations were performed using the Desmond MD engine^37^ to evaluate the effect of the co-crystalized Qe molecules bound to site *sc-1*, on the dynamic and interaction profile of the two RNase domains in the *sc*Ire1p dimer. WT-MetaD is a computational technique used in molecular dynamics simulations to enhance the conformational sampling of rare events in complex systems^46^. In WT-MetaD, a history-dependent bias potential is added to the system, and the height and shape of this potential are adaptively adjusted during the simulation to encourage the exploration of different conformational states. The “well-tempered” aspect of this method refers to the adaptive nature of the bias potential, which gradually decreases over time to avoid excessive perturbation of the system. This helps in maintaining a balance between the exploration of the free energy landscape and the accuracy of the resulting data^46^.

The WT-MetaD simulations were conducted in duplicate for two systems: the *sc*Ire1p dimer with Qe molecules bound into the site *sc-1*, and the apo-*sc*Ire1p dimer. The CV used in the WT-MetaD simulations is the distance between the center of mass (*R*_COM_) of the RNase domains in each monomer (residues 983–1115). The width of the Gaussian kernels has been set to ¼ of the CV fluctuations calculated from unbiased MD simulations. The height of the hills, deposition interval, and biasing factor (*k*_b_ΔT) were set to 0.1 kcal mol^−1^, 1 ps, and 10 kcal mol^−1^, respectively. The rest of the parameter settings were the same as in the regular MD simulation.

### IRE1-mediated in vitro RNase assay

The in vitro RNase assays were performed as previously described^47^. Briefly, the molecules were diluted in a minimal volume of DMSO and subsequently re-diluted in reaction buffer (20 mM HEPES pH 7.5; 1 mM MgOAc; 50 mM KOAc). Maximum volume of DMSO per reaction never exceeded 1%. The reaction volume was 25 μL. Recombinant IRE1 (0.6 μg/reaction, aa 465–977, His & GST Tag, purchased from SinoBiological, Cat: 11905-H20B) was incubated at room temperature for 10 minutes with varying concentrations (0–100 μM) of inhibitor and reaction buffer. The assay relied on the use of a fluorescence-quenched mini XBP1 RNA substrate probe (Cy3-CAUGUCCGCAGCGCAUG-BHQ3, Eurogentec), which, when cleaved by IRE1 emits fluorescence at 590 nm (cy5) wavelength^47^. Equal volumes of a mixture of reaction buffer, 20 mM ATP, 2 mM DTT, and 1 μg of fluorescent probe were added to each sample in 96 well plates flat bottom, black polystyrene, matrix active group High Bind (“Corning”), and fluorescence was read every minute for 25 minutes, at 37 °C, using a Tecan 200 plate reader.

### MST measurements

MST measurements were performed on a Nanotemper Monolith NT.115 at 70% LED power (red excitation) and 40% MST power using Monolith NT.115 Premium Capillaries (MO-K025). Sample preparations were done in black, nonbinding surface, round-bottom 384 microwell plates (Corning #4514), and plates were incubated at room temperature and protected from light for 1 h before measurements. All samples were prepared using recombinant IRE1 protein (aa 465-977, His & GST Tag, purchased from SinoBiological, Cat: 11905-H20B) and Kinase Tracer 236 (KT236, ThermoFisher^TM^, PV5592) in Kinase Buffer A (InvitrogenTM, PV6135) diluted to 1× prior to use (HEPES 50 mM (pH 7,5), MgCl2 10 mM, EGTA 1 mM and Brij-35 0,01%) with a final volume per well of 10 µL. *Protein titrations*: 10 nM of the fluorescently labeled KT236 was titrated with varying concentrations of IRE1 protein (highest concentration = 1745 µM, dilution factor 1:1, 14 data points) in order to determine the *K*_d_-value and select appropriate protein and tracer concentrations for the subsequent competition experiments (Supplementary Data 3). Analysis of the normalized fluorescence time traces was performed using the default “thermophoresis + t-jump” setting in the analysis software, and the *F*_norm_ (**‰**) values were plotted against the logarithmic protein concentration. The data (*n* = 2) were fitted with the “[Inhibitor] vs. response–variable slope (four parameters)” equation from GraphPad Prism for Windows (GraphPad Software Inc., CA, USA). *Compounds titrations*: dilution series of the inhibitors dissolved in kinase buffer A (prepared from 10 mM stock solution of inhibitors in DMSO; final concentration of DMSO < 1%, dilution factor 1:1, 14 data points) were made with fixed concentration of labeled KT236 (10 nM) and protein (180 nM) (Supplementary Data 2). In addition to the 14 data points of the ligand dilution, two data points were reserved for control conditions, namely KT236 (10 nM) + IRE1 (180 nM) and KT236 alone (10 nM), representing the lower (KT236 fully bound to IRE1) and upper plateau (KT236 fully unbound), respectively. As an independent control, DMSO at a final concentration of 1% v/v was also measured. The data (*n* = 3) were fitted with the “[Inhibitor] vs. response – Variable slope (four parameters)” equation from GraphPad Prism for Windows (GraphPad Software Inc., CA, USA).

### Western blotting

Cells grown on six-well plates were washed with PBS and lysed with NativePAGE™ sample preparation kit to extract proteins according to the manufacturer’s instructions (Invitrogen™ Ref. BN2008). Protein extracts were resolved with NativePAGE™ 3–12%, Bis-Tris, 1,0 mm, minigels (Invitrogen™ Ref. BN1001BOX) for 4 hours at 4 °C and transferred to PVDF membranes for 16 hours, 30 V at 4 °C using a Mini Trans-Blot^®^ (BioRad® Transfer System #1703930). Proteins were fixed in the membrane with 8% acetic acid. IRE1 was stained using anti‐IRE1 antibody (Anti-human; rabbit polyclonal; Cell signaling (14C10) mAb #3294) for 16 hours at 4 °C, washed with PBST, and incubated for 1 hour with goat anti‐rabbit at room temperature (Invitrogen, Carlsbad, CA, USA) prior revelation using chemiluminescence (ECL RevelBlOt® Intense, Ozyme). Protein levels were quantified from the gels by Fiji software. The ROIs were manually captured, and the bands were quantified according to their intensities.

### Statistical analyses

Data are presented as mean ± SEM or ±SD as indicated in each figure. Statistical significance (*P* < 0.05) was determined using unpaired *t*‐tests or ANOVA as appropriate, and performed using GraphPad Prism software (GraphPad Software, San Diego, CA, USA). Curve extrapolations were performed using curve fit hypotheses by GraphPad Prism software (GraphPad Software, San Diego, CA, USA).

### Reporting summary

Further information on research design is available in the Nature Portfolio Reporting Summary linked to this article.

### Supplementary information

Supplementary information

Description of Additional Supplementary Files

Supplementary Data 1

Supplementary Data 2

Supplementary Data 3

Reporting Summary

## Data Availability

Supplementary data with setup descriptions for docking procedure, benchmarking, and study of Qe and Qi binding to *sc*Ire1p *sc-1* and *sc-2* sites, and *h*IRE1 *h-2* and *ATP* binding sites, including Supplementary Figs. 1–6 and Supplementary Table 1, as well as western blot gels in Supplementary Fig. 7, are available in Supplementary information. Unprocessed gels for Supplementary Fig. 7 are provided in Supplementary Data 1. The output files for MST experimental measurements and K_d_ determination are provided in Supplementary Data 2 and Supplementary Data 3, respectively. Docked structures, MD trajectories, BPMD simulations, and video of the RNase opening/closing cycles in apo and Qi-bound *sc*Ire1p are provided freely at zenodo.org with 10.5281/zenodo.7937826.
