# Supplementary material for: Different binding modalities of quercetin to inositol-requiring enzyme 1 of S. cerevisiae and human lead to opposite regulation
Source: Commun Chem. 2024 Jan 5;7:6. doi: 10.1038/s42004-023-01092-0 (PMC10767055; doi:10.1038/s42004-023-01092-0)
Supplement: Supplementary file 1 — Supplementary information [file 42004_2023_1092_MOESM1_ESM.pdf]

SUPPORTING MATERIAL

Different binding modalities of Quercetin to Inositol-requiring enzyme 1 of *S. cerevisiae* and Human lead to opposite regulation.

S. Jalil Mahdizadeh[1], Johan Grandén[1], Diana Pelizzari-Raymundo[2,3], Xavier Guillory[2,3,4], Antonio Carlesso[1,5], Eric Chevet[2,3,*], Leif A. Eriksson[1,*]

[1]Department of Chemistry and Molecular Biology, University of Gothenburg, 405 30 Göteborg, Sweden.

[2]INSERM U1242, Université de Rennes, Rennes, France.

[3]Centre de Lutte contre le Cancer Eugène Marquis, Rennes, France.

[4]Univ Rennes, CNRS, ISCR – UMR 6226, F-35000 Rennes, France.

[5]Department of Pharmacology, Sahlgrenska Academy, University of Gothenburg, SE-405 31 Gothenburg, Sweden

Corresponding authors

Leif A. Eriksson: leif.eriksson@chem.gu.se

Eric Chevet: eric.chevet@inserm.fr

Supplementary Discussion

Binding Site *sc-1* of *S. cerevisiae* Ire1p dimers enhance dimer stability

The Qe molecules were docked to the *sc-1* grid box with and without the counterpart present in the adjacent protomer (A/B). The results showed that Qe can bind to the same binding site with the same binding pose even in the absence of the other Qe molecule (Supplementary Figures 1a, 1b). The docking scores and RMSD values (compared to the co-crystalized ligand) are presented in Table S1. The docking score value is 3-4 kcal mol^{-1} lower (*i.e.* stronger interaction) in the complex with stoichiometry of 2:2, which clearly indicates that the two ligands stabilize each other. This was further confirmed by binding pose metadynamics simulations.

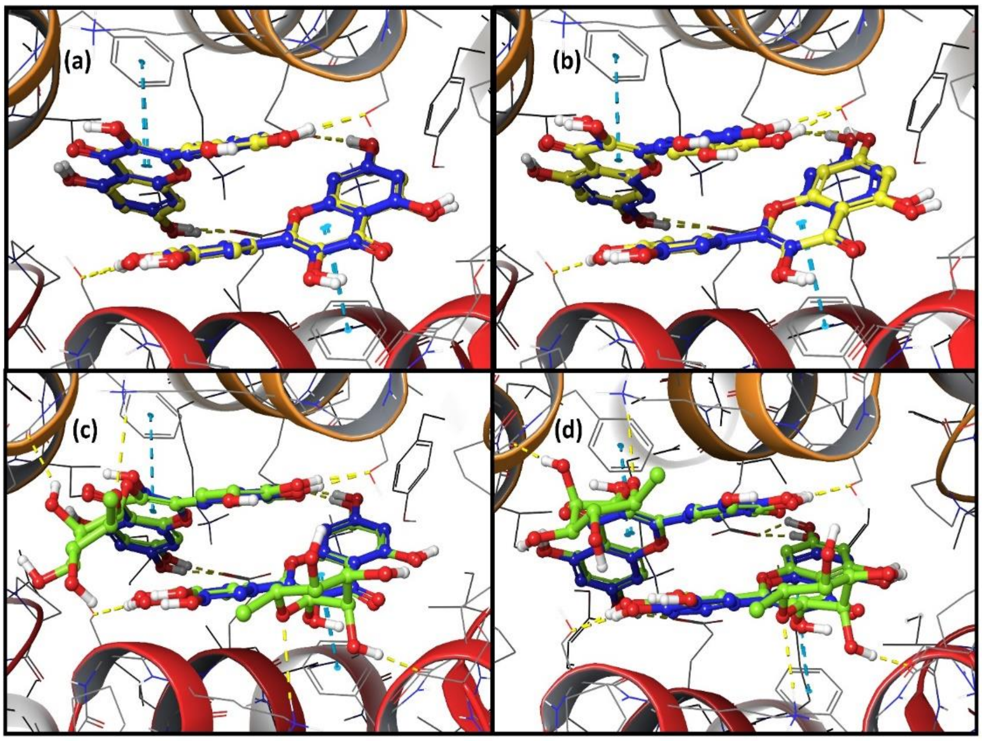

Supplementary Figure 1. Qe molecules docked to the *sc-1* site (yellow) in the **(a)** presence and **(b)** absence of the counterpart molecule, superposed on the co-crystalized ligands (blue). Qi docked to the *sc-1* site (green) in the **(c)** presence and **(d)** absence of the counterpart molecule, superposed on the co-crystalized Qe ligands (blue). Chains A and B of the *sc*IRE1 dimer are shown in orange and red, respectively.

Qi was also docked on the *sc-1* site. Qi is able to bind to the same pocket as Qe, with RMSD values of 0.30 and 0.31 Å for Qi-A and Qi-B, respectively, compared to the corresponding atoms in the Qe molecules (Supplementary Figures 1c, 1d and Supplementary Table 1). Qi interacts with the same residues as Qe, plus two additional hydrogen bond interactions with

Lys985 and Glu1111 residues through the sugar moieties. Because of these additional interactions, the docking score values of Qi is ~2 kcal/mol lower than that of Qe (Supplementary Table 1). As for Qe, the docking ability of Qi on the *sc-1* site was also investigated in the absence of the counterpart molecule to evaluate the possibility of complex formation with a 2:2 stoichiometry. Docking score results show that, similar to Qe, the 2:2 stoichiometry is favored for Qi bound to the *sc-1* site (Supplementary Table 1).

Ligand	Counterpart present	Docking Score (kcal mol^{-1})	RMSD (Å)[a]
**Qe-A**	**Yes**	**-9.3**	**0.26**
**Qe-B**	**Yes**	**-9.9**	**0.23**
**Qe-A**	**No**	**-6.3**	**0.43**
**Qe-B**	**No**	**-6.2**	**0.39**
**Qi-A**	**Yes**	**-11.6**	**0.30**
**Qi-B**	**Yes**	**-11.4**	**0.31**
**Qi-A**	**No**	**-8.3**	**0.52**
**Qi-B**	**No**	**-8.1**	**0.56**

[a]RMSD relative to Qe atoms in the crystal structure.

Supplementary Table 1. Docking scores and RMSD values of Qe and Qi molecules docked to binding *sc-1* in presence or absence of the counterpart molecule.

To investigate the stability and dynamics of Qe or Qi bound to the *sc-1* site, three independent MD simulations (300 ns in each run) were performed. The abundance of each interaction during the MD simulations was determined for both Qe-A and Qe-B, respectively (Supplementary Figures 2a, 2b). The main interacting residues are Phe1112, Ser984, Glu988, and Tyr1059, which are the same as those observed in the molecular docking calculations (Figure S1). The average RMSD and RMSF graphs for Qe are illustrated in Supplementary Figures 2c, 2d, respectively. The low average RMSD and RMSF values show that Qe is highly stable in the binding site. The abundance of the main interactions for Qi-A and Qi-B during the MD simulation are shown in Supplementary Figures 2f, 2g, respectively. Most of the interactions observed after molecular docking calculations were maintained during the MD campaign. The average ligand RMSD values for Qi (Supplementary Figure 2h) are low, indicating their stability in the binding site. The average RMSF values for Qi (Supplementary Figure 2i) are higher than those found for Qe, particularly regarding the sugar moiety (atoms 23-31). However, they are low enough (< 2 Å) to be considered as stable ligands.

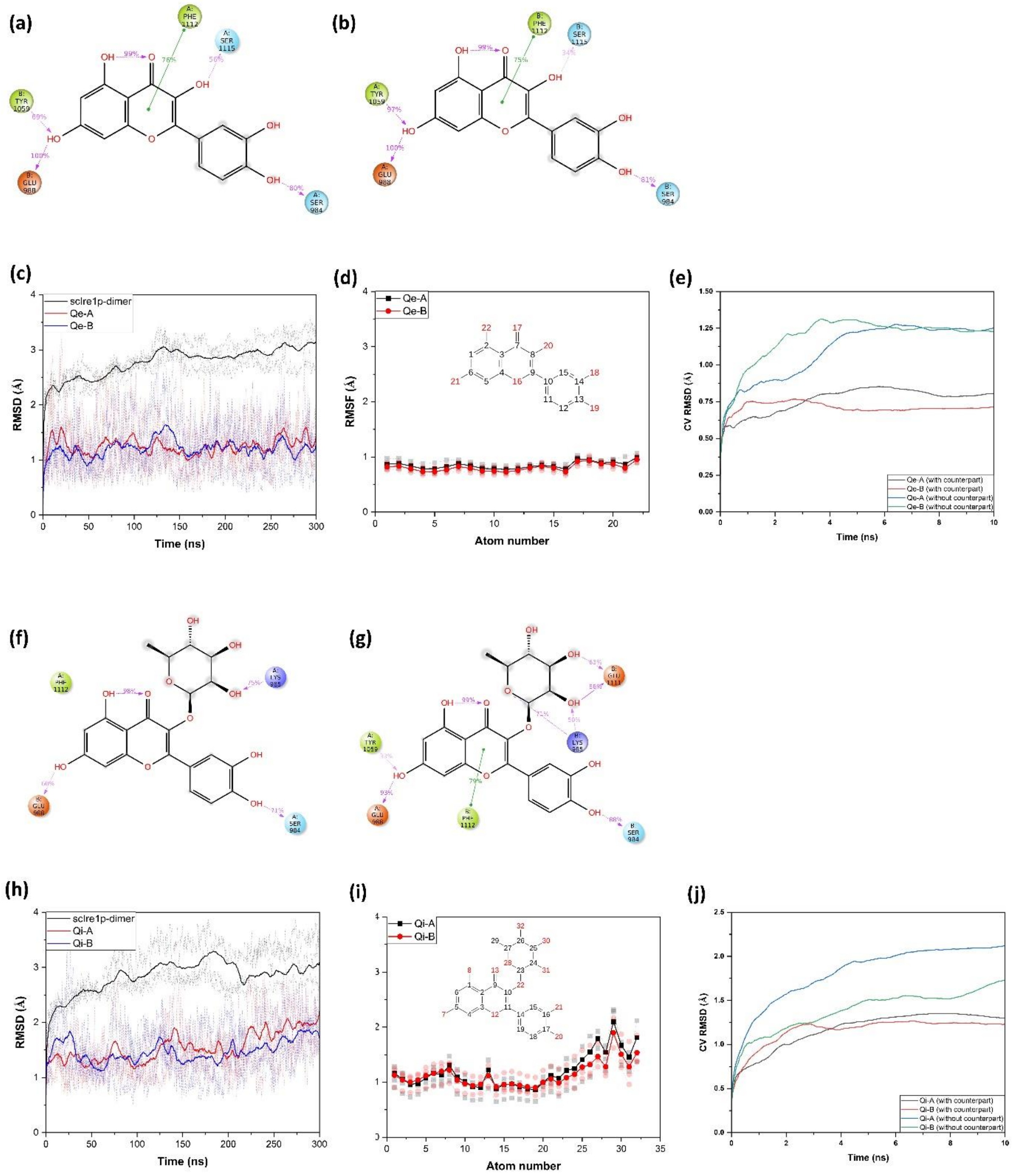

Supplementary Figure 2. Interactions, RMSD and RMSF data for Qe and Qi during MD simulations in site *sc-1*. 2D interaction diagram showing the interaction abundance during the MD simulation for **(a)** Qe-A and **(b)** Qe-B. The individual (dashed lines, triplicates 300 ns MD simulations) and the corresponding average **(c)** RMSD and **(d)** RMSF graphs for Qe molecules bound to site *sc-1*. **(e)** BPMD of Qe bound to the site *sc*-1 of protomer A or B, in absence and presence of counterpart. Interaction abundance during the MD simulation for **(f)** Qi-A and **(g)** Qi-B.The individual (dashed lines, triplicates 300 ns MD simulations) and the corresponding

average **(h)** RMSD and **(i)** RMSF graphs for Qi molecules bound to site *sc-1*. **(j)** BPMD of Qi bound to the site *sc*-1 of protomer A or B, in absence and presence of counterpart.

The average predicted free energy of binding values <ΔG_{Bind}> of Qe and Qi bound to the *sc-1* site were calculated from the triplicate MD simulations as described in the Methods section of the main text. The average free energies of binding for Qe-A and Qe-B are -61.0 and -61.2 kcal mol^{-1}, respectively, and the corresponding values for Qi-A and Qi-B are -64.1 and -66.1 kcal mol^{-1}, respectively. The higher free energies of binding for Qi are in agreement with the docking score values and is related to the formation of extra interactions caused by the presence of the sugar moiety in Qi (Supplementary Figures 2f, g).

Binding pose metadynamics simulations (BPMD) is a robust approach to distinguish stable (PoseScore < 2 Å) from unstable (PoseScore > 2 Å) ligand binding poses into a binding pocket [1, 2]. The average RMSD curves (over 10 independent well-tempered metadynamics simulation trials) was determined for Qe in the *sc-1* binding site in the presence or not of the counterpart molecule present (Supplementary Figure 2e). BPMD resulted in lower RMSD values for Qe in the presence of the counterpart ligand (PoseScore of 0.79 and 0.71 Å for Qe-A and Qe-B, respectively), compared to those without the counterpart ligand (PoseScore of 1.24 and 1.23 Å for Qe-A and Qe-B, respectively). Qi also attained lower RMSD values with the counterpart ligand (PoseScore of 1.32 and 1.23 Å for Qi-A and Qi-B, respectively; Supplementary Figure 2j), compared to those without (PoseScore of 2.10 and 1.62 Å for Qi-A and Qi-B, respectively). These results along with the molecular docking calculations clearly indicate that the two Qe or Qi molecules stabilize each other and confirm that complex formation with a 2:2 stoichiometry is more favorable than 2:1, in agreement with the crystal structure of the *sc*IRE1-Qe complex (pdb id: 3LJ0; [3]).

Relevance of the dimer disruptor site (*sc-2)* in *sc*Ire1p dimers.

The ability of Qe and Qi to disrupt dimer formation in *S. cerevisiae* Ire1p by binding to the region corresponding to the *h-2* site (i.e., site *sc-2*) was investigated using molecular docking calculations and BPMD simulations. To generate a proper grid box for binding site *sc-2* the structures of *h*IRE1 (4YZC) and *sc*Ire1p (3LJ0) back-to-back dimers were aligned, superimposed, and Qi was transferred from *h*IRE1 (*h-2* binding site) to *sc*Ire1p. The nearest residues were identified for the grid box generation (residues B:Lys992, B:Asp995, B:Gln1107, A:Tyr1059, A:His1060, and A:Phe1062 to generate the grid box for Qe-A and Qi-A, and conversely for the grid box for Qe-B and Qi-B). The Qe and Qi molecules were then

flexibly docked into binding site *sc-2*. The binding poses along with the interacting residues are shown in Supplementary Figures 3a-3f. The docking score values are -8.9 and -7.6 kcal mol^{-1} for Qe-A and Qe-B, respectively, and corresponding values -6.7 and -6.5 kcal mol^{-1} for Qi-A and Qi-B, respectively. As Supplementary Figures 3a, 3b shows, Qe-A interacts with residues A:Tyr1059 and B:Gln1107 while Qe-B interacts with A:Gln1107 and B:Tyr1059. These are among the residues that were defined to generate the grid boxes. For the Qi molecules, the situation is different. Qi-A interacts with residues A:Arg1056 and B:Glu988, while Qi-B interacts with residues A:Arg1056 and B:Asp1064 (Supplementary Figures 3c, d). These residues are different from those that were defined to generate the grid box. Unexpectedly, the Qe and Qi molecules hence bind with different binding poses and stabilities, in contrast to the findings for binding sites *sc-1* and *h-2* (Supplementary Figures 3e, 3f). The Qe molecules in binding site *sc-2* have ~1.5 kcal mol^{-1} worse docking score than in site *sc-1* and also a ~20 kcal mol^{-1} weaker free energy of binding, thus making binding site *sc-1* a more favored pocket in the yeast system. A series of BPMD simulations were conducted to evaluate the stability of Qe and Qi within the *sc-2* binding site and to compare the data with those for the sc-*1* site (Supplementary Figure 3g). Qi is not stable within the *sc-2* binding site (PoseScore: 2.53 and 2.51 for Qi-A and Qi-B, respectively), while it is stable in the *sc-1* site (PoseScore: 1.33 and 1.24 for Qi-A and Qi-B, respectively). Qe, on the other hand, is relatively stable also in the *sc-2* site (PoseScore: 1.78 and 1.31 for Qe-A and Qe-B, respectively). However, the stability is significantly higher in the *sc-1* binding site (PoseScore: 0.79 and 0.71 for Qe-A and Qe-B, respectively), in agreement with the crystallographic data.

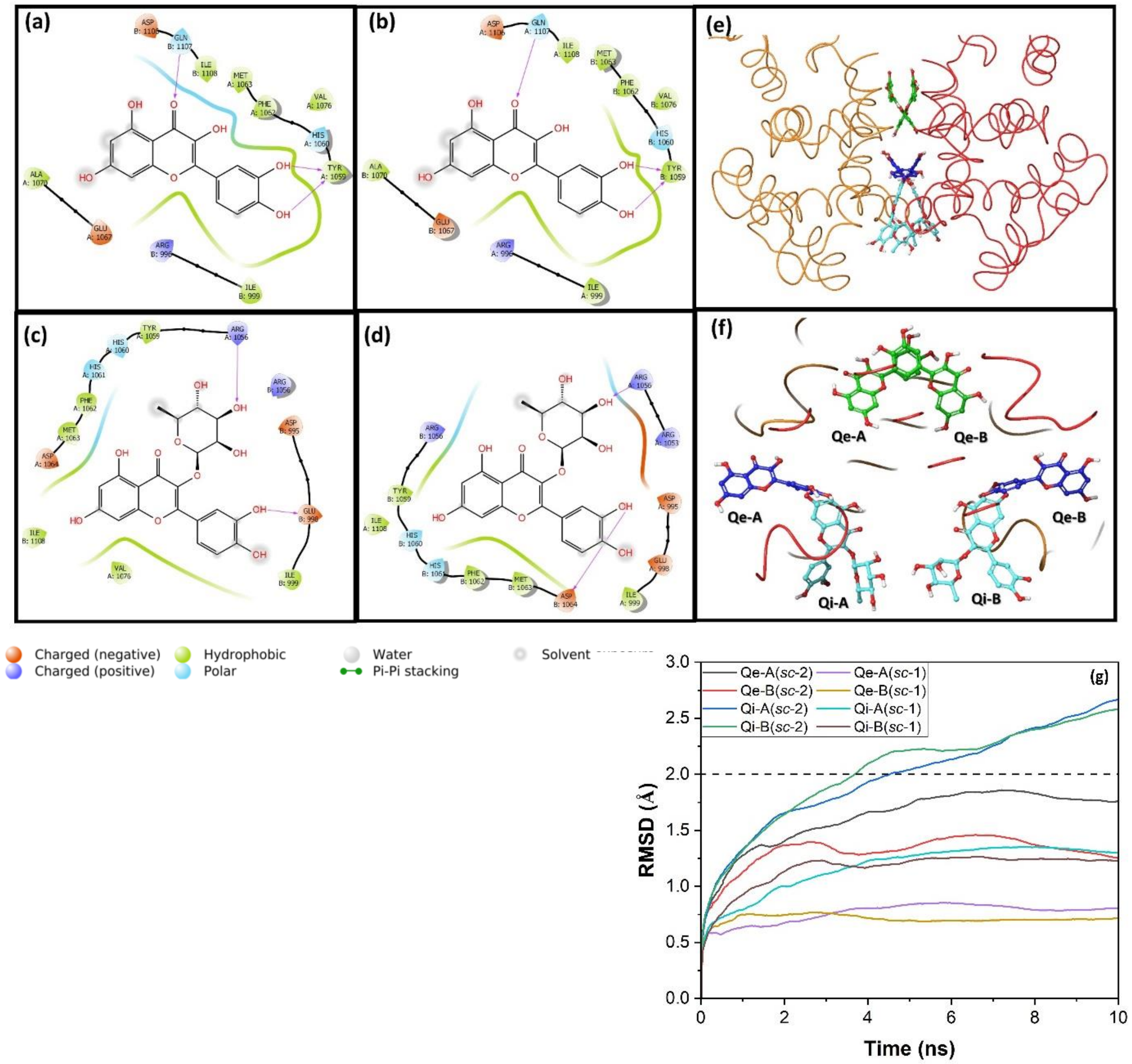

Supplementary Figure 3. Binding and stability of Qe and Qi to the dimer disruptor site 2 in *sc*Ire1p dimers. 2D interaction diagram showing the interacting residues with **(a)** Qe-A, **(b)** Qe-B, **(c)** Qi-A, and **(d)** Qi-B molecules docked into binding site *sc-2*. **(e-f)** The binding poses of Qe and Qi molecules in binding *sc-2* compared to Qe molecules (green) bound in site *sc-1*. **(g)** Average RMSD curves (calculated over 10 independent well-tempered metadynamics simulations) for the Qe and Qi molecules in binding sites *sc-1* and *sc-2*.

Qe and Qi bind with moderate strength to the ATP binding site in *h*IRE1.

The grid box for docking to the ATP binding site was generated on the co-crystalized ligand (Staurosporine) bound to chain A of *h*IRE1 dimer (4YZC) and the Qe and Qi molecules were flexibly docked. The docking score and free energy of binding values were calculated to be -8.71 and -42.74 kcal mol^{-1} for Qe, respectively, and the corresponding values for Qi are -11.31 and -57.04 kcal mol^{-1}, respectively. Supplementary Figure 4 illustrates the binding modes and the surrounding residues interacting with the ligands. A series of BPMD simulations were conducted to further evaluate the stability of the ligands. The results are presented in Supplementary Figure 4f. The averaged ligand RMSD curves with PoseScore values of 1.96 and 1.84 Å for the Qe and Qi molecules, respectively, demonstrate the moderate stability of the ligands bound into the kinase pocket. These results are in agreement with previous work showing that flavonols are weak/moderate inhibitors of a number of protein kinases [4]. To compare with the native kinase binder ADP, the *h*IRE1 monomer in complex with ADP and Mg metal ion (PDB ID: 4YZD) was prepared and superposed on chain-A of the *h*IRE1 dimer (PDV ID: 4YZC) from where the co-crystallized Staurosporine molecule was removed. The ADP molecule along with the Mg metal ion were transferred into the kinase domain and the structure further optimized and refined in implicit solvent using the OPLS4 force field. While the free energy of binding of the ADP molecule (-36.55 kcal mol^{-1}) is weaker than those seen for the Qe and Qi molecules, BPMD simulations resulted in a PoseScore value of 1.20 Å for ADP which is considerably better than those of Qe and Qi. The average ligand RMSD curves (Supplementary Figure 4f) confirm that Qe and Qi can barely compete with ADP in binding into the Kinase pocket of *h*IRE1.

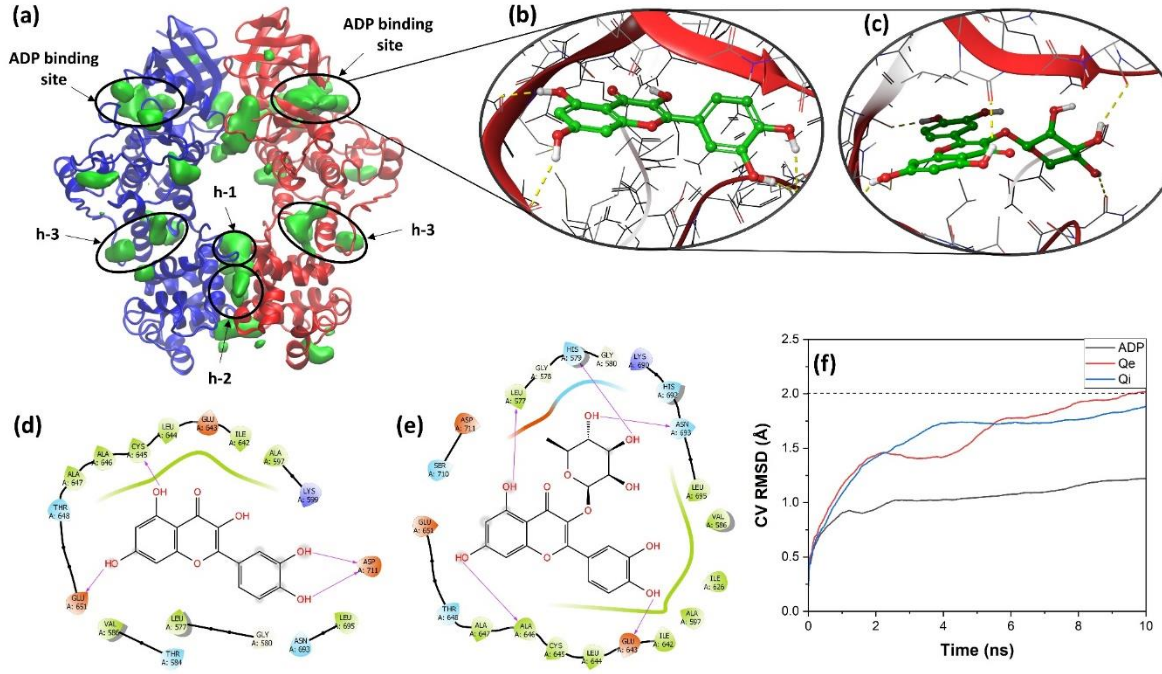

Supplementary Figure 4. Binding and stability of Qe and Qi in the ATP binding pocket of hIRE1. (a) Potential binding pockets in apo-*h*IRE1 dimer (pdb id:4YZC) identified by FPocket. Chain A and chain B are presented in red and blue colored ribbons, respectively. The green surfaces are the predicted binding sites (cavities) using FPocket depicted by a set of combined alpha spheres. The binding modes of the docked **(b)** Qe and **(c)** Qi molecules into the ADP binding site of *h*IRE1 dimer (chain A). 2D interaction diagrams showing the interacting residues with **(d)** Qe and **(e)** Qi molecules. **(f)** Average ligand RMSD curves (calculated over 10 independent BPMD simulations) for Qe, Qi and ADP in the ADP binding site.

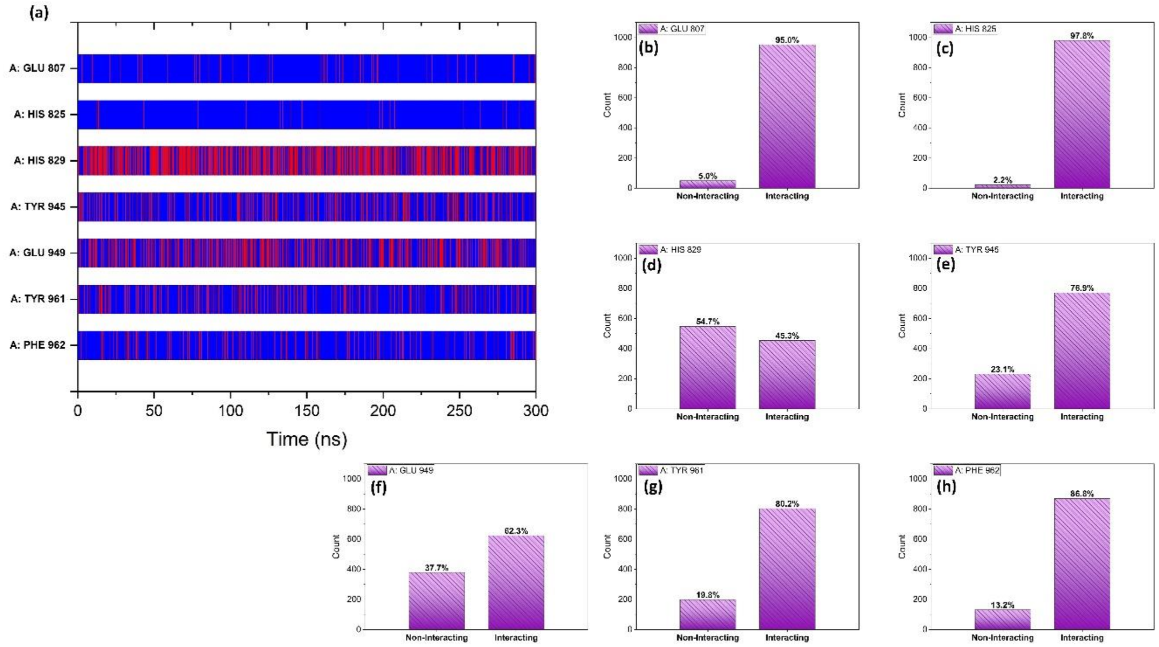

Supplementary Figure 5. (a) Time series of atomic interactions between Qe-A and the surrounding residues within binding site h-3 extracted from 300 ns MD simulation. The blue and red bars indicate interacting and non-interacting snapshot. Histogram of interactions per surrounding residue (b) Glu 807, (c) His 825, (d) His829, (e) Tyr 945, (f) Glu 949, (g) Tyr 961, and (h) Phe 962. The interaction types are indicated in Figure 4e.

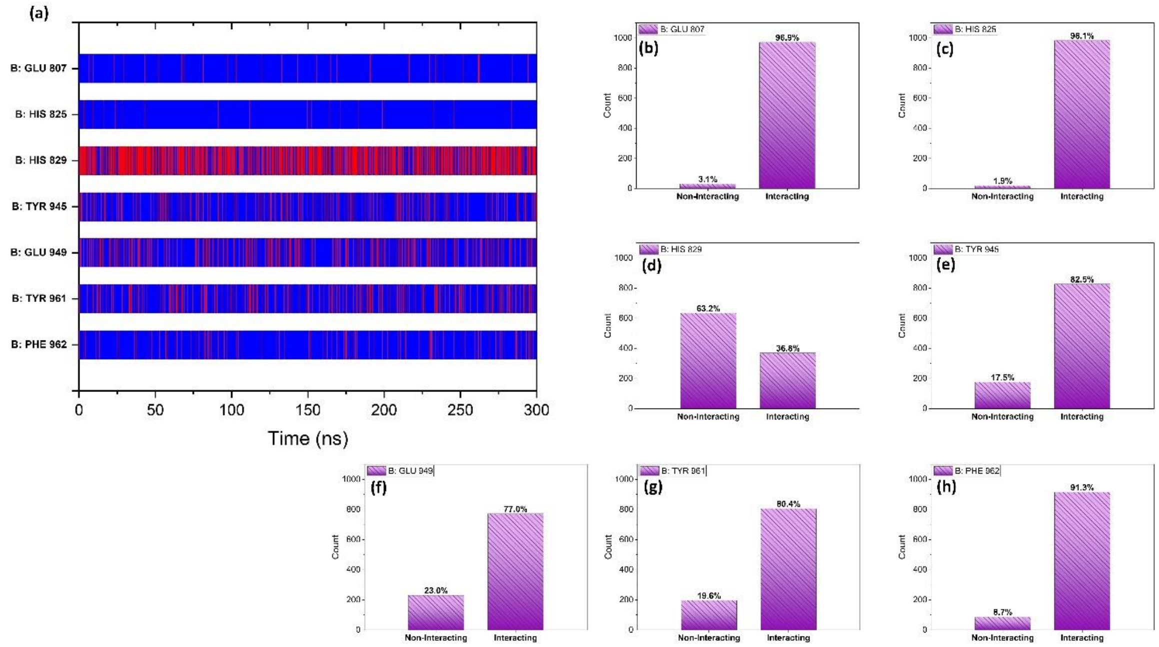

Supplementary Figure 6. (a) Time series of atomic interactions between Qe-B and the surrounding residues within binding site h-3 extracted from 300 ns MD simulation. The blue and red bars indicate interacting and non-interacting snapshot. Histogram of interactions per surrounding residue (b) Glu 807, (c) His 825, (d) His829, (e) Tyr 945, (f) Glu 949, (g) Tyr 961, and (h) Phe 962. The interaction types are indicated in Figure 4f.

Supplementary Note 1:

FPocket

FPocket [5, 6] is a protein pocket (cavity) detection algorithm based on Voronoi tessellation and the concept of alpha spheres. It uses of the QHULL library [7] to perform Voronoi tessellation to gather alpha spheres. Briefly, an alpha sphere is a sphere that contacts four atoms on its boundary and contains no internal atom. Alpha sphere radii reflect the local curvature defined by the four atoms: 4 atoms in a plane would correspond to an alpha sphere of infinite radius, and conversely, 4 atoms packed at the apex of a tetrahedron would lead to a value of radius close to that of the Van der Waals radius. For a protein, very small spheres are located within the protein, large spheres at the exterior, and clefts and cavities correspond to spheres of intermediate radii.

The FPocket algorithm can be divided in the following steps:

- Calculate alpha spheres on the whole protein
- Filter out alpha spheres with radius $m < R < M$, with $m = 3.0$ and $M = 6.0$ in the current implementation
- Cluster alpha sphere using a multiple linkage clustering algorithm
- Merge nearby pockets
- Remove very small pockets
- Score and rank pockets using scoring function that employs several pocket descriptors [8].

FPocket allows tracking protein pockets during MD simulation. Briefly, based on a set of aligned PDB structures, FPocket runs over each of MD snapshots and collects all resulting alpha spheres. Once done, a discrete grid will be created (1Å resolution) and superposed to all alpha spheres. The number of alpha spheres around each grid point will be counted, resulting in a pocket density grid, similar to an electron density map. For each grid point, FPocket counts the number of alpha spheres located around a sphere of 8 Å (default parameters). Thus, the more a cavity is conserved during the MD, the higher this value will be for a given grid point (alpha sphere density).

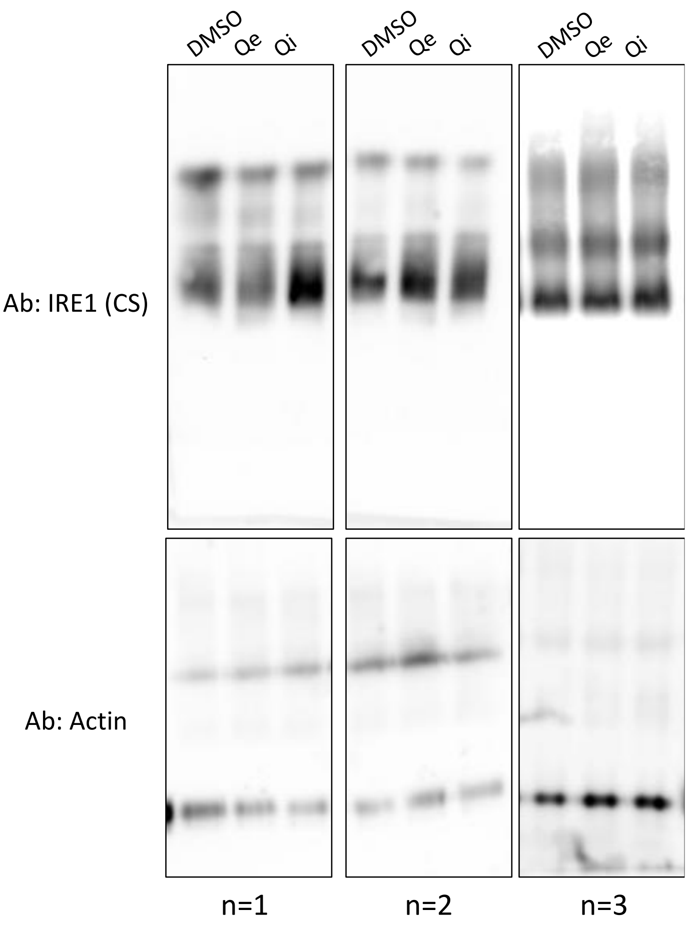

Supplementary Figure 7. Effects of Quercetin and Quercitrin on IRE1 activity. HEK293T cells overexpressing IRE1 were treated with vehicle (DMSO), 10μM Qe or 10μM Qi for 2 hours. Detection of IRE1 containing complexes was achieved using Western blot following protein separation by native gel electrophoresis. Actin was used as a loading control. The gel images of 3 replicates are shown. Full gel images of the Western blots as well as raw data for the MST experiments are provided as Supplementary Data.

Supplementary References

[1] L. Fusani, D.S. Palmer, D.O. Somers, I.D. Wall, Exploring ligand stability in protein crystal structures using binding pose metadynamics, Journal of Chemical Information and Modeling, 60 (2020) 1528-1539.

[2] A.J. Clark, P. Tiwary, K. Borrelli, S. Feng, E.B. Miller, R. Abel, R.A. Friesner, B.J. Berne, Prediction of protein–ligand binding poses via a combination of induced fit docking and metadynamics simulations, Journal of chemical theory and computation, 12 (2016) 2990-2998.

[3] R.L. Wiseman, Y. Zhang, K.P. Lee, H.P. Harding, C.M. Haynes, J. Price, F. Sicheri, D. Ron, Flavonol activation defines an unanticipated ligand-binding site in the kinase-RNase domain of IRE1, Molecular cell, 38 (2010) 291-304.

[4] B. Baby, P. Antony, W. Al Halabi, Z. Al Homedi, R. Vijayan, Structural insights into the polypharmacological activity of quercetin on serine/threonine kinases, Drug Design, Development and Therapy, (2016) 3109-3123.

[5] V. Le Guilloux, P. Schmidtke, P. Tuffery, Fpocket: an open source platform for ligand pocket detection, BMC bioinformatics, 10 (2009) 1-11.

[6] P. Schmidtke, A. Bidon-Chanal, F.J. Luque, X. Barril, MDpocket: open-source cavity detection and characterization on molecular dynamics trajectories, Bioinformatics, 27 (2011) 3276-3285.

[7] B. Barber, H. Huhdanpaa, Qhull, The Geometry Center, University of Minnesota, http://www.geom. umn. edu/software/qhull, (1995).

[8] V. Le Guilloux, P. Schmidtke, P. Tuffery, Fpocket: An open source platform for ligand pocket detection, BMC Bioinformatics, 10 (2009) 168.
