# Supplementary material for: Different binding modalities of quercetin to inositol-requiring enzyme 1 of S. cerevisiae and human lead to opposite regulation
Source: Commun Chem. 2024 Jan 5;7:6. doi: 10.1038/s42004-023-01092-0 (PMC10767055; doi:10.1038/s42004-023-01092-0)
Supplement: Supplementary file 2 — Description of Additional Supplementary Files [file 42004_2023_1092_MOESM2_ESM.pdf]

Description of Additional Supplementary Files

File name: Supplementary Data 1

Description: Unprocessed gels for supplementary Figure 7

File name: Supplementary Data 2

Description: The output files for MST experimental measurements

File name: Supplementary Data 3

Description: The output files for MST Kd determination
