# Supplementary figures and images for: Different binding modalities of quercetin to inositol-requiring enzyme 1 of S. cerevisiae and human lead to opposite regulation
Source: Commun Chem. 2024 Jan 5;7:6. doi: 10.1038/s42004-023-01092-0 (PMC10767055; doi:10.1038/s42004-023-01092-0)

Ab: IRE1 (CS)

| <u>n=1</u> |    |    | <u>n=2</u> |    |    |
|------------|----|----|------------|----|----|
| DMSO       | Qe | Qi | DMSO       | Qe | Qi |

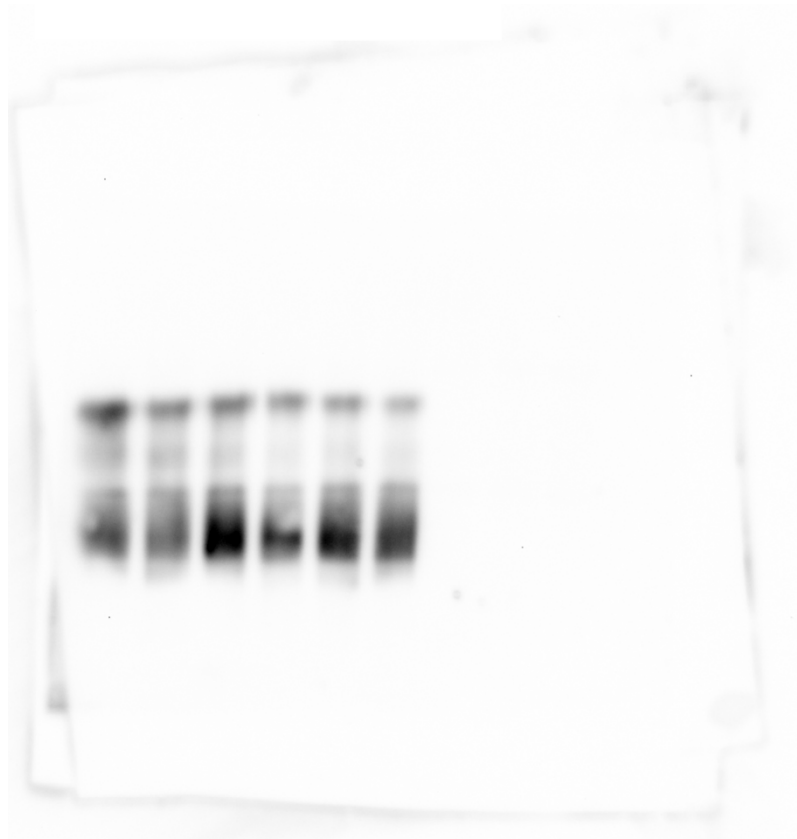

|   |   |   | <u>n=3</u> |    |    |   |   |   |
|---|---|---|------------|----|----|---|---|---|
| X | X | X | DMSO       | Qe | Qi | X | X | X |

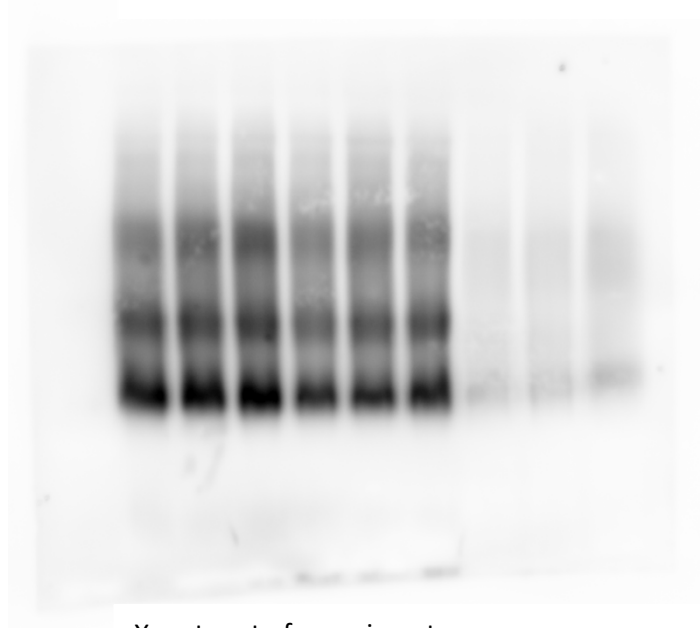

X=not part of experiment

Ab: Actin

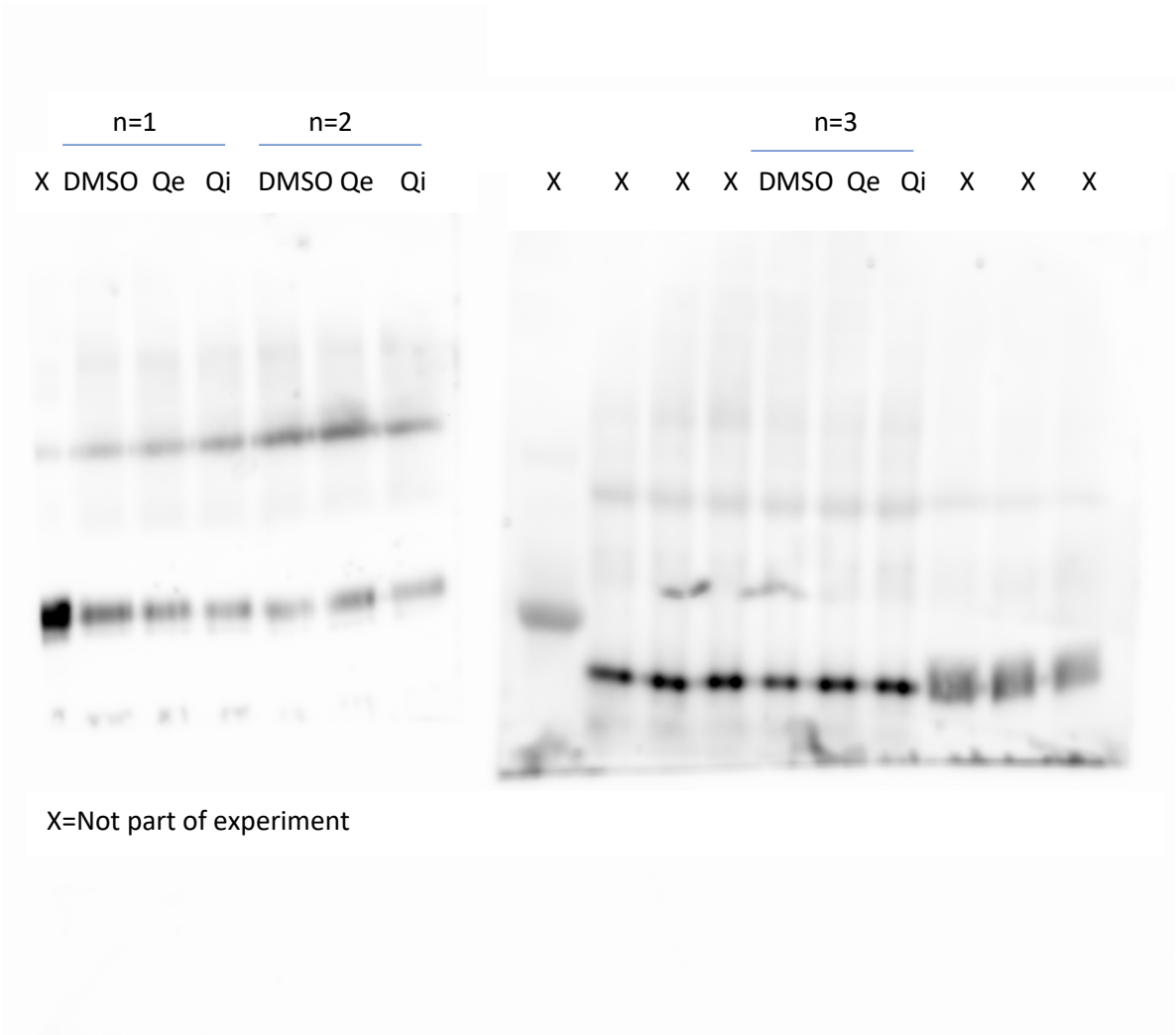

Supplement: Supplementary file 3 — Supplementary Data 1 [file 42004_2023_1092_MOESM3_ESM.pdf]
