# Supplementary material for: Different binding modalities of quercetin to inositol-requiring enzyme 1 of S. cerevisiae and human lead to opposite regulation
Source: Commun Chem. 2024 Jan 5;7:6. doi: 10.1038/s42004-023-01092-0 (PMC10767055; doi:10.1038/s42004-023-01092-0)
Supplement: Supplementary file 4 — Supplementary Data 2 [file 42004_2023_1092_MOESM4_ESM.pdf]

Report

Ntp-Filename: XG4-015
Experiment Name: DMSO_n2 2/27/2023 1:31:08 PM
MST Power: 40% , LED Power: 70%

Normalized Fluorescence Timetrace

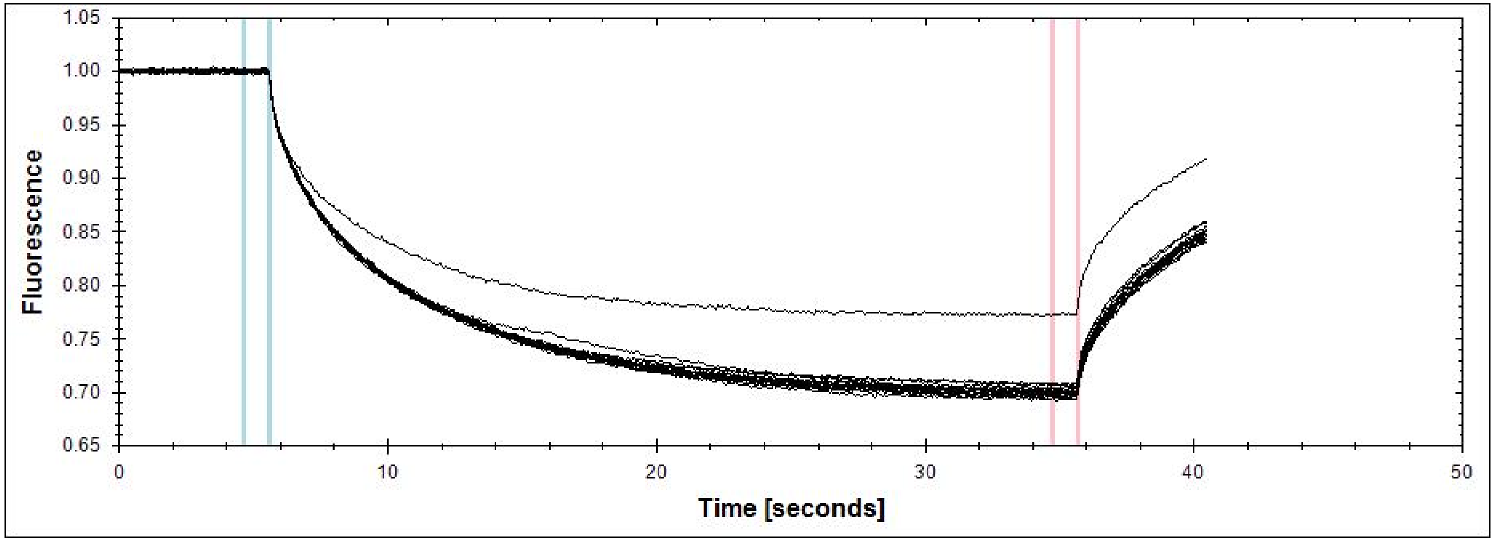

hot region: HotStart=4.62 HotLength=0.97
cold region ColdStart=34.72 ColdLength=0.97

Capillary-Scan: 0

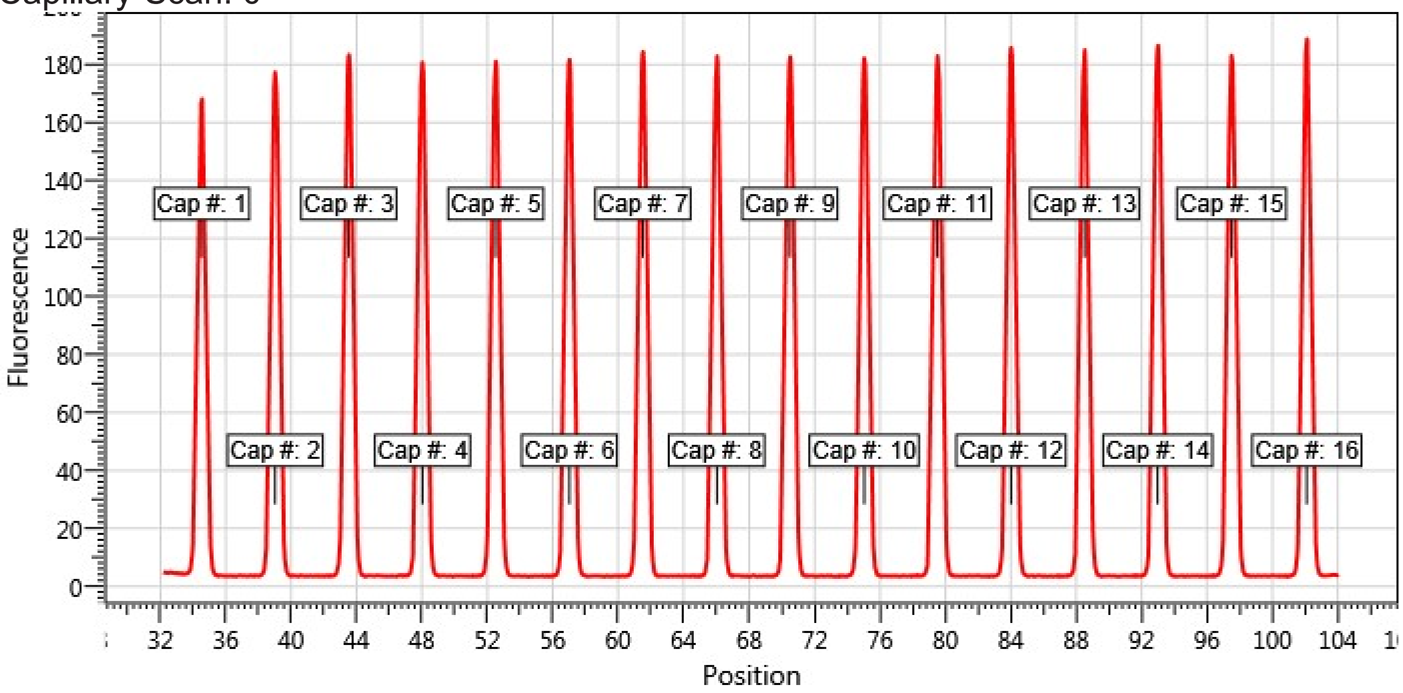

Experiments measured with this Cap-Scan

KIRA8_n3

Thermophoresis with Temperature Jump

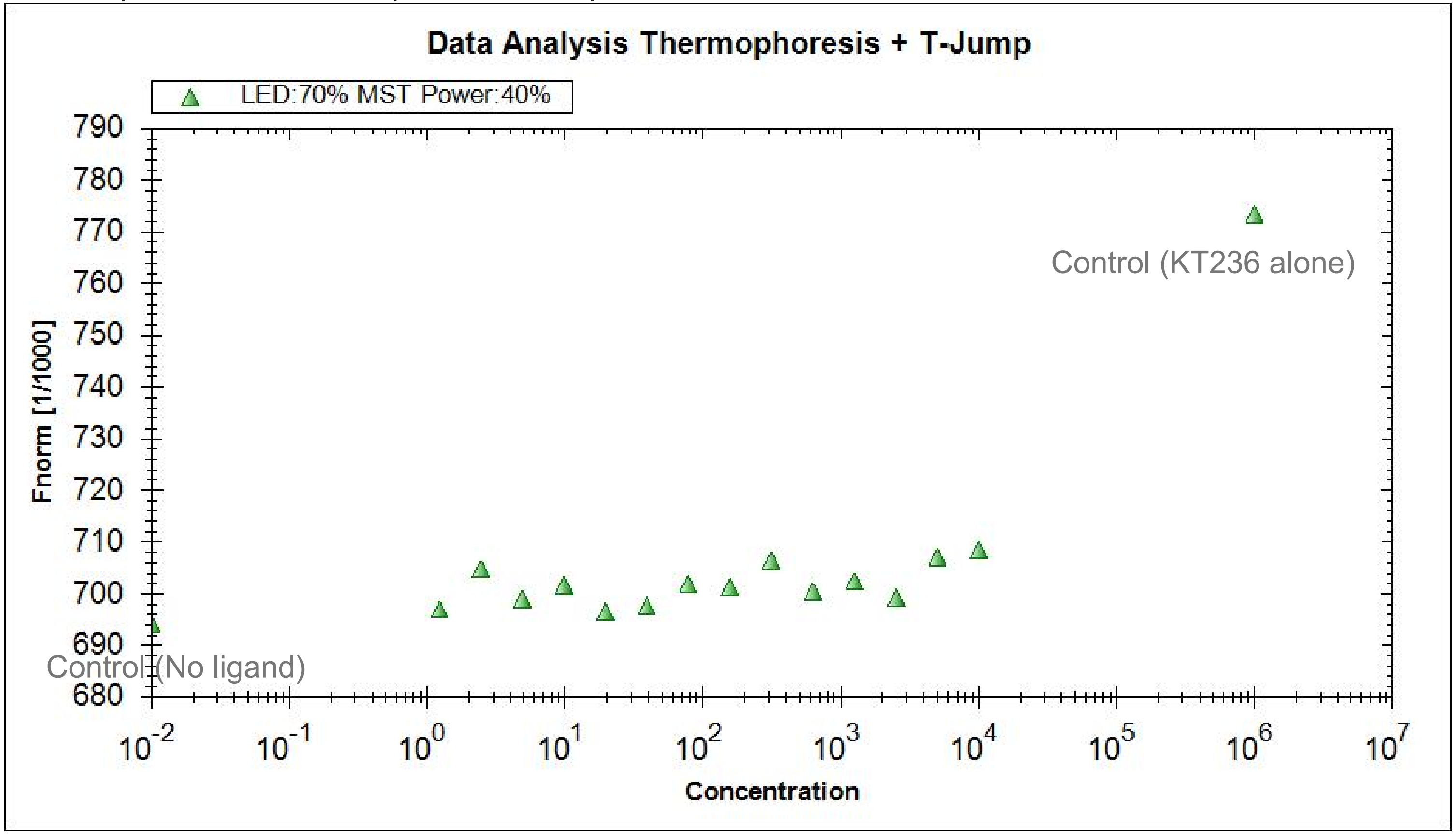

Data

Concentration	Fnorm [1/1000]	
0.01	694.19	Control (No DMSO, KT236 (10nM) + IRE1 (180 nM))
1.22	697.02	
2.44	704.83	
4.88	699.06	
9.77	701.75	
19.53	696.57	
39.06	697.67	
78.13	702.01	
156.25	701.38	
312.50	706.51	
625.00	700.51	
1250.00	702.41	
2500.00	699.24	
5000.00	707.14	
10000.00	708.48	
1000000.00	773.47	Control (KT236 alone, 10 nM)

Report

Ntp-Filename: XG4-015
Experiment Name: DMSO_n3 2/27/2023 4:33:34 PM
MST Power: 40% , LED Power: 70%

Normalized Fluorescence Timetrace

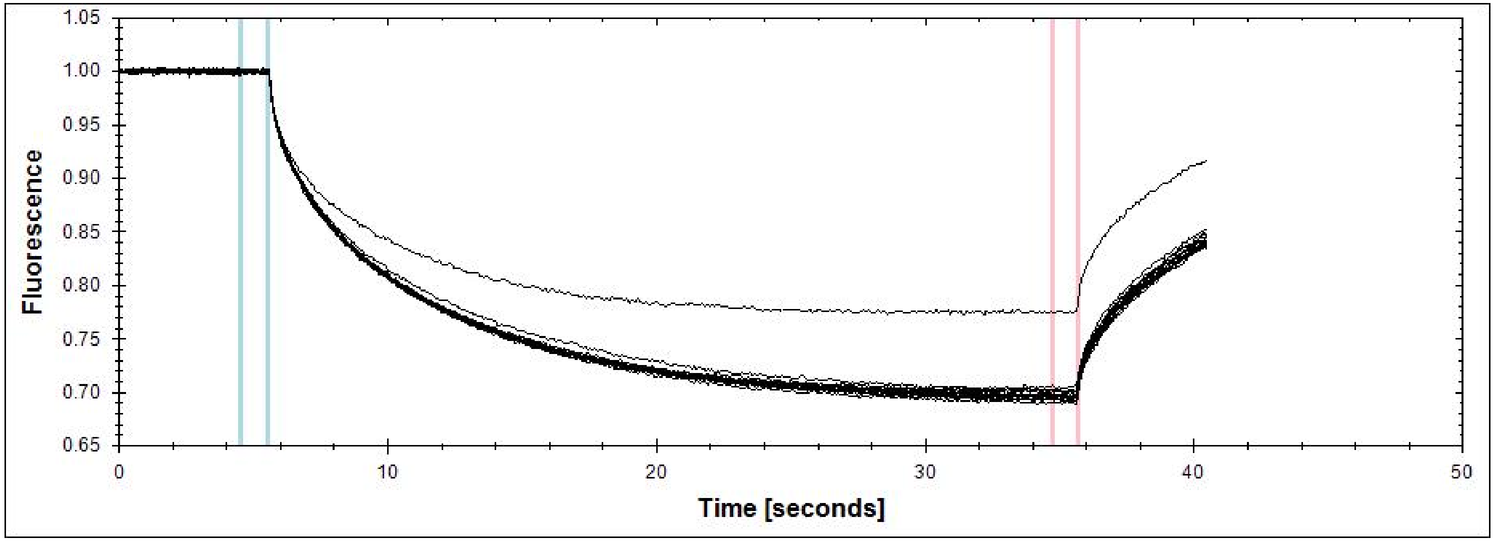

hot region: HotStart=4.55 HotLength=0.97
cold region ColdStart=34.72 ColdLength=0.97

Capillary-Scan: 0

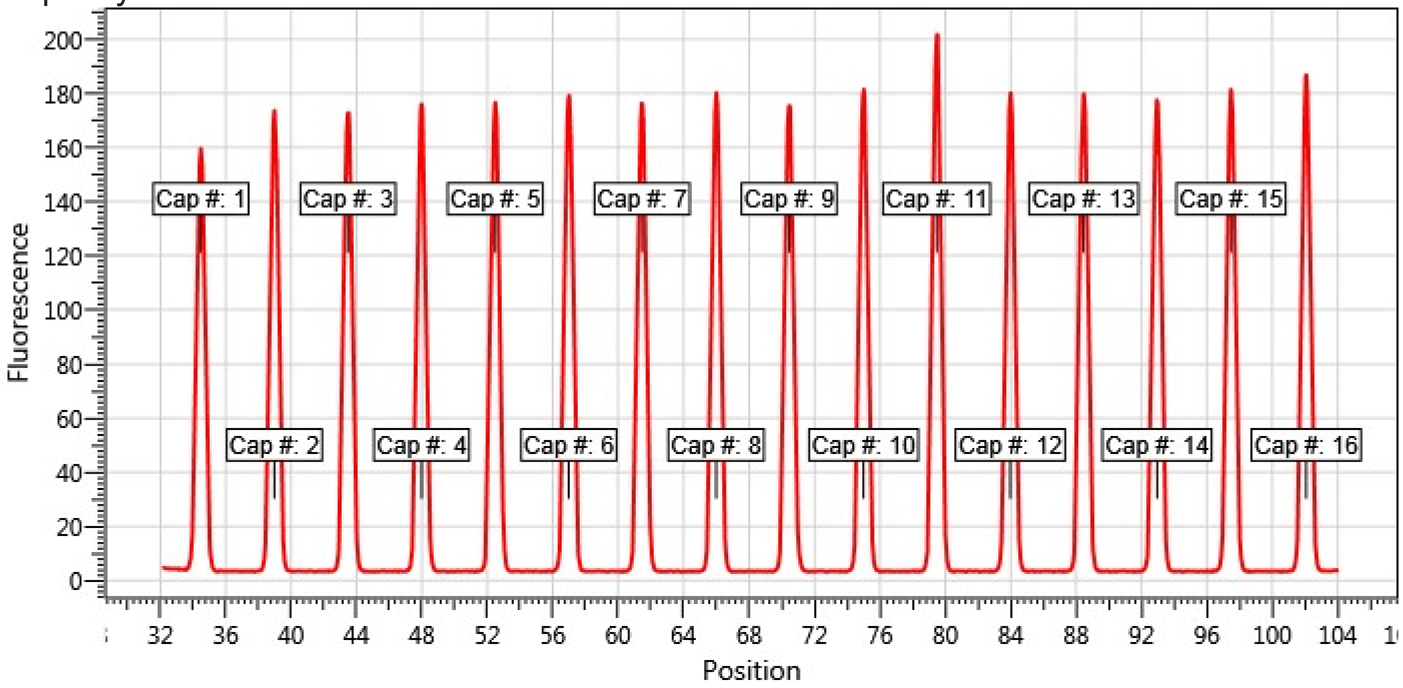

Experiments measured with this Cap-Scan

KIRA8_n3

Thermophoresis with Temperature Jump

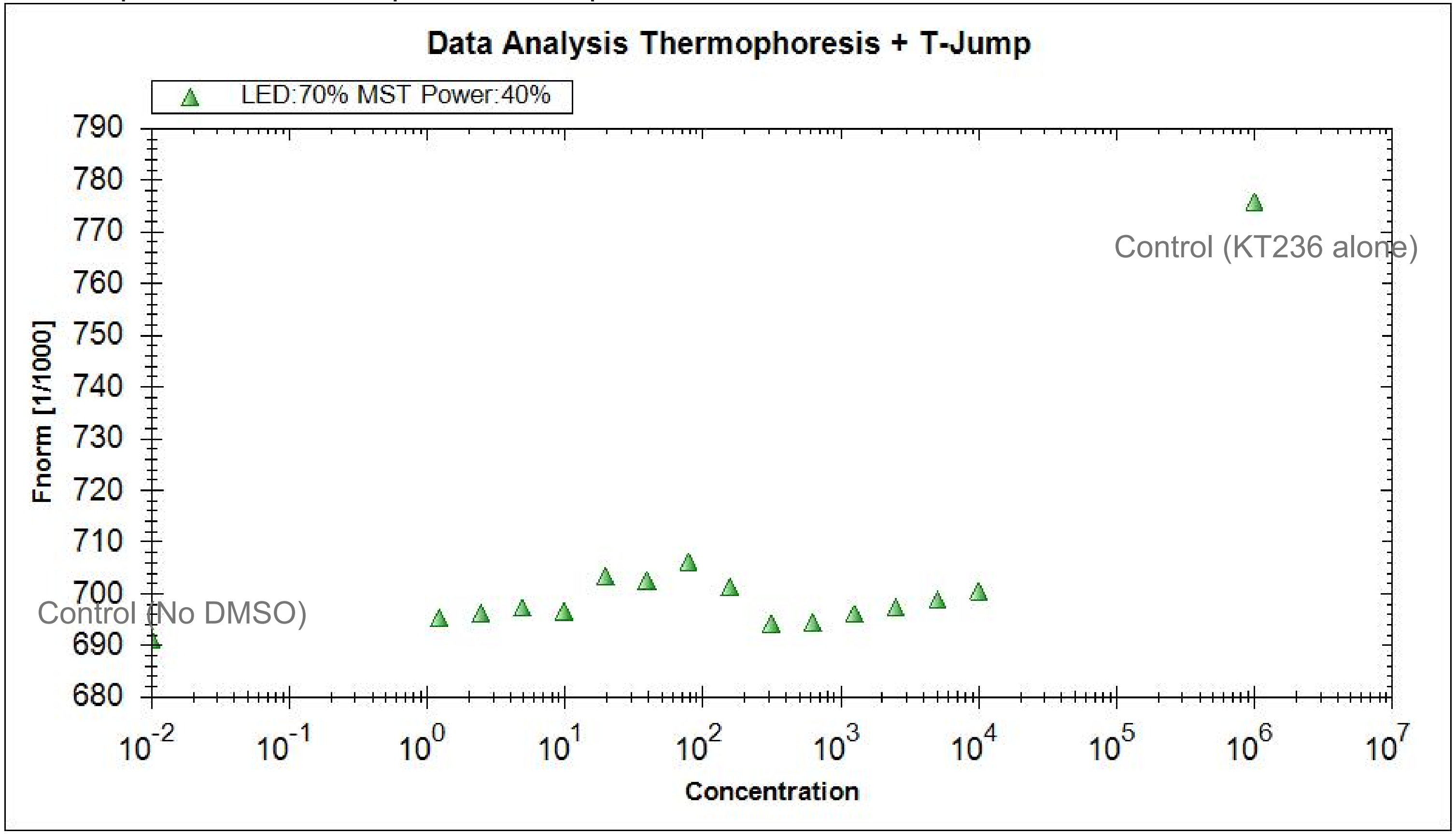

Data

Concentration	Fnorm [1/1000]	
0.01	691.17	Control (No DMSO, KT236 (10nM) + IRE1 (180 nM))
1.22	695.35	
2.44	696.27	
4.88	697.38	
9.77	696.67	
19.53	703.42	
39.06	702.55	
78.13	706.28	
156.25	701.36	
312.50	694.23	
625.00	694.42	
1250.00	696.15	
2500.00	697.33	
5000.00	698.84	
10000.00	700.59	
1000000.00	775.89	Control (KT236 alone, 10 nM)

Report

Ntp-Filename: XG4-014_MODIFIED_CONCENTRATION_.ntp
Experiment Name: XG4-014_KIRA8_replicate2 2/23/2023 11:17:08 AM
MST Power: 40% , LED Power: 70%

Normalized Fluorescence Timetrace

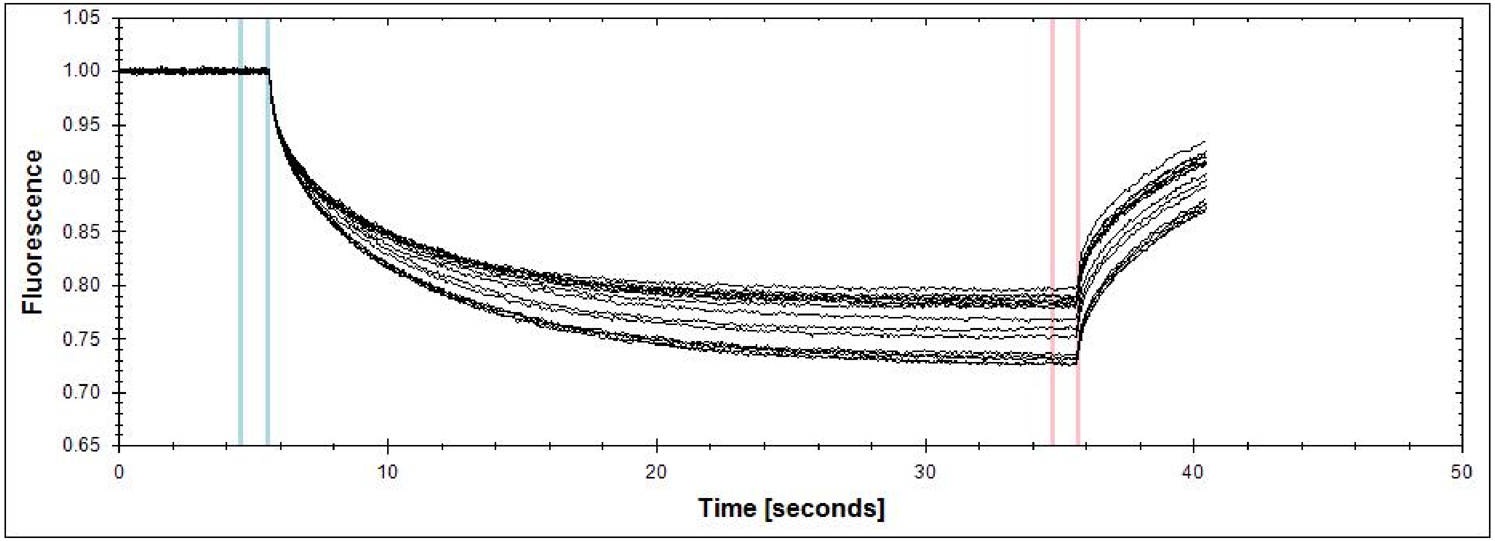

hot region: HotStart=4.55 HotLength=0.97
cold region ColdStart=34.72 ColdLength=0.97

Capillary-Scan: 0

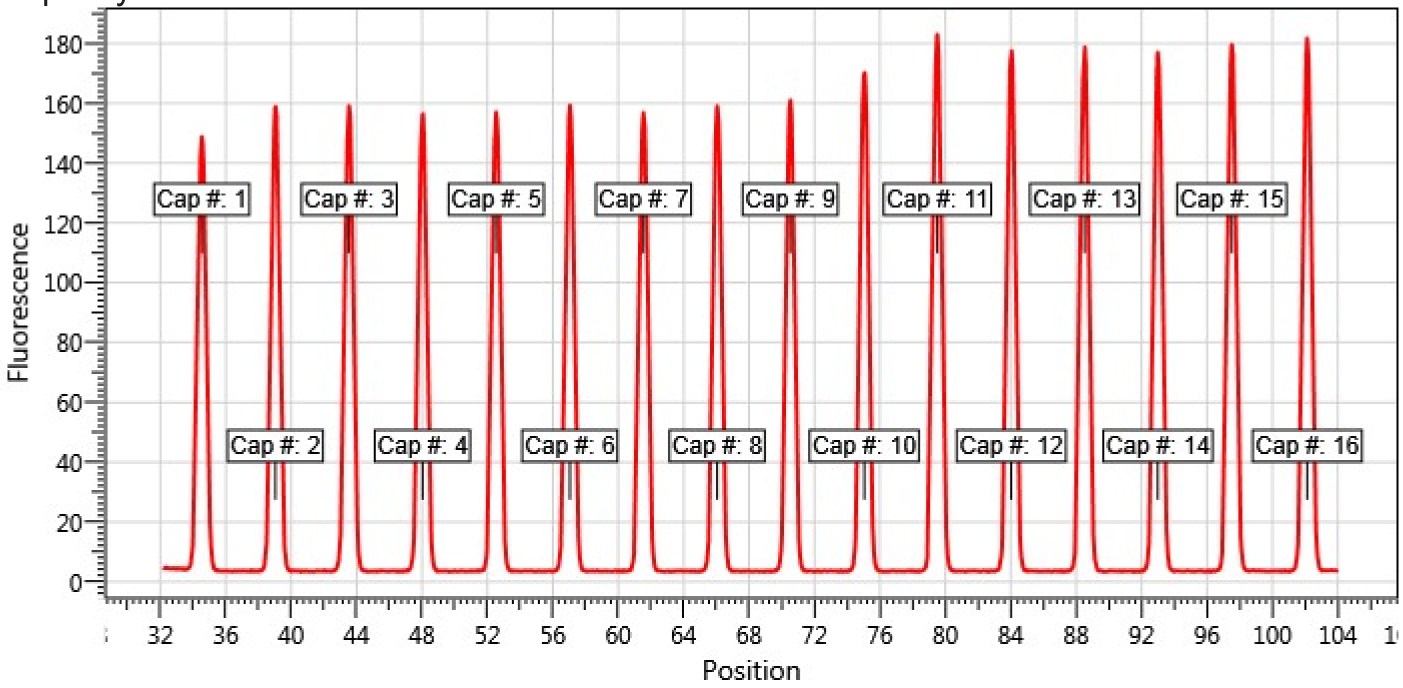

Experiments measured with this Cap-Scan

XG4-014_KIRA8_replicate2

Thermophoresis with Temperature Jump

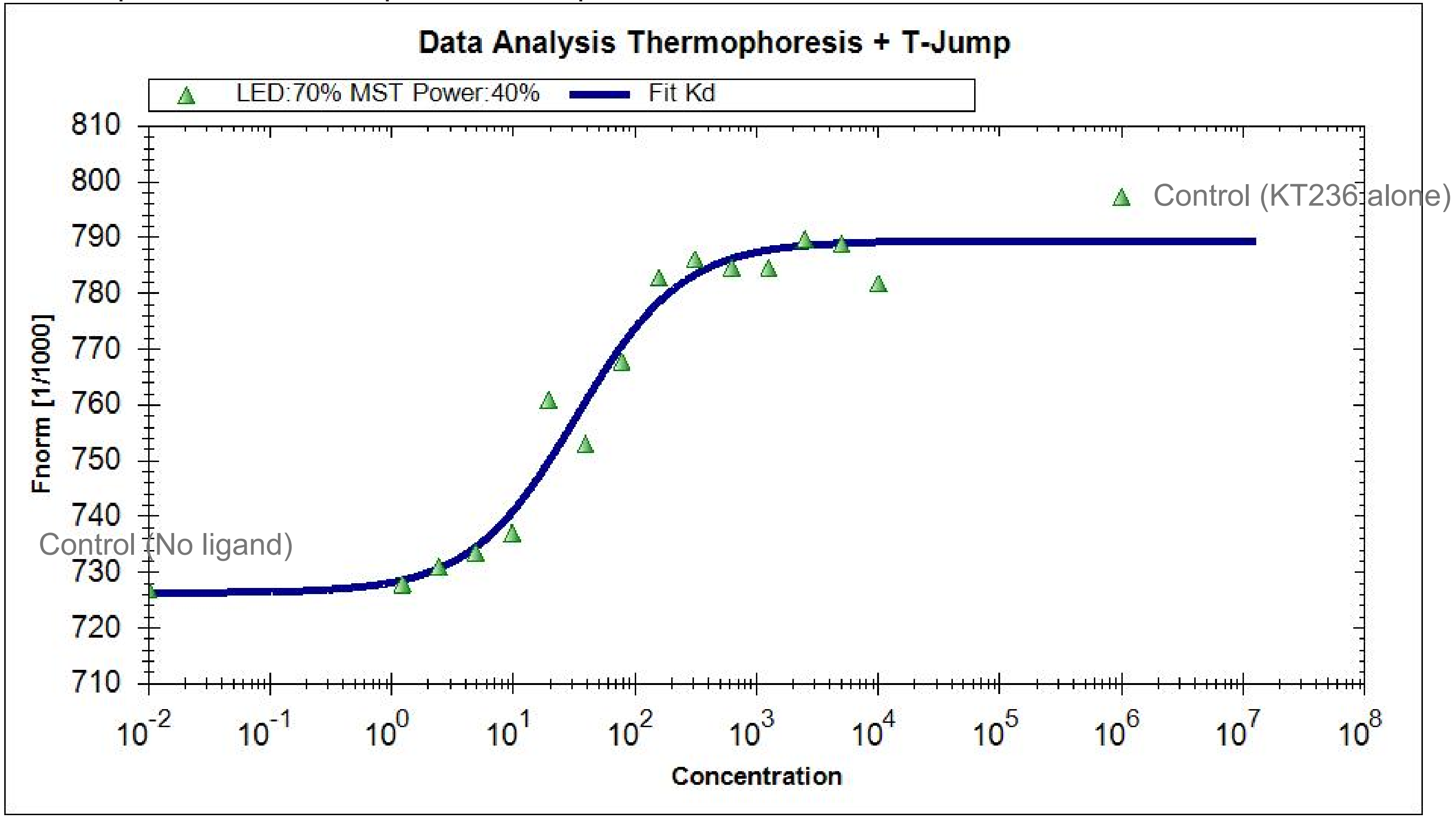

Fitting for Kd Formular

Fitted Parameter	Fitted Value
Dissociation Constant	32.9+/-3.06
Fluo.Conc	0.001
Bound	789.41
Unbound	726.33
Amplitude	63.08

Kd Formula (law of mass action)

f(c) = unbound + (bound-unbound) / 2 * (FluoConc + c + Kd - Sqrt((FluoConc + c + Kd)^2 - 4*FluoConc*c)

Data

Concentration	Fnorm [1/1000]	
0.01	726.98	Control (No ligand, KT236 (10nM) + IRE1 (180 nM))
1.22	727.91	
2.44	731.05	
4.88	733.48	
9.77	737.07	
19.53	760.98	
39.06	752.93	
78.13	767.81	
156.25	782.82	
312.50	786.16	
625.00	784.70	
1250.00	784.67	
2500.00	789.73	
5000.00	788.81	
10000.00	781.74	
1000000.00	797.39	Control (KT236 alone, 10 nM)

Report

Ntp-Filename: XG4-014_MODIFIED_CONCENTRATION_.ntp
Experiment Name: XG4-014_KIRA8_replicate3 2/23/2023 11:51:12 AM
MST Power: 40% , LED Power: 70%

Normalized Fluorescence Timetrace

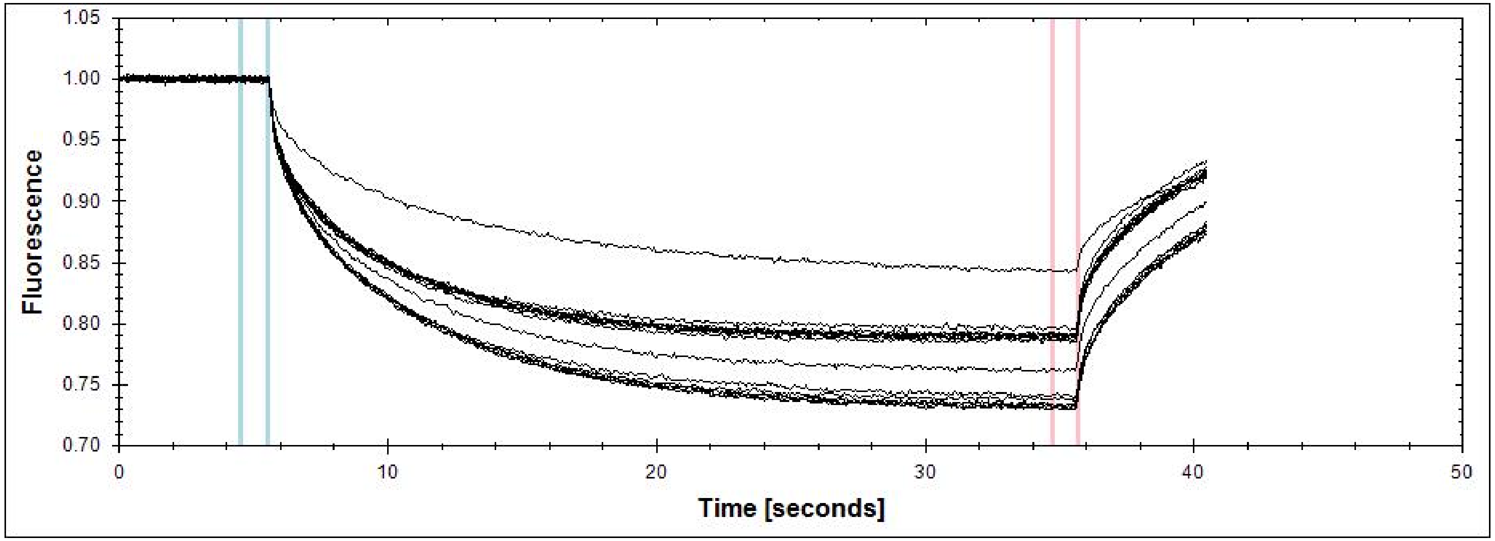

hot region: HotStart=4.55 HotLength=0.97
cold region ColdStart=34.72 ColdLength=0.97

Capillary-Scan: 0

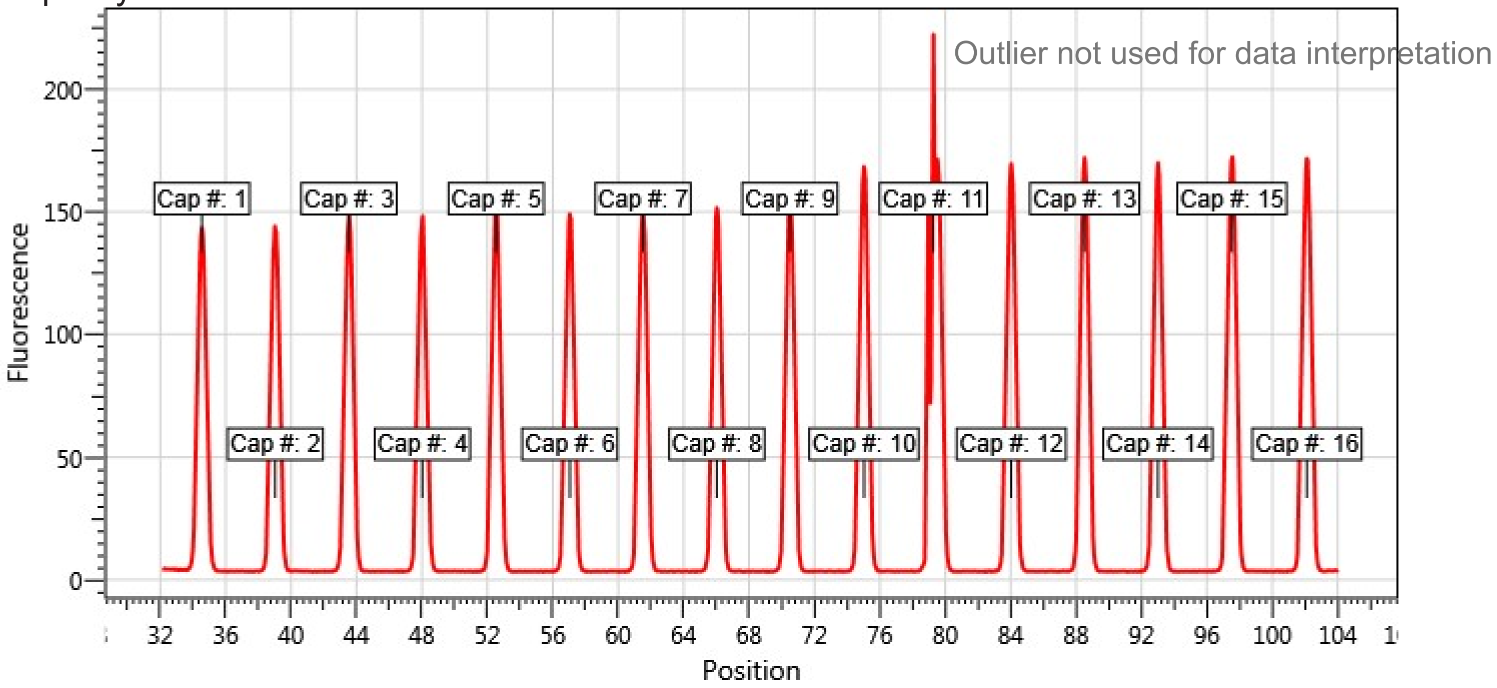

Experiments measured with this Cap-Scan

XG4-014_KIRA8_replicate2

Thermophoresis with Temperature Jump

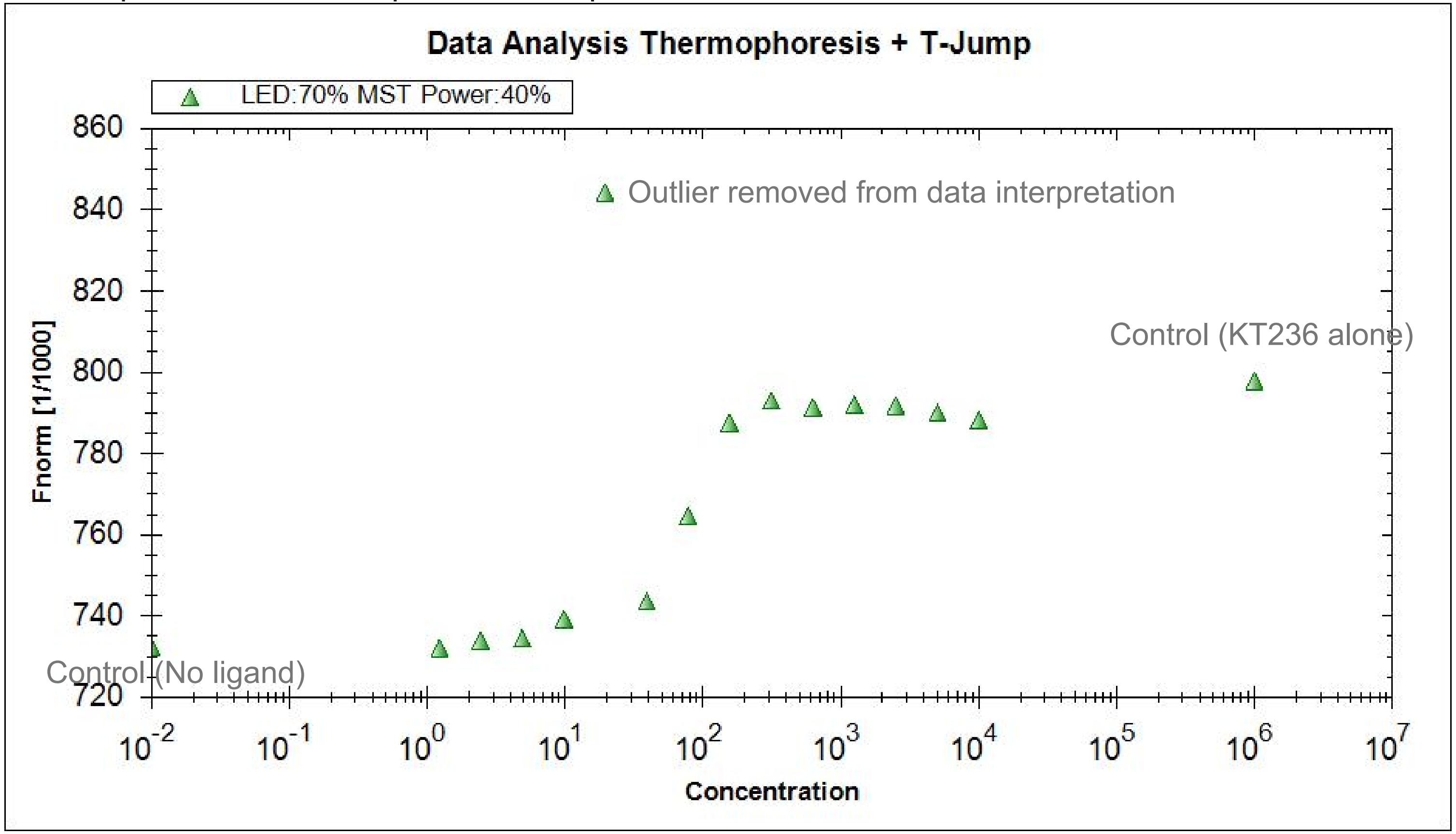

Data

Concentration	Fnorm [1/1000]	
0.01	732.21	Control (No ligand, KT236 (10nM) + IRE1 (180 nM))
1.22	732.14	
2.44	733.90	
4.88	734.56	
9.77	739.28	
19.53	844.31	
39.06	743.65	
78.13	764.72	
156.25	787.65	
312.50	793.06	
625.00	791.38	
1250.00	792.00	
2500.00	791.79	
5000.00	790.01	
10000.00	788.23	
1000000.00	797.89	Control (KT236 alone, 10 nM)

Report

Ntp-Filename: XG4-015
Experiment Name: KIRA8_n3 2/27/2023 12:21:18 PM
MST Power: 40% , LED Power: 70%

Normalized Fluorescence Timetrace

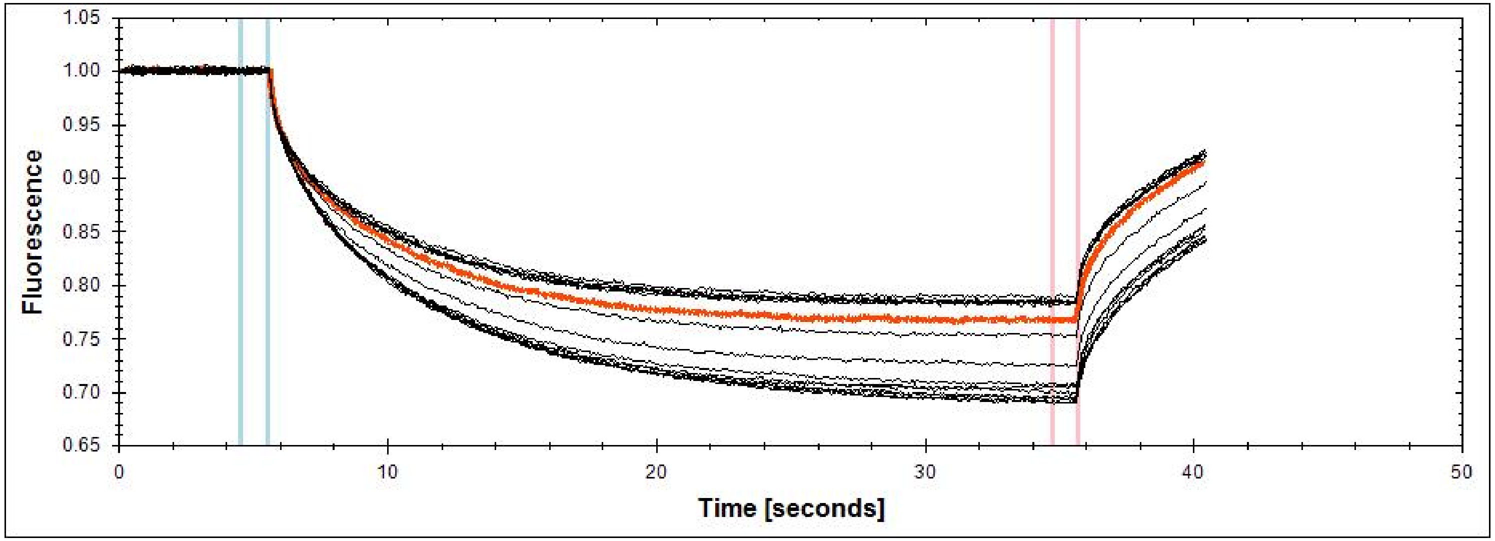

hot region: HotStart=4.55 HotLength=0.97
cold region ColdStart=34.72 ColdLength=0.97

Capillary-Scan: 0

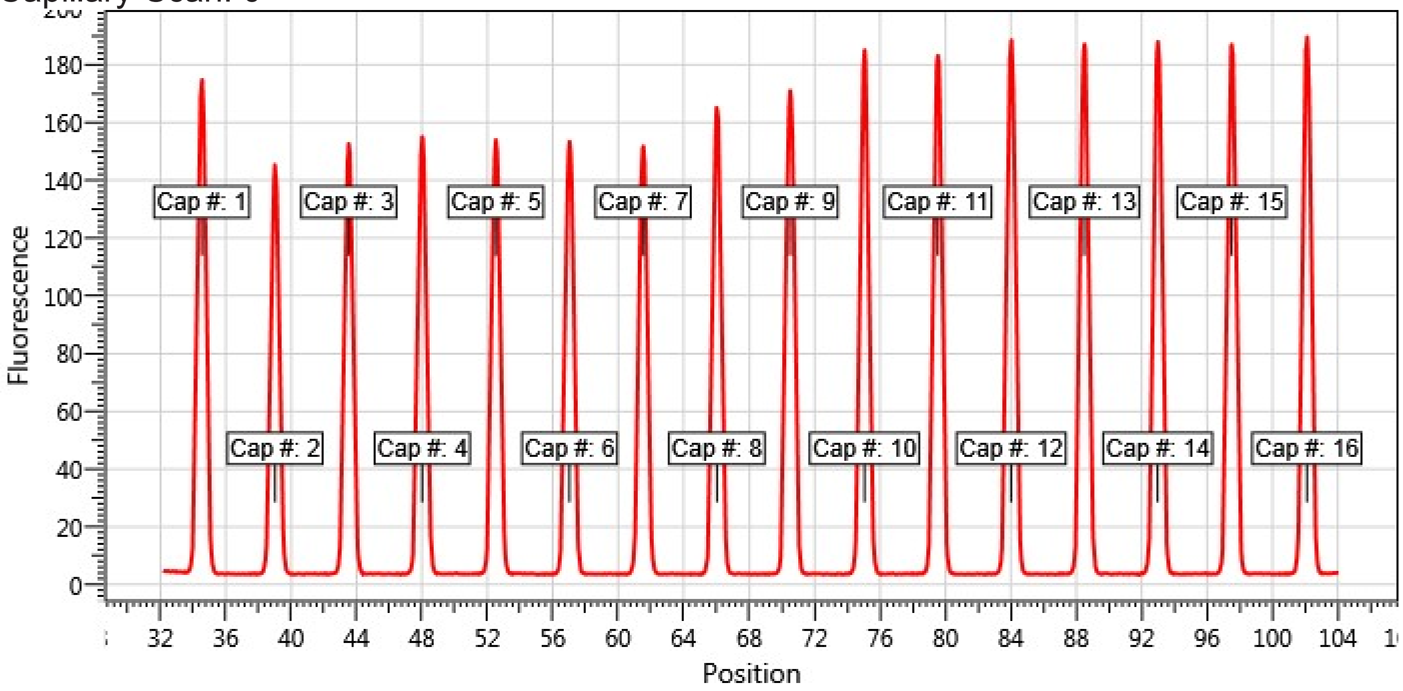

Experiments measured with this Cap-Scan

KIRA8_n3

Thermophoresis with Temperature Jump

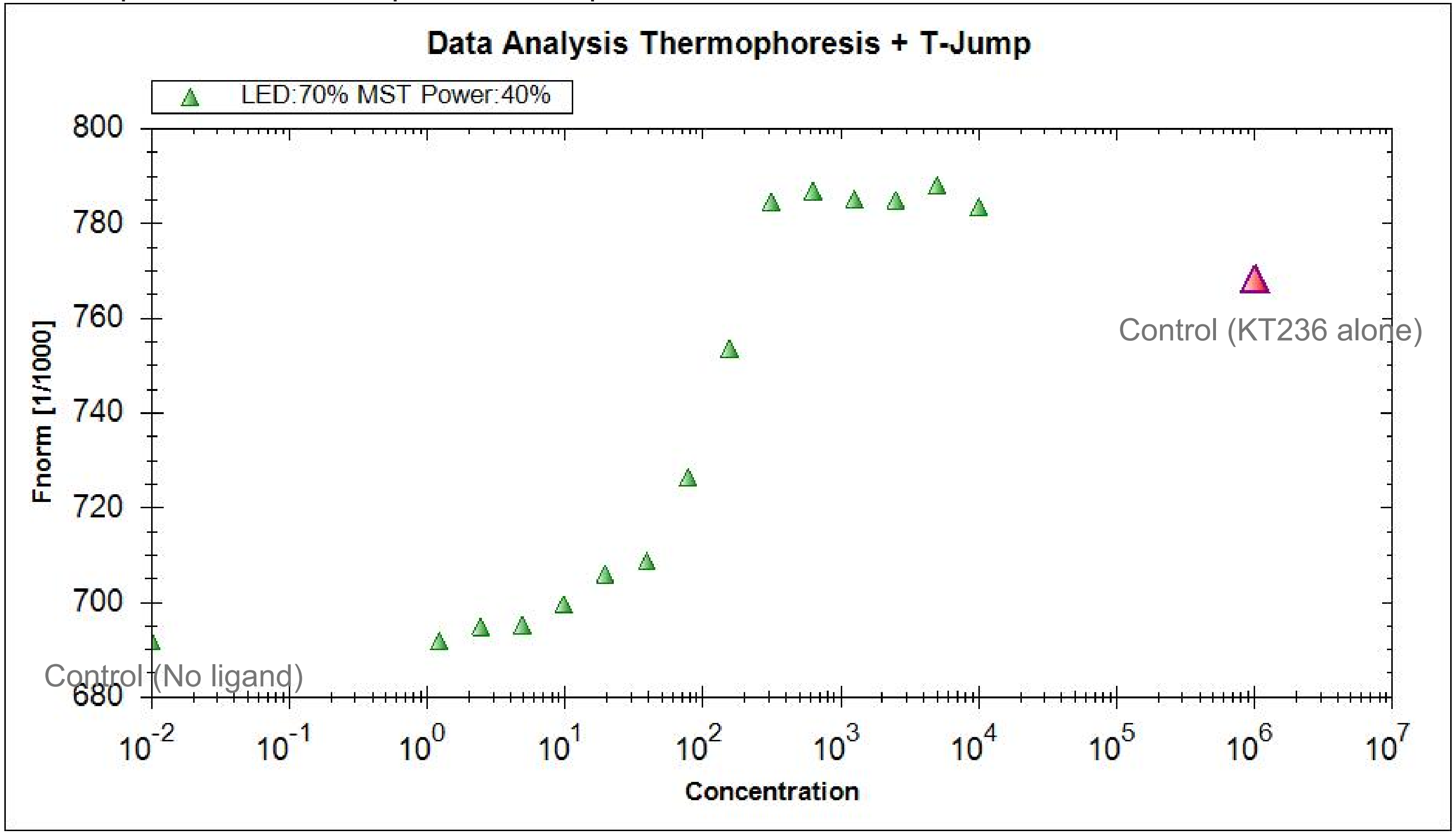

Data

Concentration	Fnorm [1/1000]	
0.01	691.84	Control (No ligand, KT236 (10nM) + IRE1 (180 nM))
1.22	691.77	
2.44	694.97	
4.88	695.22	
9.77	699.59	
19.53	706.02	
39.06	708.71	
78.13	726.49	
156.25	753.62	
312.50	784.52	
625.00	786.93	
1250.00	785.14	
2500.00	784.88	
5000.00	788.07	
10000.00	783.56	
1000000.00	768.45	Control (KT236 alone, 10 nM)

Report

Ntp-Filename: XG4-012
Experiment Name: Qe_100uM 2/22/2023 10:43:09 AM
MST Power: 40% , LED Power: 70%

Normalized Fluorescence Timetrace

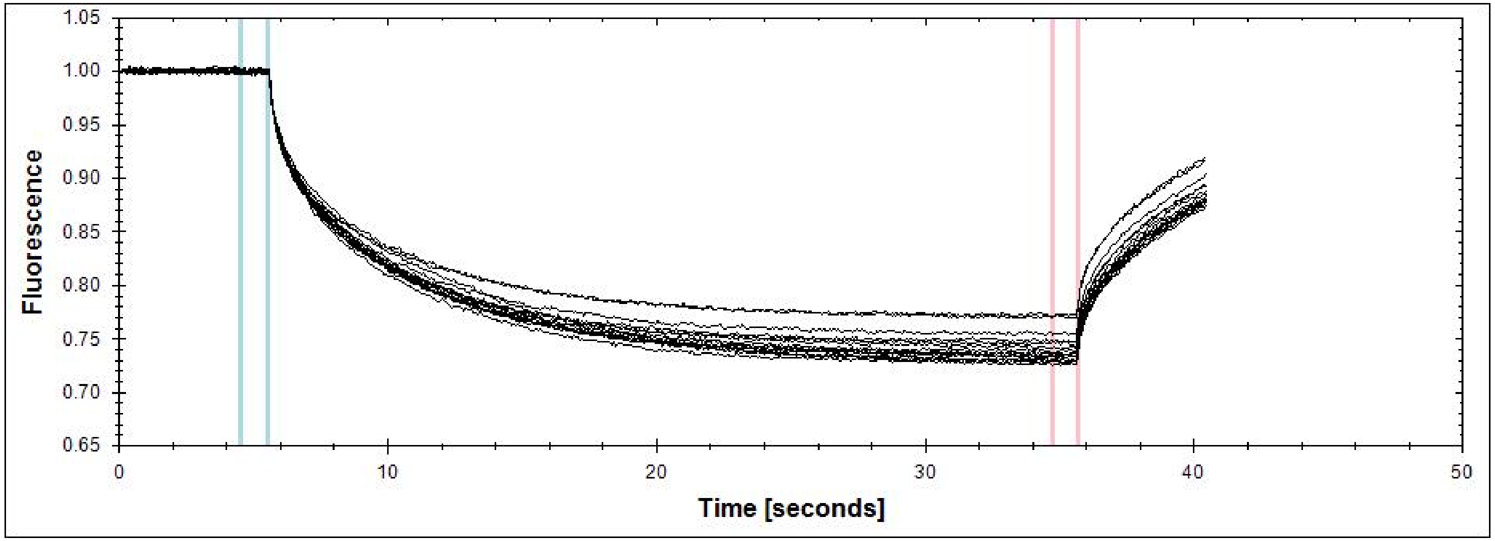

hot region: HotStart=4.55 HotLength=0.97
cold region ColdStart=34.72 ColdLength=0.97

Capillary-Scan: 0

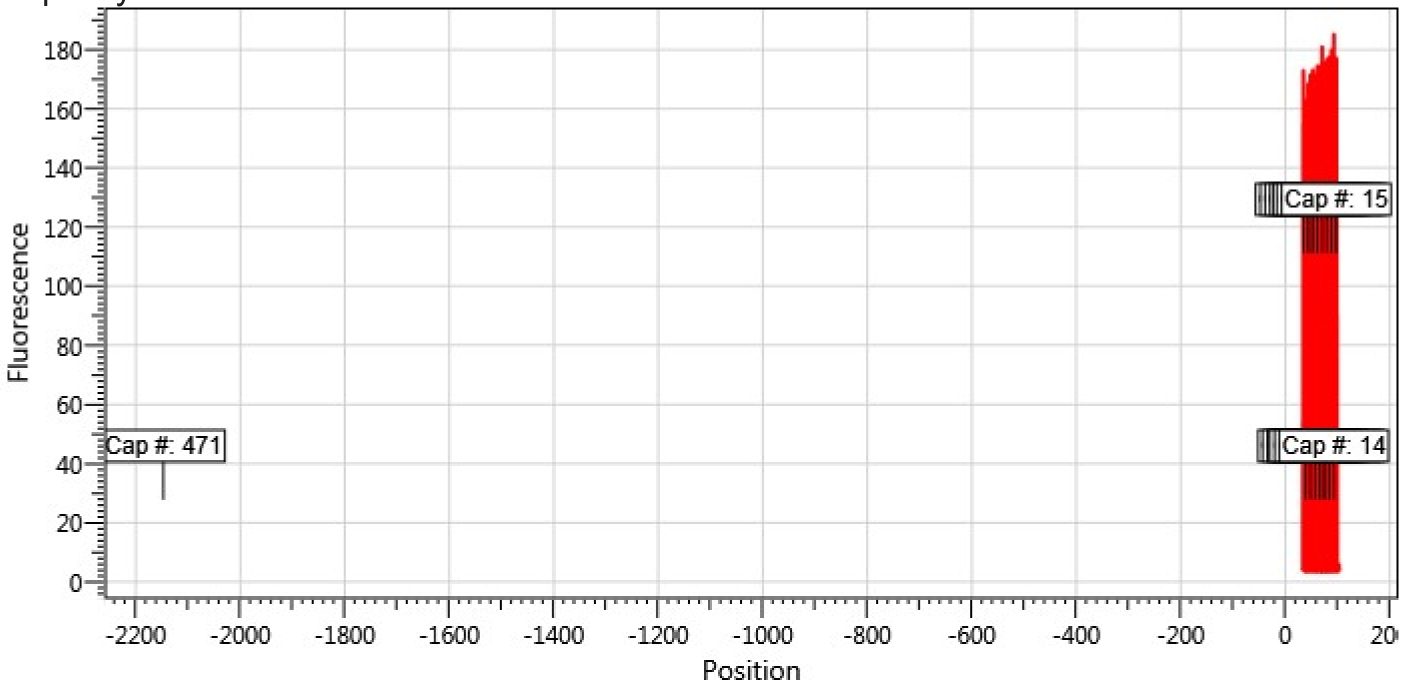

Experiments measured with this Cap-Scan

XG4-014_Qi_replicate3

Thermophoresis with Temperature Jump

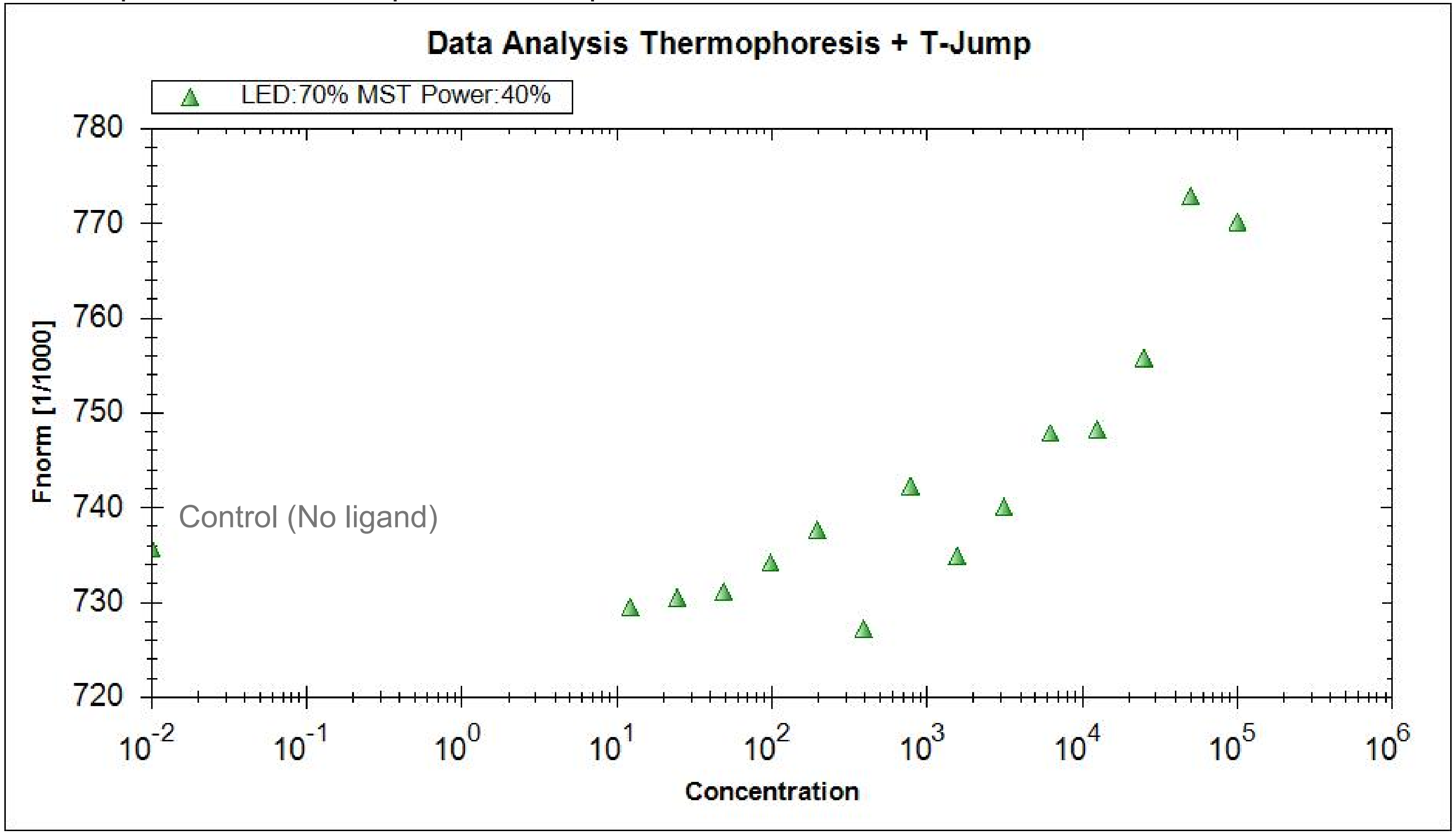

Data

Concentration	Fnorm [1/1000]	
0.01	735.73	Control (No ligand, KT236 (10nM) + IRE1 (180 nM))
12.21	729.52	
24.41	730.51	
48.83	731.19	
97.66	734.19	
195.31	737.62	
390.63	727.25	
781.25	742.27	
1562.50	734.91	
3125.00	740.05	
6250.00	747.87	
12500.00	748.31	
25000.00	755.84	
50000.00	772.85	
100000.00	770.19	

Report

Ntp-Filename: XG4-013
Experiment Name: Qe_100uM_replicate2 2/22/2023 2:01:19 PM
MST Power: 40% , LED Power: 70%

Normalized Fluorescence Timetrace

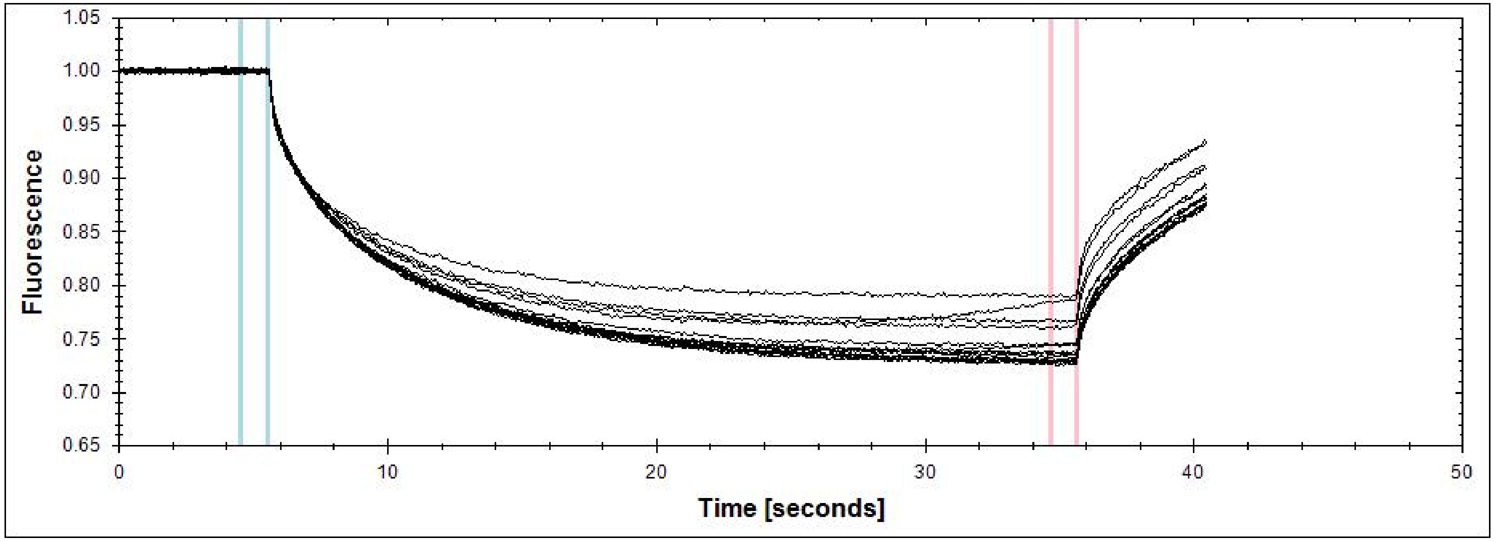

hot region: HotStart=4.55 HotLength=0.97
cold region ColdStart=34.65 ColdLength=0.97

Capillary-Scan: 0

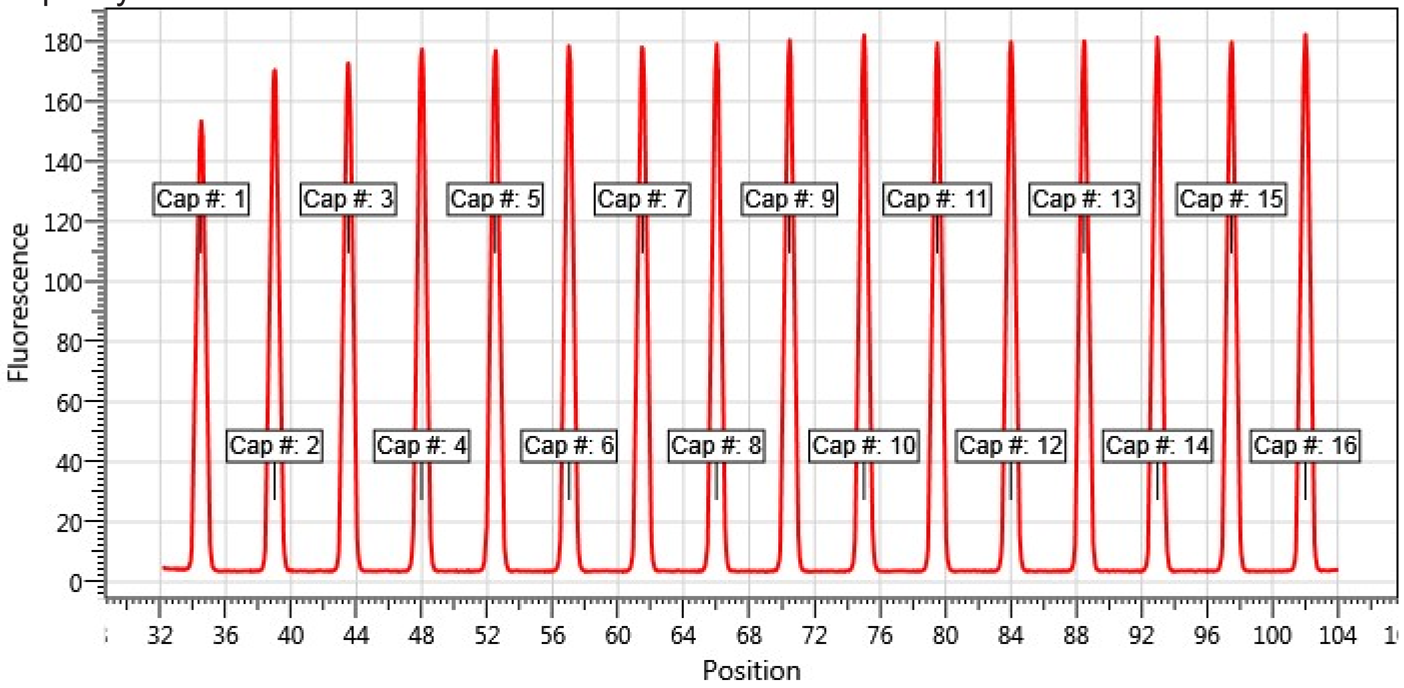

Experiments measured with this Cap-Scan

XG4-014_Qi_replicate3

Thermophoresis with Temperature Jump

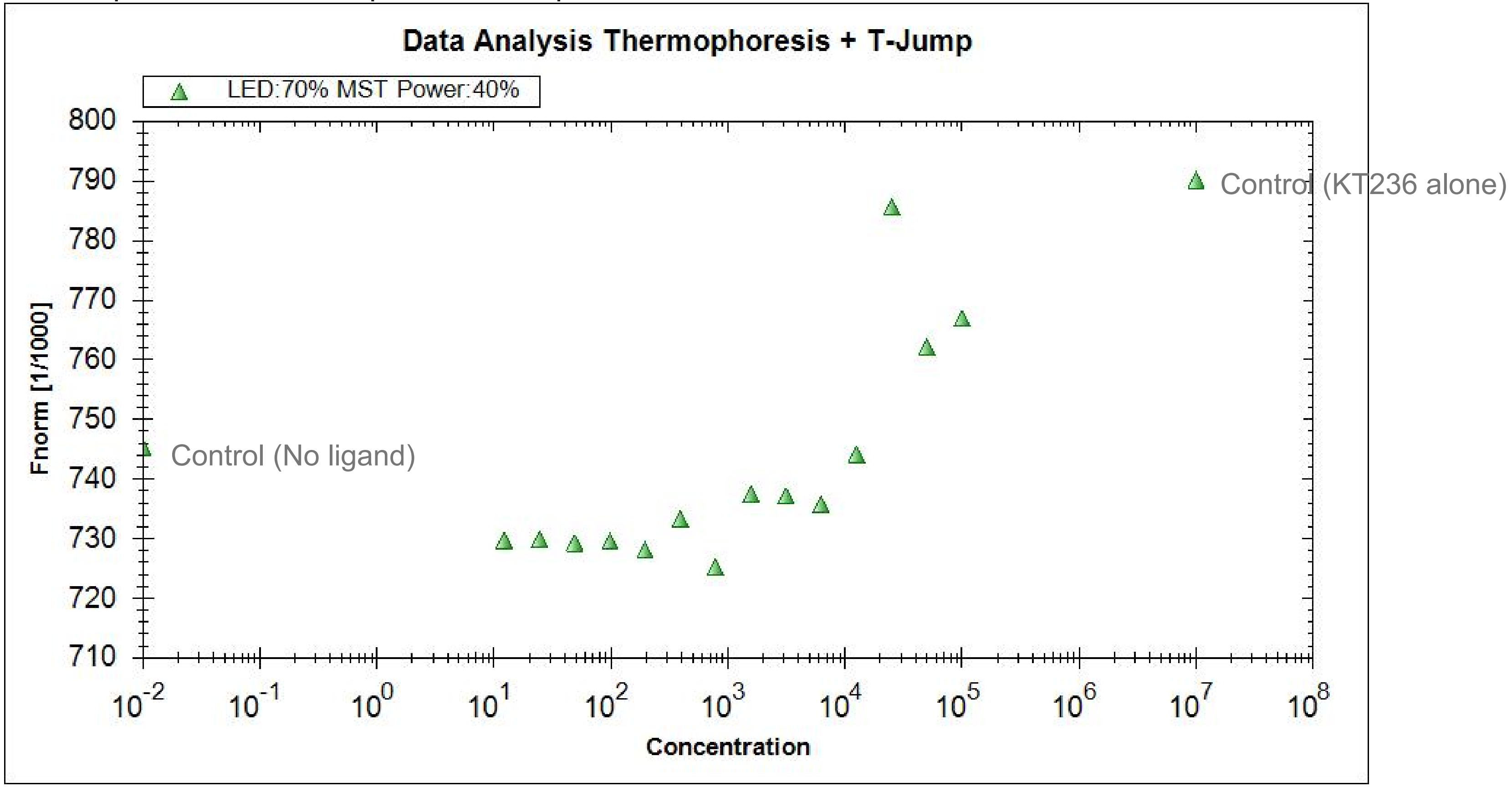

Data

Concentration	Fnorm [1/1000]	
0.01	745.34	Control (No ligand, KT236 (10nM) + IRE1 (180 nM))
12.21	729.70	
24.41	729.90	
48.83	729.29	
97.66	729.57	
195.31	728.13	
390.63	733.40	
781.25	725.22	
1562.50	737.47	
3125.00	737.16	
6250.00	735.68	
12500.00	744.12	
25000.00	785.67	
50000.00	762.25	
100000.00	767.01	
10000000.00	790.19	Control (KT236 alone, 10 nM)

Report

Ntp-Filename: XG4-014_MODIFIED_CONCENTRATION_.ntp
Experiment Name: XG4-014_Qe_replicate3 2/23/2023 12:46:05 PM
MST Power: 40% , LED Power: 70%

Normalized Fluorescence Timetrace

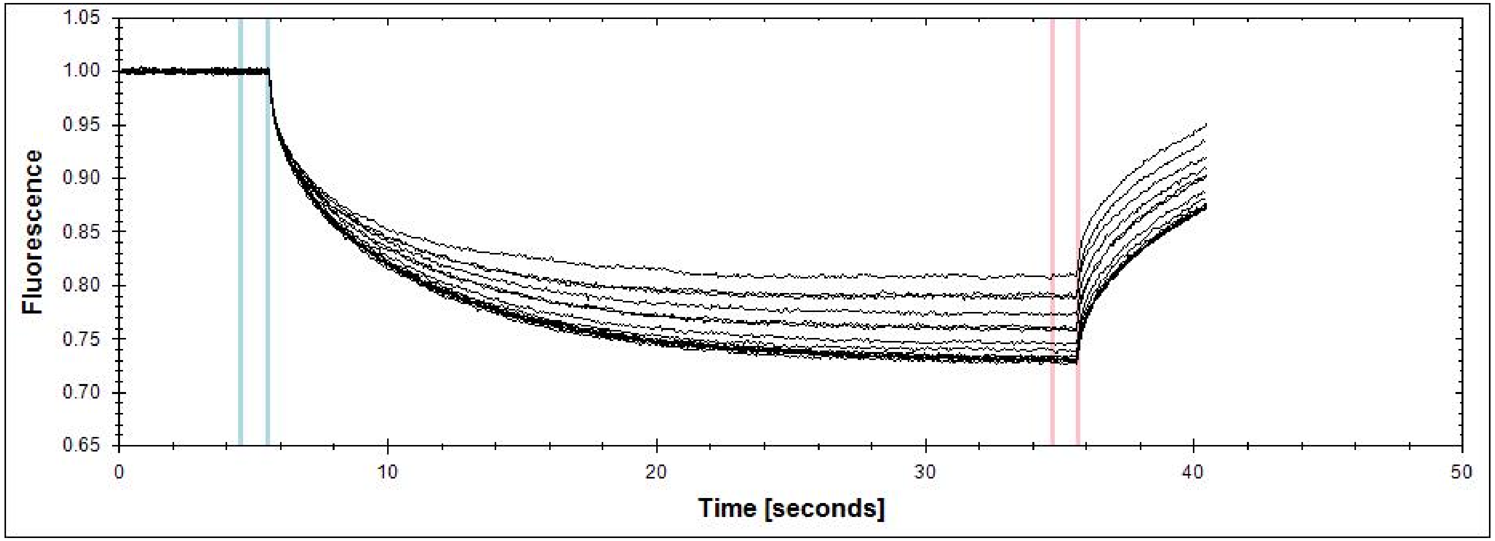

hot region: HotStart=4.55 HotLength=0.97
cold region ColdStart=34.72 ColdLength=0.97

Capillary-Scan: 0

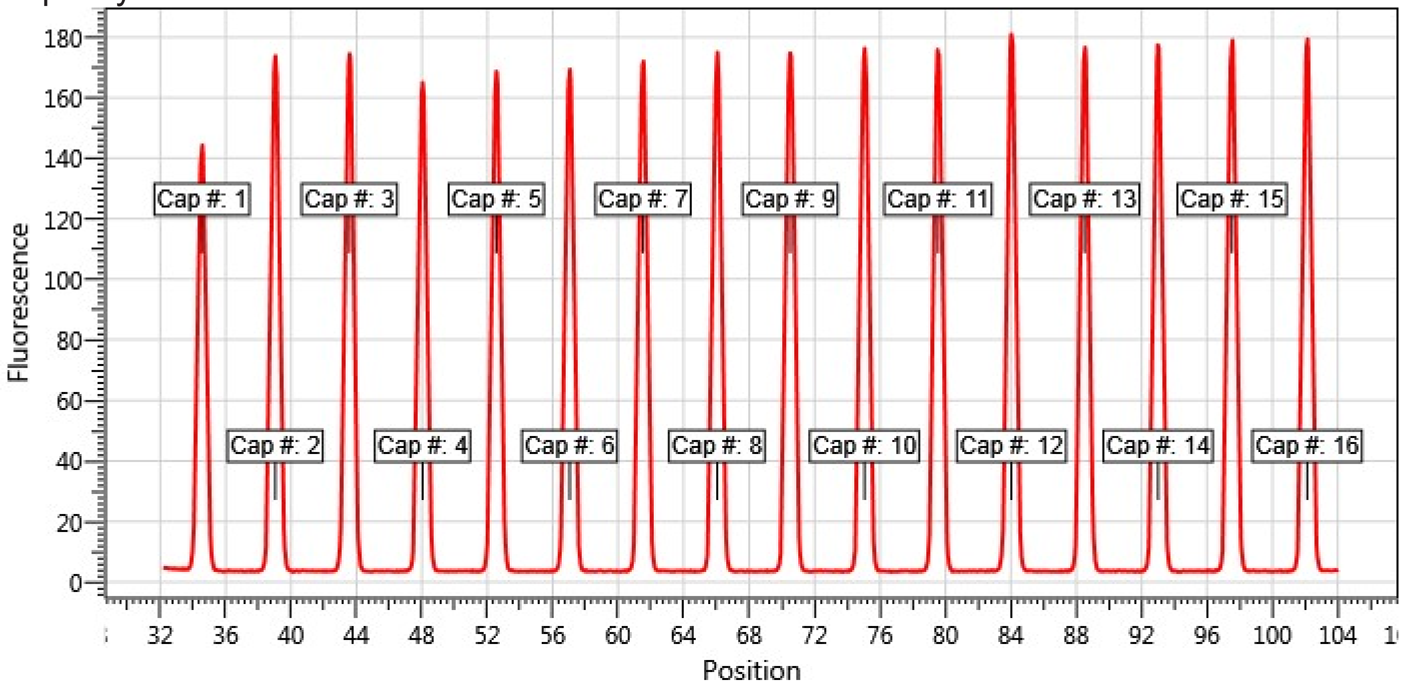

Experiments measured with this Cap-Scan

XG4-014_Qe_replicate3

Thermophoresis with Temperature Jump

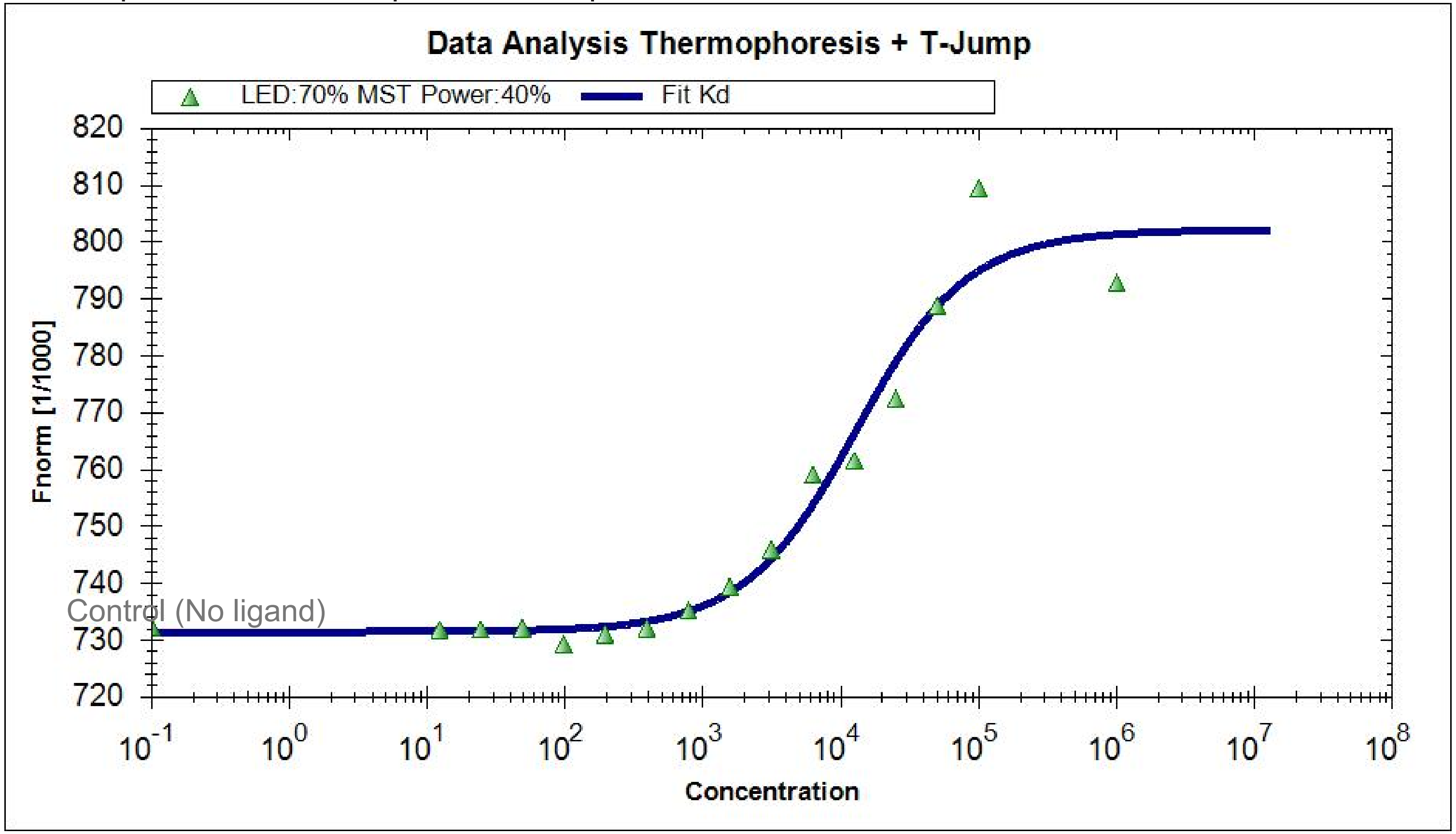

Fitting for Kd Formular

Fitted Parameter	Fitted Value
Dissociation Constant	10900+/-807
Fluo.Conc	3830
Bound	802.18
Unbound	731.49
Amplitude	70.69

Kd Formula (law of mass action)

f(c) = unbound + (bound-unbound) / 2 * (FluoConc + c + Kd - Sqrt((FluoConc + c + Kd)^2 - 4*FluoConc*c)

Data

Concentration	Fnorm [1/1000]	
0.10	732.41	Control (No ligand, KT236 (10nM) + IRE1 (180 nM))
12.21	731.88	
24.41	731.95	
48.83	732.15	
97.66	729.35	
195.31	731.10	
390.63	732.18	
781.25	735.23	
1562.50	739.45	
3125.00	746.04	
6250.00	759.15	
12500.00	761.57	
25000.00	772.49	
50000.00	788.89	
100000.00	809.60	
1000000.00	792.82	Control (KT236 alone, 10 nM)

Report

Ntp-Filename: XG4-012
Experiment Name: Qi_10um 2/21/2023 5:33:25 PM
MST Power: 40% , LED Power: 70%

Normalized Fluorescence Timetrace

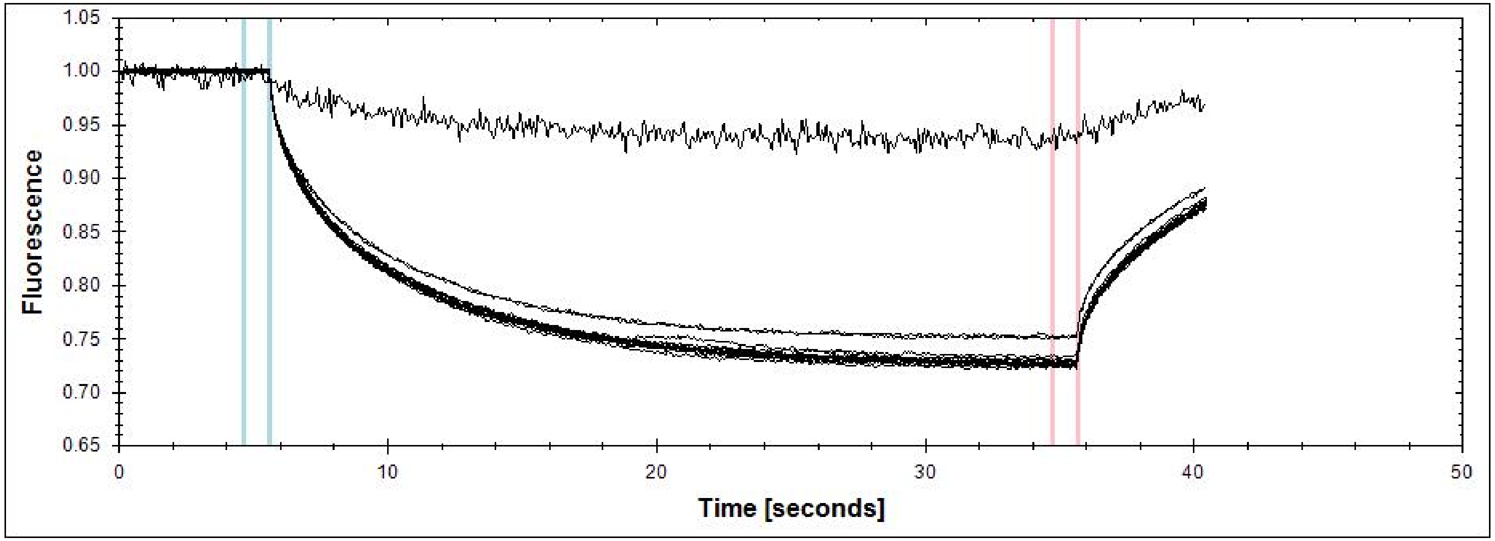

hot region: HotStart=4.62 HotLength=0.97
cold region ColdStart=34.72 ColdLength=0.97

Capillary-Scan: 0

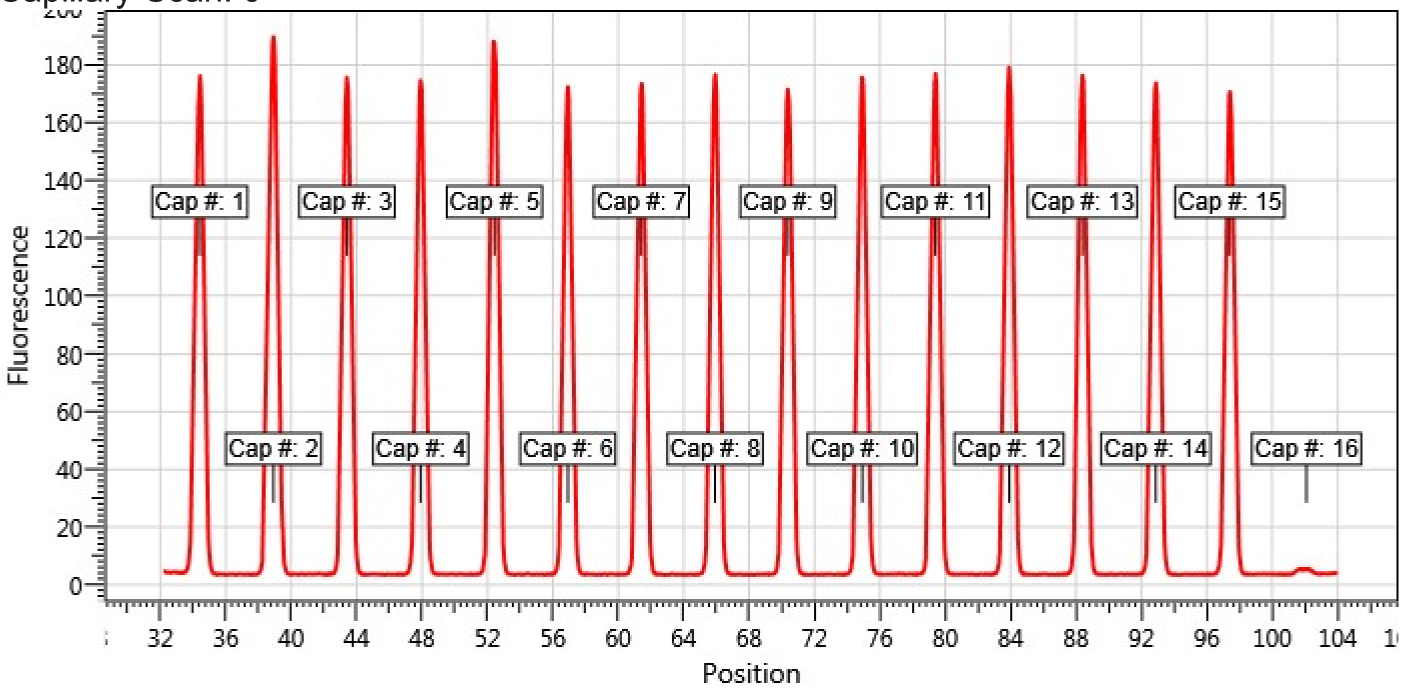

Experiments measured with this Cap-Scan

XG4-014_Qi_replicate3

Thermophoresis with Temperature Jump

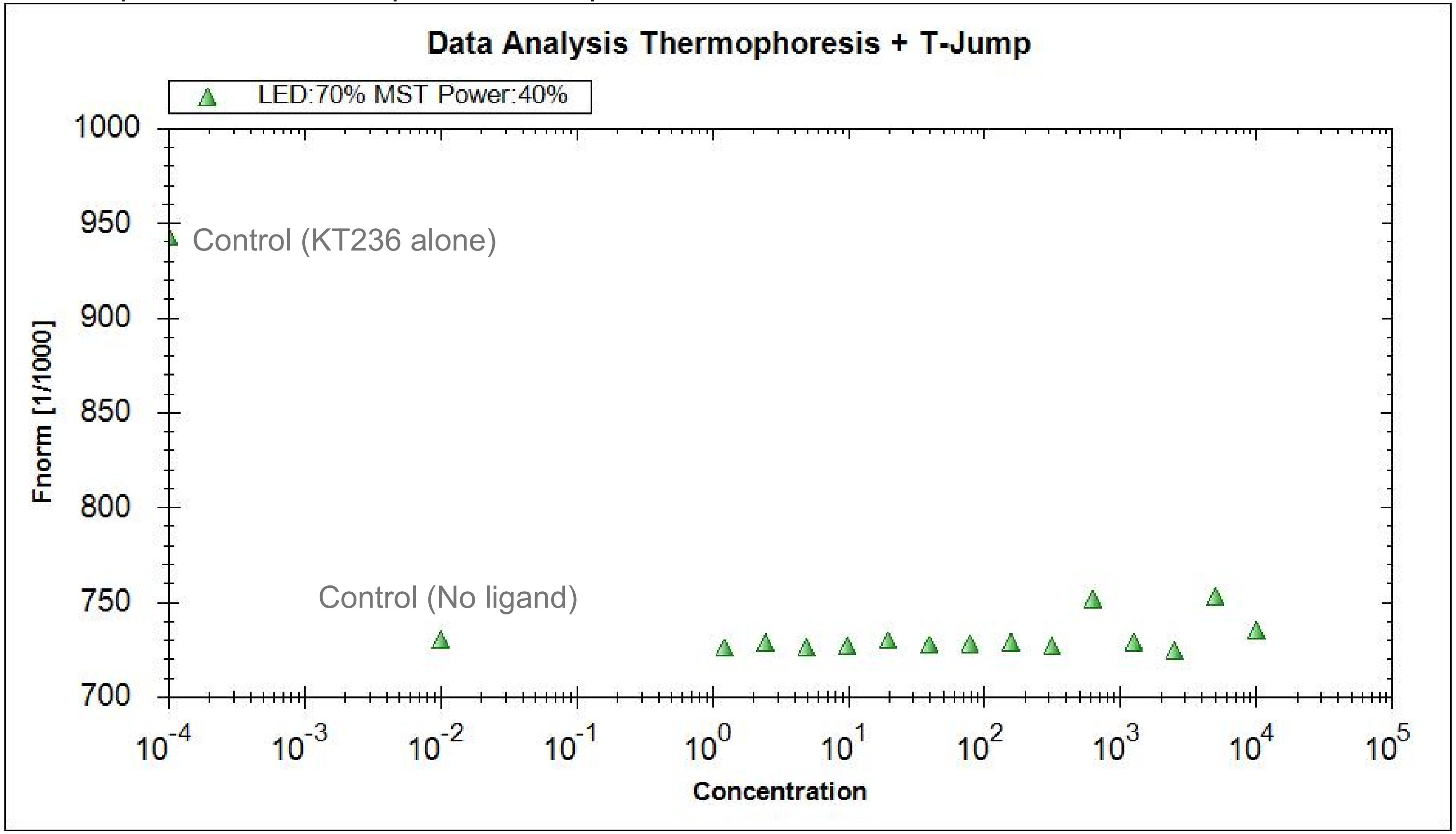

Data

Concentration	Fnorm [1/1000]	
0.00	943.06	Control (KT236 alone, 10 nM)
0.01	730.64	Control (No ligand, KT236 (10nM) + IRE1 (180 nM))
1.22	726.11	
2.44	728.75	
4.88	726.36	
9.77	727.04	
19.53	730.11	
39.06	727.89	
78.13	728.18	
156.25	729.11	
312.50	727.09	
625.00	752.09	
1250.00	729.14	
2500.00	724.86	
5000.00	753.04	
10000.00	735.22	

Report

Ntp-Filename: XG4-013
Experiment Name: Qi_10uM_replicate2 2/22/2023 2:41:45 PM
MST Power: 40% , LED Power: 70%

Normalized Fluorescence Timetrace

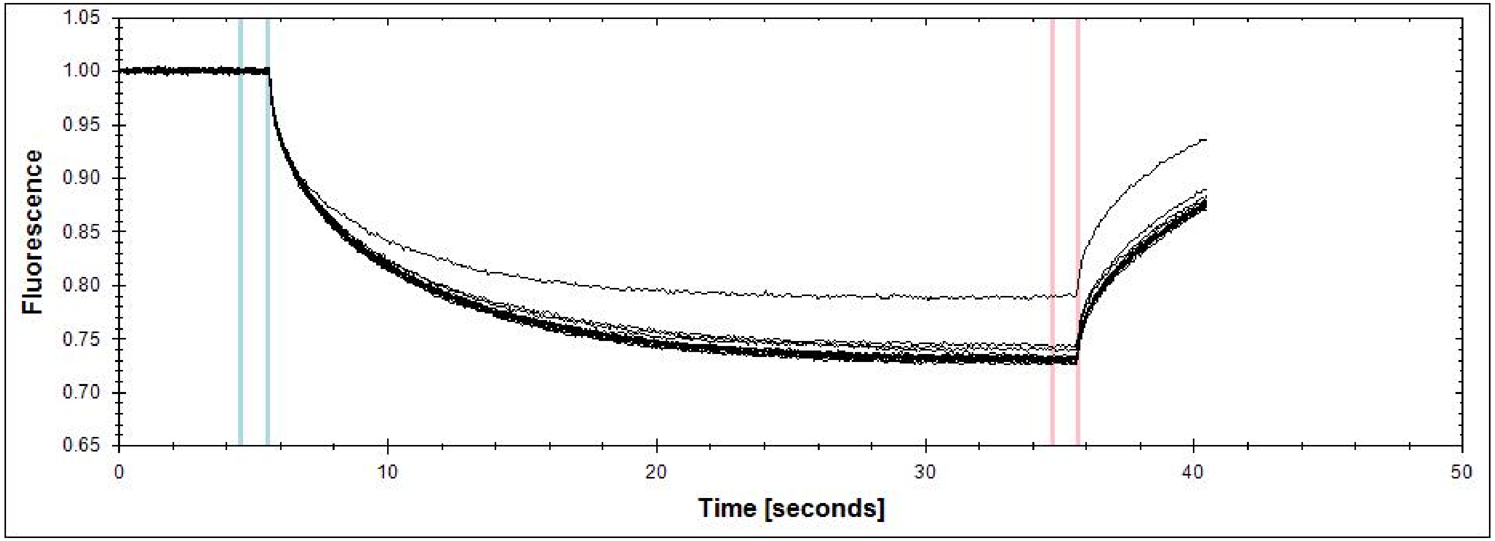

hot region: HotStart=4.55 HotLength=0.97
cold region ColdStart=34.72 ColdLength=0.97

Capillary-Scan: 0

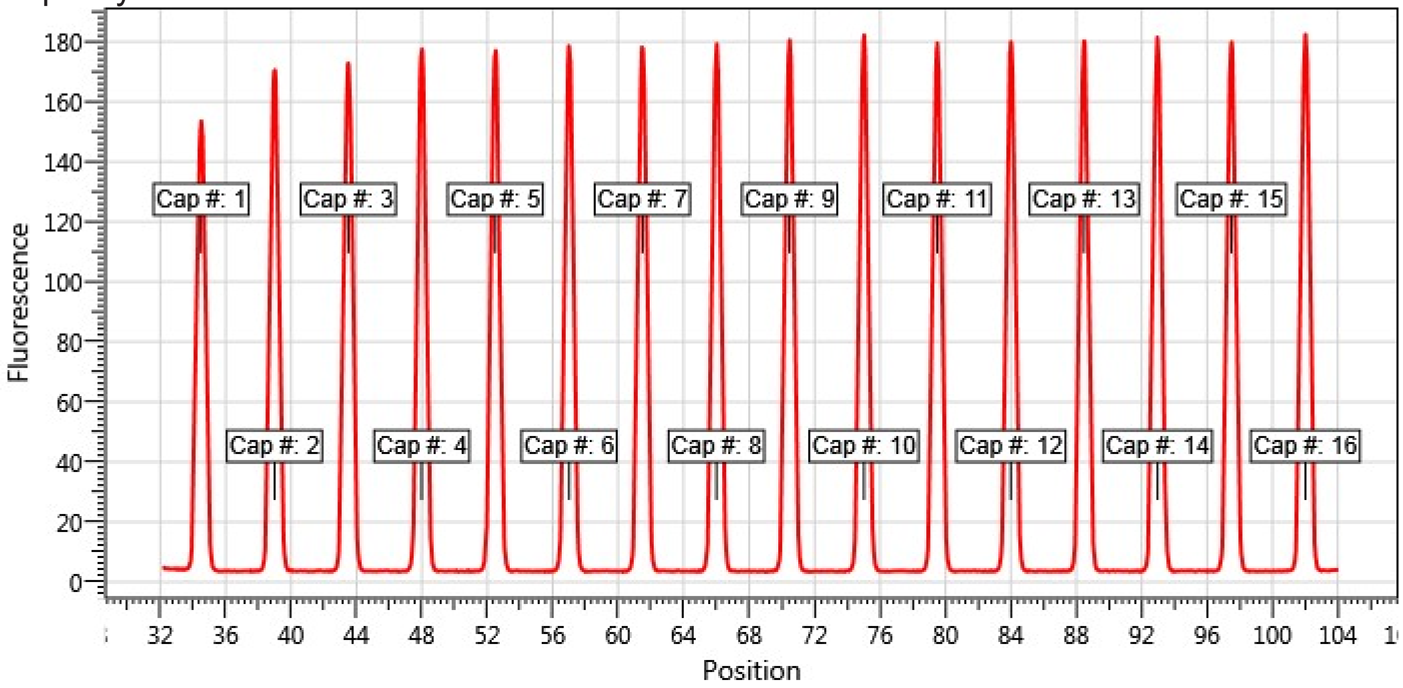

Experiments measured with this Cap-Scan

XG4-014_Qi_replicate3

Thermophoresis with Temperature Jump

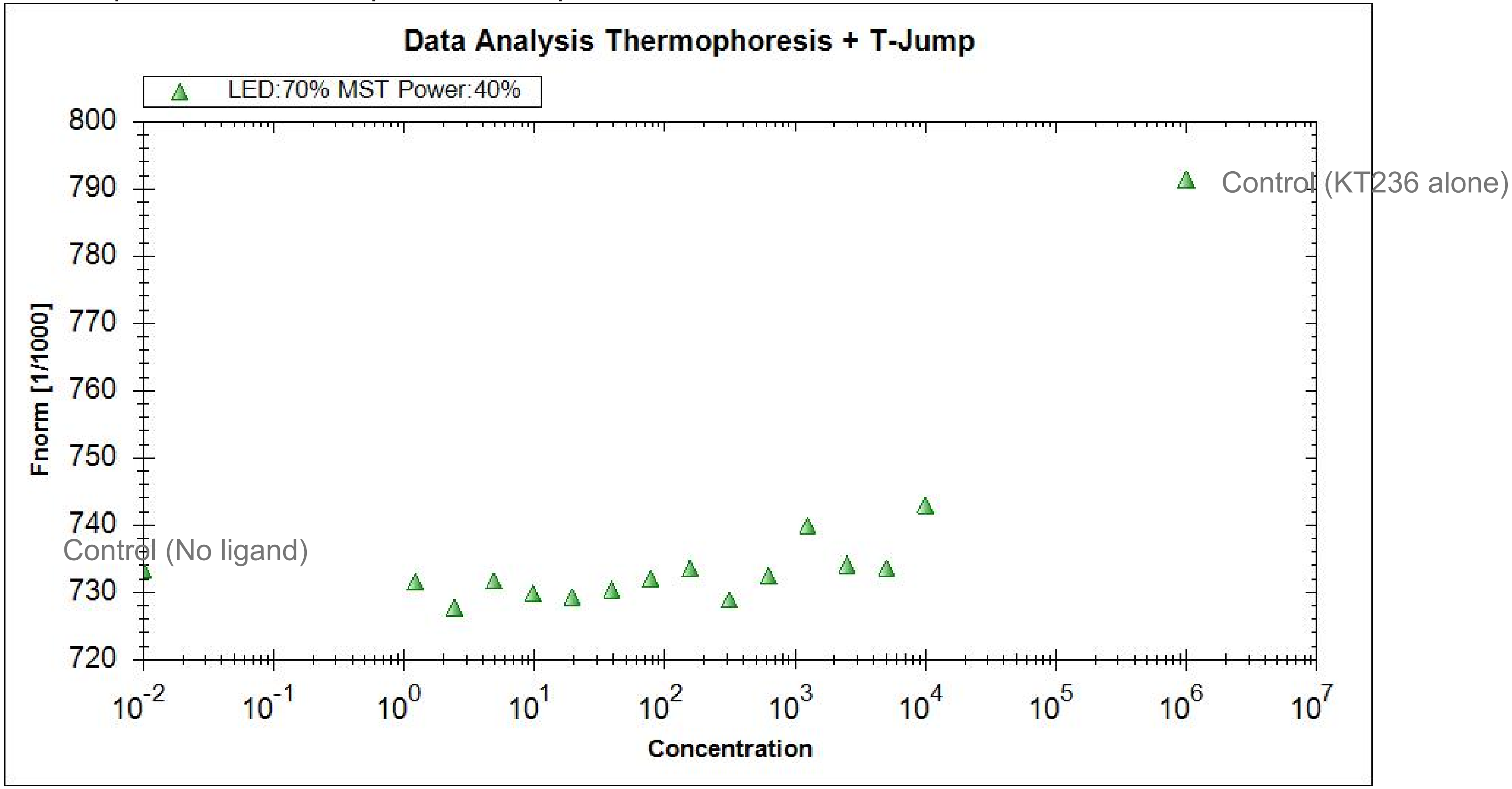

Data

Concentration	Fnorm [1/1000]	
0.01	733.31	— Control (No ligand, KT236 (10nM) + IRE1 (180 nM))
1.22	731.66	
2.44	727.77	
4.88	731.73	
9.77	729.81	
19.53	729.29	
39.06	730.41	
78.13	732.01	
156.25	733.63	
312.50	728.96	
625.00	732.48	
1250.00	739.96	
2500.00	734.11	
5000.00	733.66	
10000.00	743.01	
1000000.00	791.42	— Control (KT236 alone, 10 nM)

Report

Ntp-Filename: XG4-014
Experiment Name: XG4-014_Qi_replicate3 2/23/2023 1:16:35 PM
MST Power: 40% , LED Power: 70%

Normalized Fluorescence Timetrace

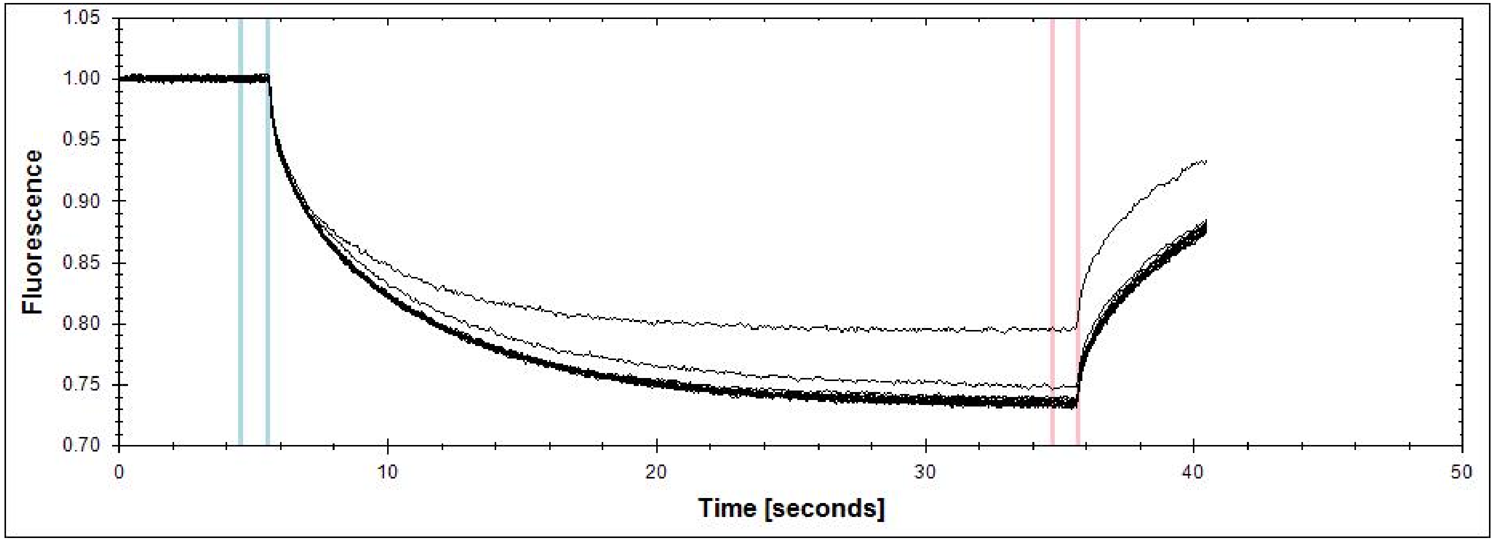

hot region: HotStart=4.55 HotLength=0.97
cold region ColdStart=34.72 ColdLength=0.97

Capillary-Scan: 0

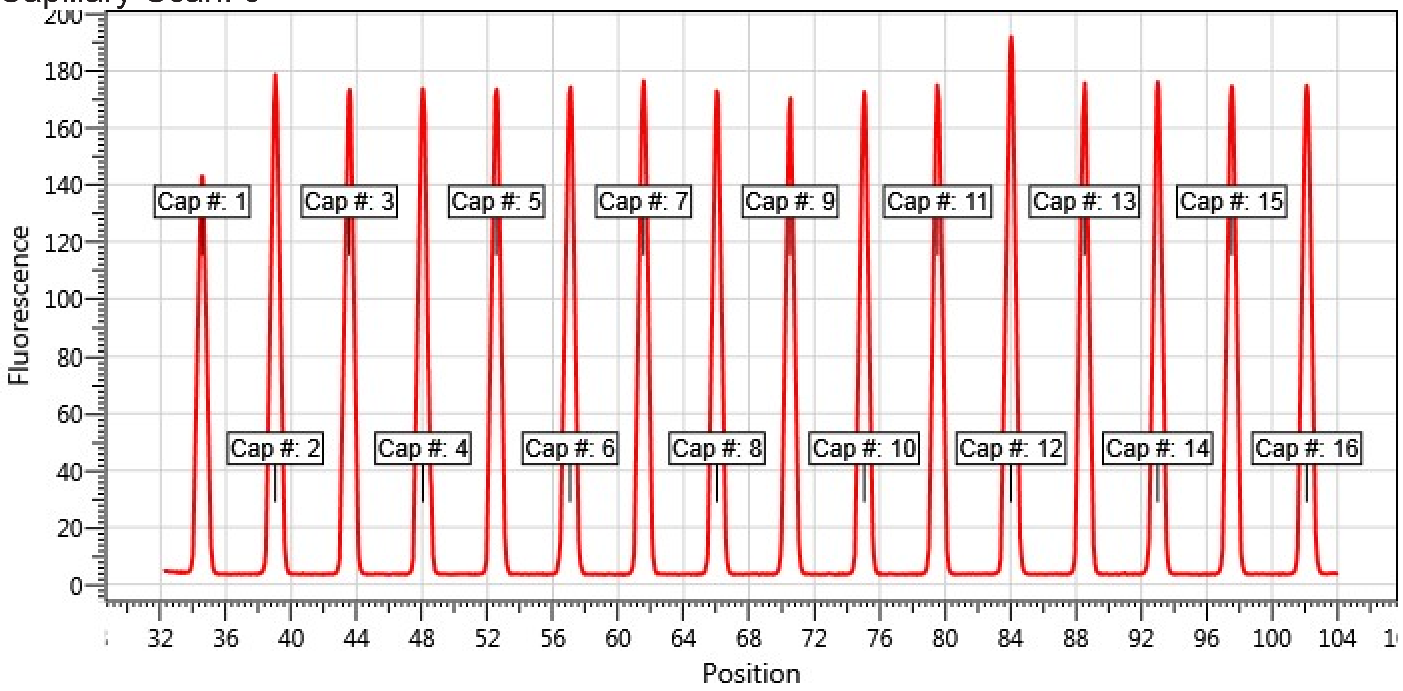

Experiments measured with this Cap-Scan

XG4-014_Qi_replicate3

Thermophoresis with Temperature Jump

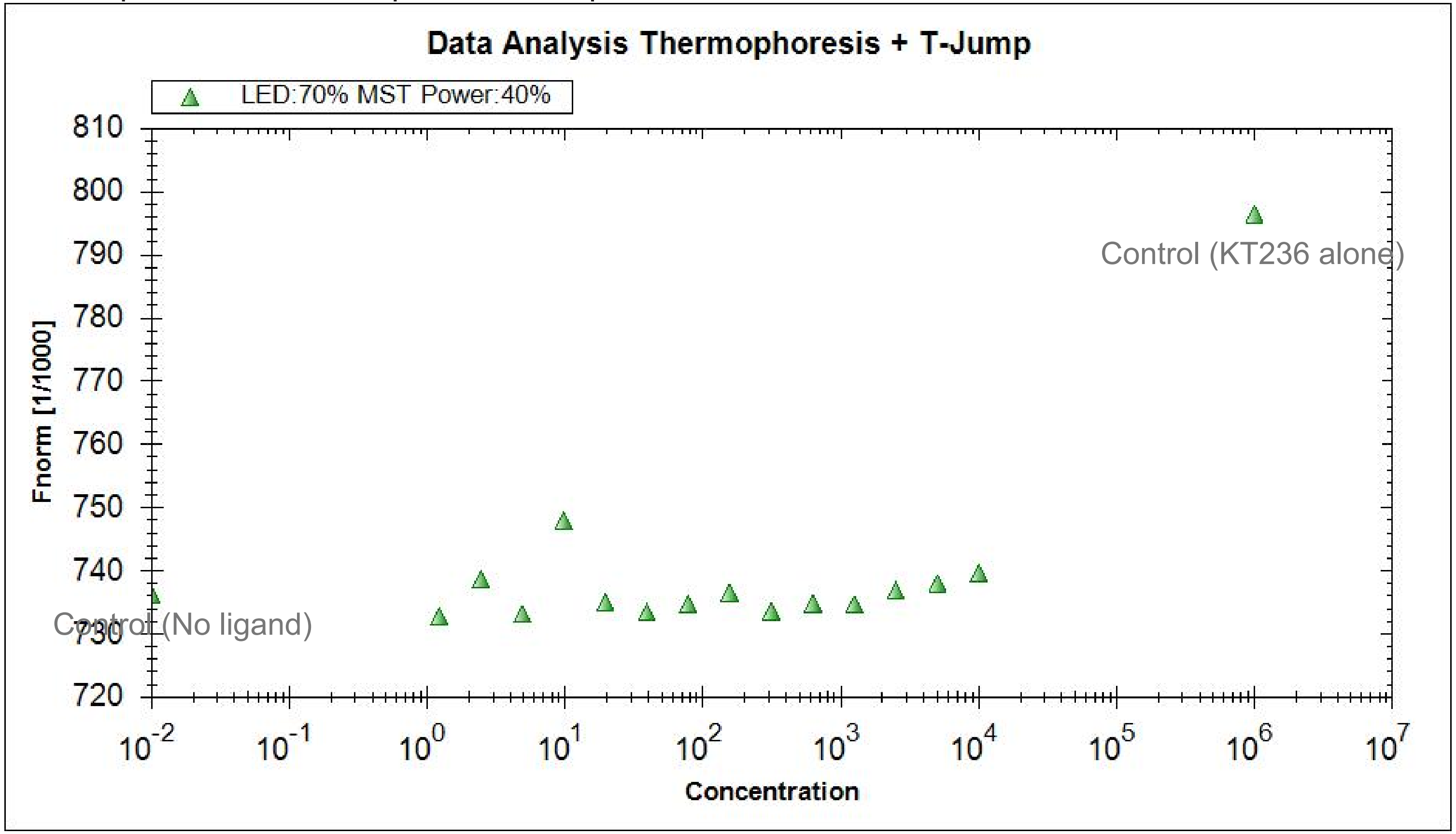

Data

Concentration	Fnorm [1/1000]	
0.01	736.21	Control (No ligand, KT236 (10nM) + IRE1 (180 nM))
1.22	732.83	
2.44	738.65	
4.88	733.19	
9.77	747.97	
19.53	735.09	
39.06	733.45	
78.13	734.68	
156.25	736.58	
312.50	733.55	
625.00	734.85	
1250.00	734.69	
2500.00	736.90	
5000.00	738.02	
10000.00	739.68	
1000000.00	796.50	Control (KT236 alone, 10 nM)
