# Supplementary material for: Different binding modalities of quercetin to inositol-requiring enzyme 1 of S. cerevisiae and human lead to opposite regulation
Source: Commun Chem. 2024 Jan 5;7:6. doi: 10.1038/s42004-023-01092-0 (PMC10767055; doi:10.1038/s42004-023-01092-0)
Supplement: Supplementary file 5 — Supplementary Data 3 [file 42004_2023_1092_MOESM5_ESM.pdf]

Report

Ntp-Filename: XG4-016
Experiment Name: Protein titration n2 2/28/2023 10:28:26 AM
MST Power: 40% , LED Power: 60%

Normalized Fluorescence Timetrace

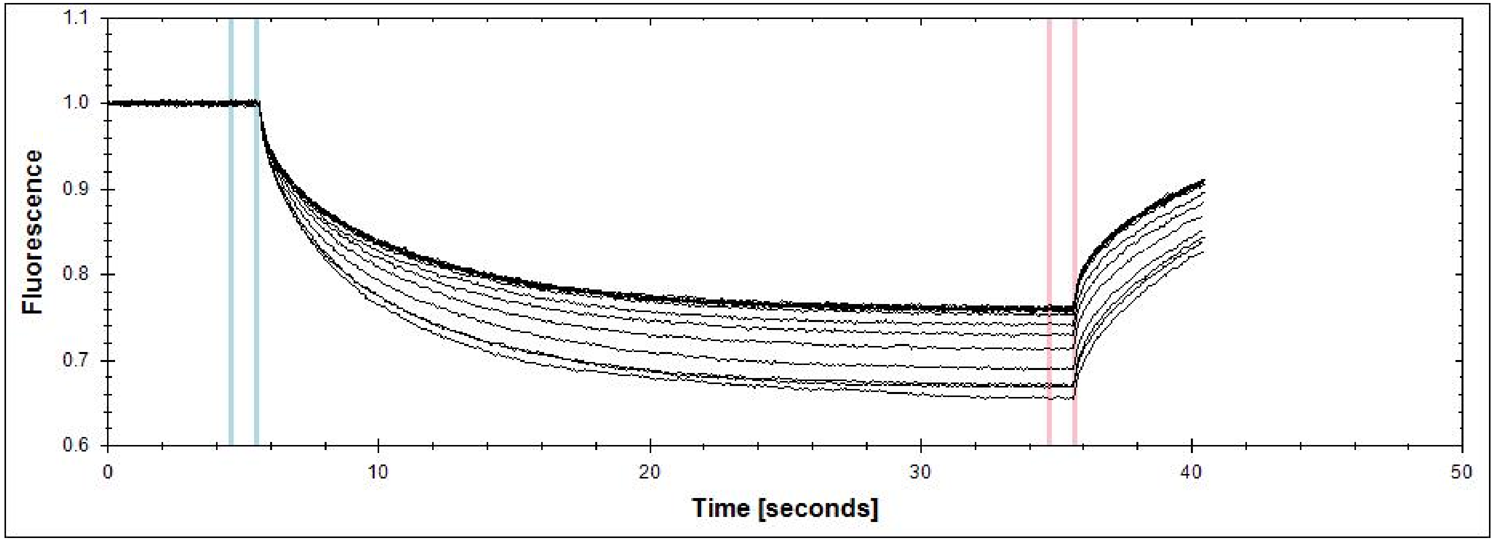

hot region: HotStart=4.55 HotLength=0.97
cold region ColdStart=34.72 ColdLength=0.97

Capillary-Scan: 0

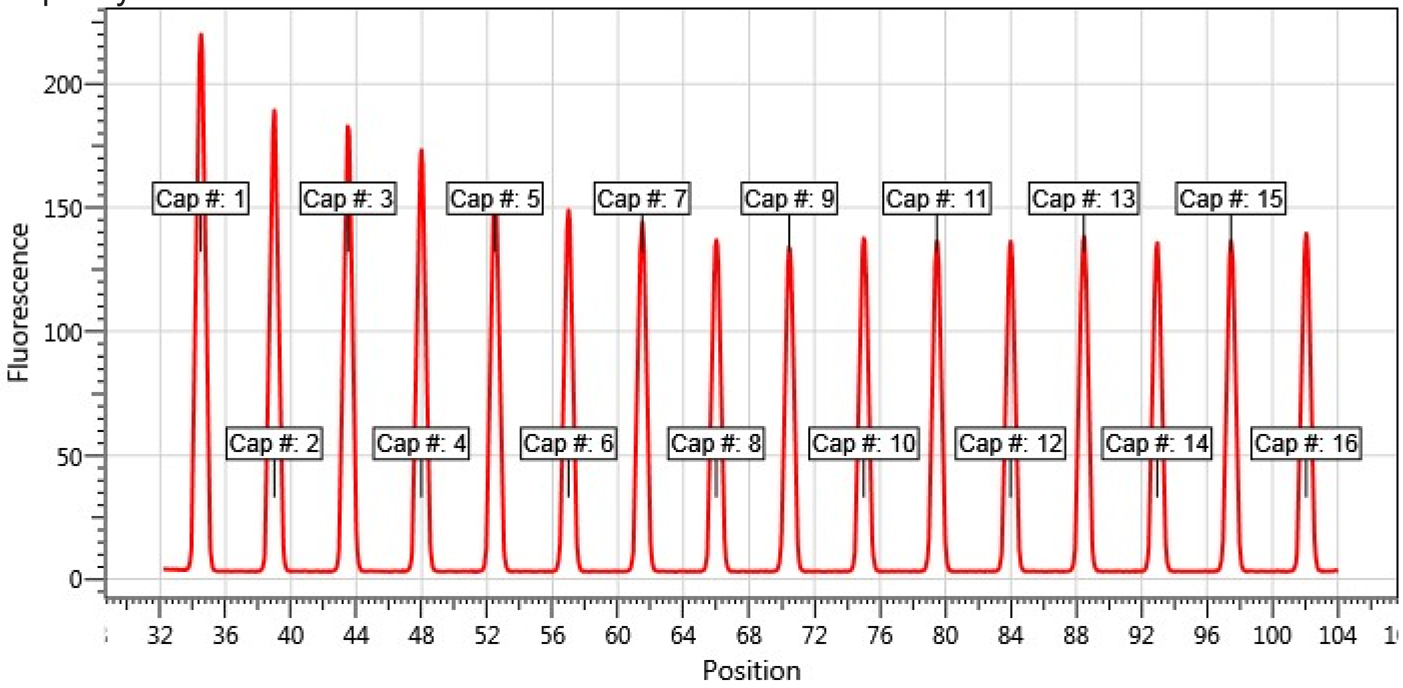

Experiments measured with this Cap-Scan

XG4-010_0.30ug/ul_protein_stock ;XG4-010_0.30ug/ul_protein_stock

Thermophoresis with Temperature Jump

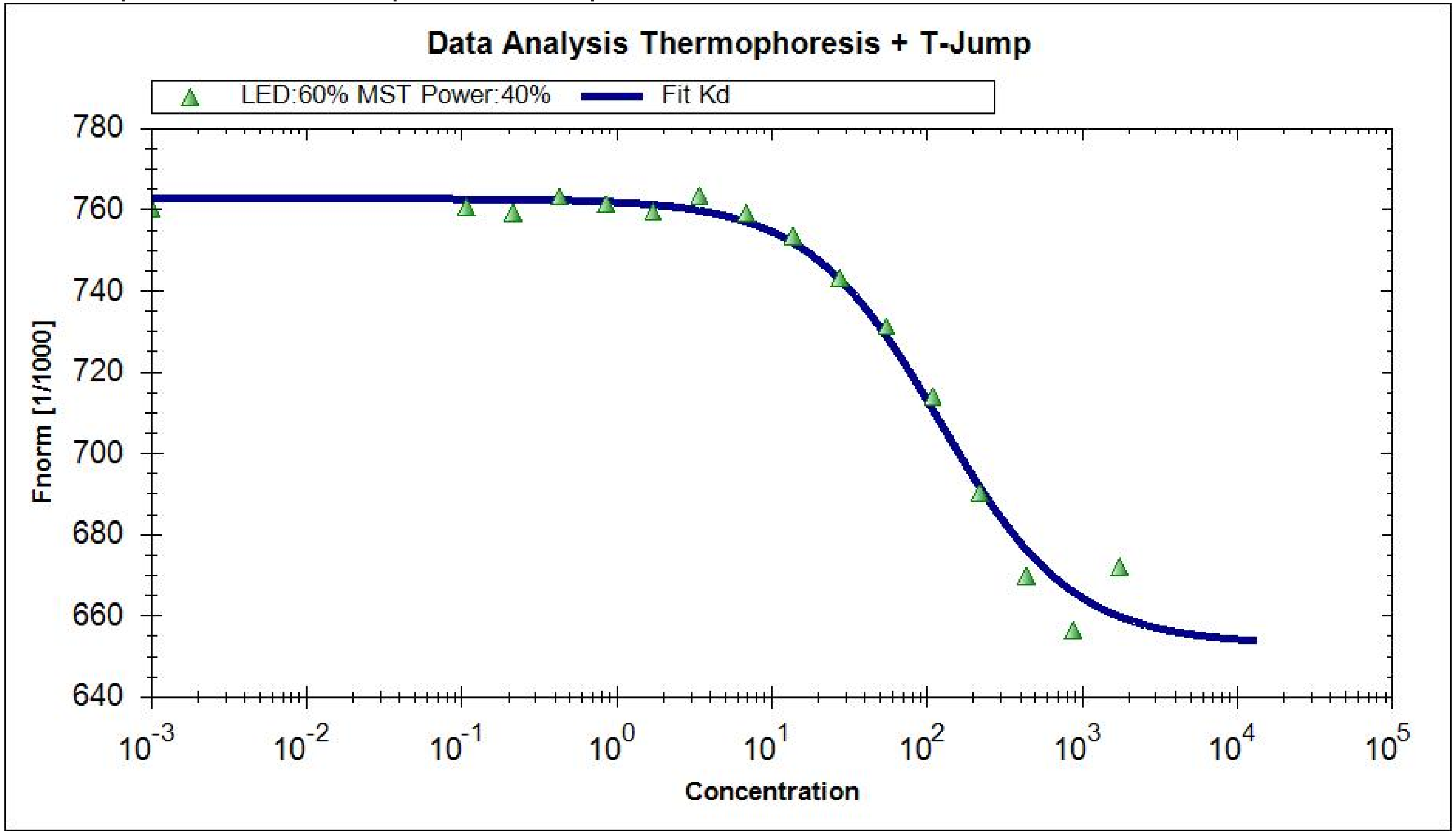

Fitting for Kd Formular

Fitted Parameter	Fitted Value
Dissociation Constant	116+/-4.54
Fluo.Conc	10
Bound	652.98
Unbound	762.73
Amplitude	109.75

Kd Formula (law of mass action)

f(c) = unbound + (bound-unbound) / 2 * (FluoConc + c + Kd - Sqrt((FluoConc + c + Kd)^2 - 4*FluoConc*c)

Data

Concentration	Fnorm [1/1000]
0.00	760.43
0.11	760.69
0.21	759.42
0.43	763.34
0.85	761.37
1.70	759.47
3.41	763.58
6.82	759.30
13.63	753.71
27.27	743.21
54.53	731.29
109.06	714.10
218.13	690.27
436.25	669.98
872.50	656.53
1745.00	672.13

Report

Ntp-Filename: XG4-016
Experiment Name: Protein titration n3 2/28/2023 10:53:23 AM
MST Power: 40% , LED Power: 60%

Normalized Fluorescence Timetrace

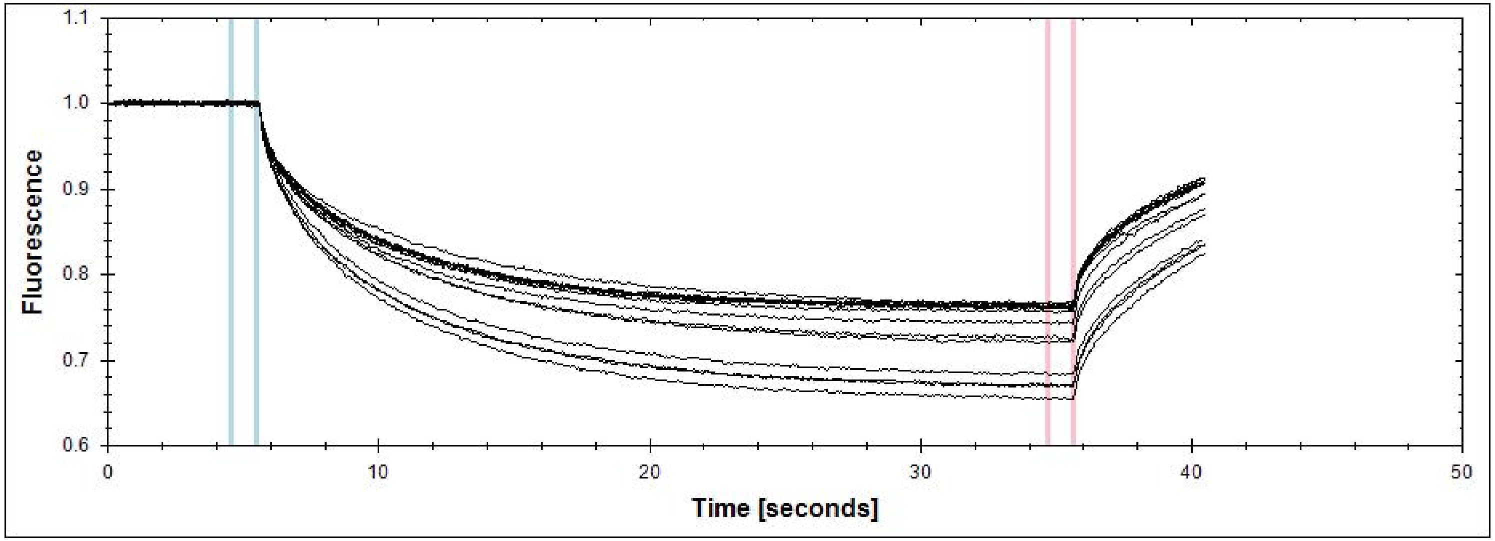

hot region: HotStart=4.55 HotLength=0.97
cold region ColdStart=34.65 ColdLength=0.97

Capillary-Scan: 0

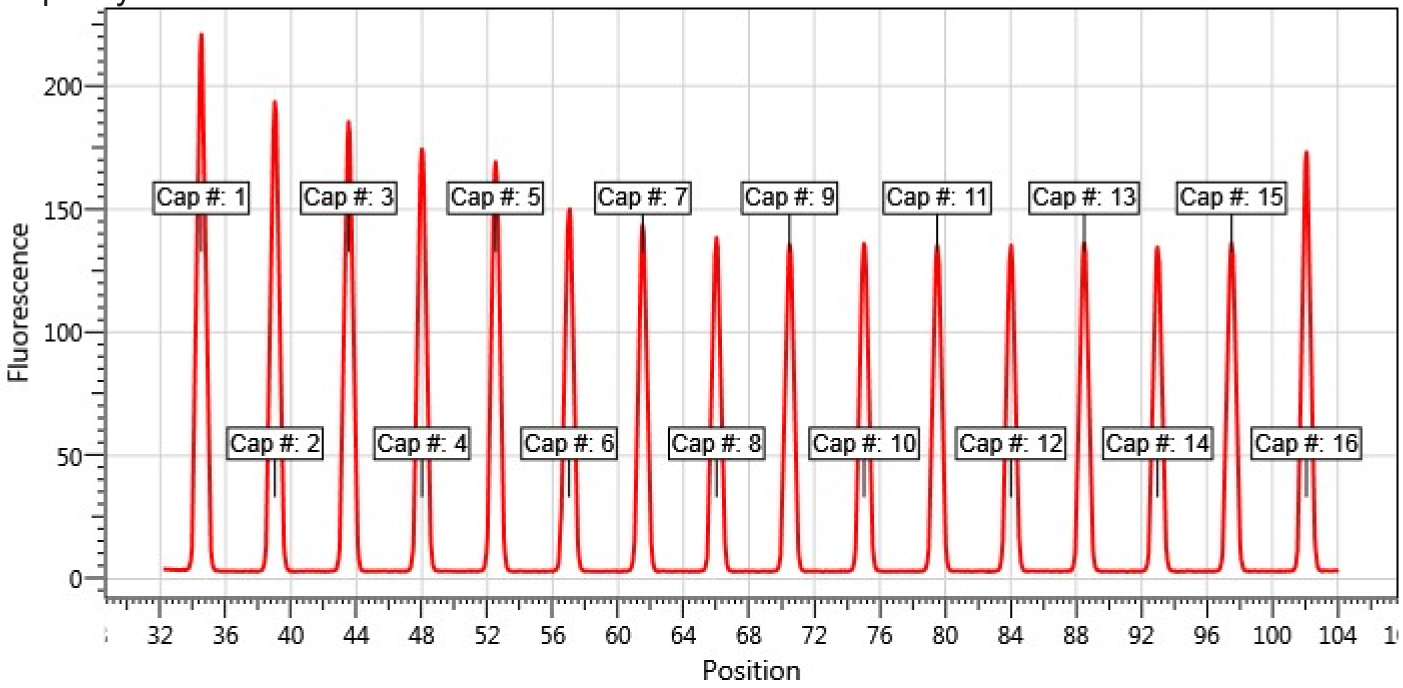

Experiments measured with this Cap-Scan

XG4-010_0.30ug/ul_protein_stock ;XG4-010_0.30ug/ul_protein_stock

Thermophoresis with Temperature Jump

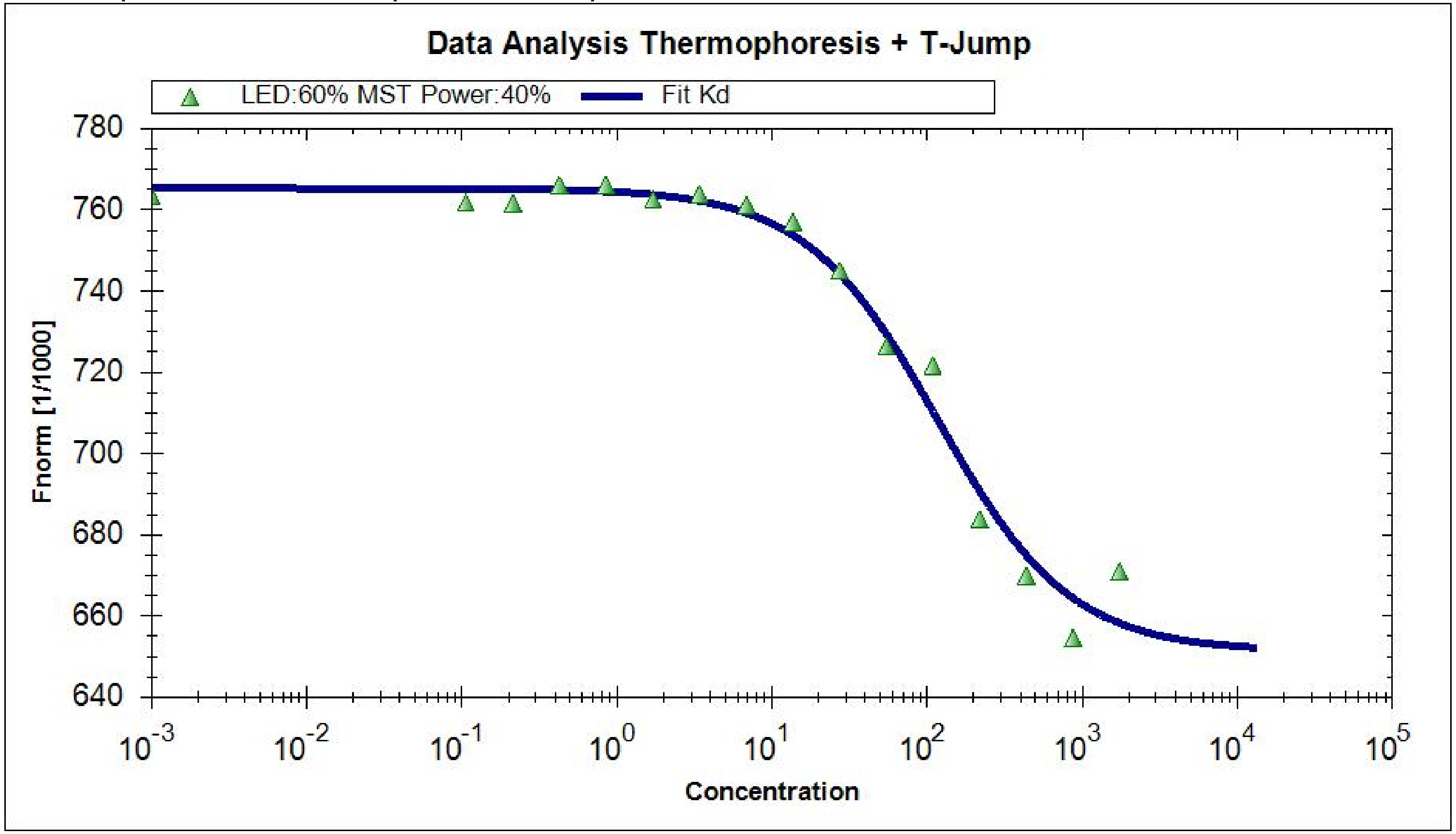

Fitting for Kd Formular

Fitted Parameter	Fitted Value
Dissociation Constant	112+/-6.51
Fluo.Conc	10
Bound	651.25
Unbound	765.32
Amplitude	114.07

Kd Formula (law of mass action)

f(c) = unbound + (bound-unbound) / 2 * (FluoConc + c + Kd - Sqrt((FluoConc + c + Kd)^2 - 4*FluoConc*c)

Data

Concentration	Fnorm [1/1000]
0.00	763.51
0.11	761.77
0.21	761.68
0.43	766.09
0.85	766.20
1.70	762.51
3.41	763.90
6.82	761.31
13.63	757.01
27.27	745.03
54.53	726.35
109.06	721.73
218.13	683.74
436.25	669.84
872.50	654.70
1745.00	670.95
